# Regioselective C3–C4 difunctionalization of 4-bromocoumarins *via* Pd/norbornene catalysis

**DOI:** 10.1039/d5ra05007c

**Published:** 2025-09-22

**Authors:** Aynaz Feyzi, Farnaz Jafarpour

**Affiliations:** a School of Chemistry, College of Science, University of Tehran 14155-6455 Tehran Iran Jafarpour@ut.ac.ir

## Abstract

The difunctionalization of arenes *via* Catellani-type reactions has predominantly relied on aryl iodides and has faced persistent limitations with dual bromide systems, particularly with benzyl bromides, which are susceptible to oxidation reactions. Herein, we present a Pd-catalyzed/norbornene-mediated vicinal difunctionalization of 4-bromocoumarins *via* sequential alkylation and vinylation using dual bromide partners—a longstanding challenge in Catellani chemistry.

## Abstract 2

Catellani-type vicinal difunctionalization of 4-bromocoumarins *via* sequential alkylation and vinylation reactions.

## Graphical abstract

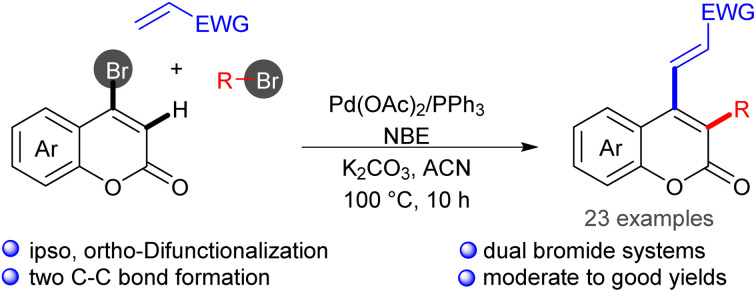

The Catellani reaction, introduced in 1997, highlights the transformative power of transition metal catalysis in modern organic synthesis.^1^ Based on palladium/norbornene cooperative catalysis, this strategy enables vicinal difunctionalization of (hetero)arenes through a one-pot sequence involving sequential *ortho* and *ipso*-functionalization of aryl halides.^2^ Its ability to incorporate diverse functional groups into complex scaffolds from simple precursors has made it a versatile platform with wide-ranging applications in medicinal chemistry, materials science, and natural product synthesis.^3^

Despite extensive development of aryl Catellani reactions, aryl iodides have remained the predominant substrates due to their superior oxidative addition kinetics. In contrast, aryl bromides, although more abundant and synthetically accessible, are considerably under-explored in this context. Their reduced reactivity toward Pd(0) limits their utility, especially in complex transformations requiring fine-tuned reactivity and intermediate stability.^3*d*,4^ Mechanistic studies have shown that the Catellani sequence involves two key oxidative additions: first, the aryl halide adds to Pd(0), and second, the electrophile adds to the aryl–norbornyl–palladacycle (ANP) intermediate. Premature oxidative addition of the electrophile can disrupt this sequence, often resulting in β-hydride elimination byproducts.^2*e*,4*a*,5^ These deviations from the intended pathway are illustrated in Scheme 1A, where early electrophile activation or misdirected engagement of the aryl halide leads to off-cycle byproducts.

**Scheme 1:**
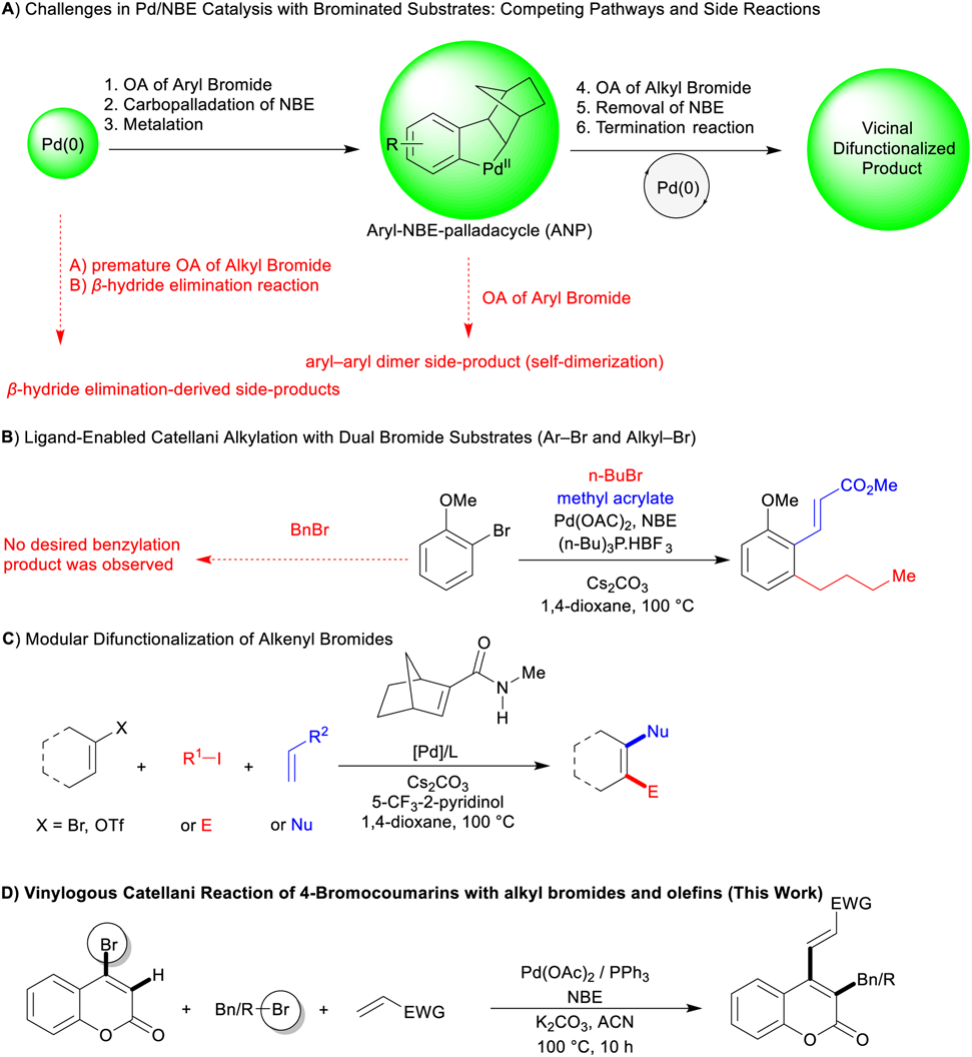
Mechanistic considerations and strategic ligand/NBE modulations in Pd/norbornene catalysis with brominated substrates.

According to literature precedents,^4*a*^ in traditional intermolecular Catellani-type reactions, highly reactive alkyl iodides can outcompete aryl bromides for oxidative addition to Pd(0), disrupting the desired catalytic sequence. When alkyl bromides are used alongside aryl iodides, iodide additives like NaI are often necessary to boost reactivity.^6^ However, dual bromide systems remain rare, as both components exhibit intrinsically low oxidative addition rates. A noteworthy exception is Dong's report on a unique difunctionalization involving 2-bromoanisole and butyl bromide, facilitated by the electron-rich ligand tris(4-methoxyphenyl)phosphine. Even under these optimized conditions, benzyl bromides remained incompatible, highlighting the ongoing difficulty of achieving selective alkylation in such systems (Scheme 1B).^4*a*^

Seeking to overcome these substrate limitations, we turned our attention to the emerging class of vinylic-type Catellani reactions, which utilize highly reactive partially aromatic and non-aromatic scaffolds in place of traditional aryl halides. This concept was first introduced by Lautens in 2006 using iodinated uracils,^7^ and later developed by Yamamoto^8^ and Zhou^9^ through the use of iodinated 2-quinolones and iodinated 2-pyridones and uracils, respectively. In a complementary direction, Dong expanded this platform to include brominated non-aromatic substrates, notably demonstrating the difunctionalization of alkenyl bromides with *N*-methyl amide substitutions to hinder cyclopropanation and fine-tune reactivity under Pd/NBE catalysis (Scheme 1C).^10^ Given these advantages and modest success of dual bromide systems in Pd/NBE catalysis, coupled with the underutilized potential of vinylic bromide substrates, we began to investigate whether 4-bromocoumarins could be effective in this context. Their conjugated benzene–lactone framework promotes oxidative addition and supports regioselective reactivity, offering both electronic activation and structural rigidity—features ideal for engaging unactivated alkyl bromides, in Catellani-type chemistry. Beyond their synthetic appeal, coumarins are privileged scaffolds in medicinal chemistry, exhibiting diverse bioactivities such as anticoagulant,^11^ antibacterial,^12^ anti-inflammatory,^13^ antitumor^14^ and antioxidant^15^ effects. Clinically approved derivatives like warfarin and acenocoumarol underscore their pharmacological relevance,^16^ while their photophysical properties enable applications in OLEDs, optical brighteners, and fluorescent probes.^17^ Herein, we report the Pd/NBE-catalyzed vicinal alkylation and vinylation of 4-bromocoumarins using electron-deficient olefins and unactivated alkyl bromides under standard catalytic conditions, without the need for specialized ligands or norbornene derivatives (Scheme 1D).

We began our investigation by employing benzyl bromide as the alkylating agent to assess its reactivity with 4-bromocoumarin in comparison to traditional aryl bromides, with the goal of overcoming longstanding challenges associated with dual bromide systems in Catellani-type reactions. The initial phase of our study involved the reaction of 4-bromocoumarin (1a) with benzyl bromide (2a) as the electrophile and ethyl acrylate (3a) as the terminating nucleophile. Through meticulous parameter optimization, we were able to obtain a reliable reaction condition, which involved the utilization of 5 equiv of 2a, 2 equiv of 3a, 10 mol% of Pd(OAc)_2_, 20 mol% of PPh_3_, 5 equiv of K_2_CO_3_, and 4 equiv of norbornene in ACN as the solvent. Under these optimized conditions, we achieved the desired product 4a with a favorable yield of 65% after conducting the reaction at 100 °C for 10 hours (Table 1, entry 1). Underscoring the critical significance of both palladium and norbornene (NBE), control experiments revealed that the absence of the Pd(OAc)_2_ catalyst or the norbornene mediator led to a complete suppression in synthesizing of the desired product 4a (entries 2 and 3). Notably, the ligand PPh_3_ likely stabilizes the Pd(0) species and facilitates oxidative addition, as evidenced by the diminished yield in its absence (entry 4). The screening of various palladium catalysts confirmed that Pd(OAc)_2_ was the most effective (entries 5 and 6). In a screening to evaluate the effects of various bases, K_2_CO_3_ was replaced with alternatives like NaOAc and Cs_2_CO_3_ (entries 7 and 8). Additionally, after careful evaluation of the tested solvents, ACN was identified as the most suitable choice among 1,4-dioxane, THF, toluene and *etc* (for more information see SI). However, these modifications resulted in a significant decrease in product yield across all experimental conditions (entries 9–11). A significant reduction in product efficiency is observed when the norbornene equivalent and temperature are altered from their standard conditions (entries 12 and 13).

**Table 1:** Optimization of reaction conditions^a^

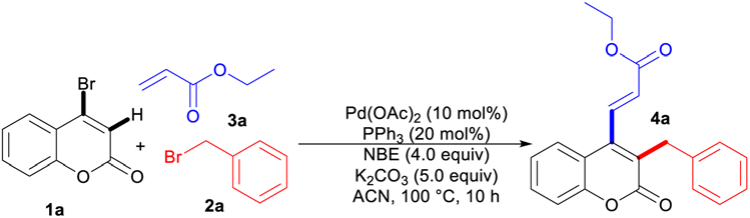		
Entry	Change from standard conditions	Yield
1	Standard conditions	65
2	No Pd catalayst	0
3	No NBE	0
4	No PPh_3_	34
5	PdCl_2_ instead of Pd(OAc)_2_	43
6	Pd(dba)_2_ instead of Pd(OAc)_2_	50
7	NaOAc instead of K_2_CO_3_	33
8	Cs_2_CO_3_ instead of K_2_CO_3_	45
9	1,4-Dioxane instead of ACN	51
10	THF instead of ACN	45
11	Toluene instead of ACN	40
12	NBE (3.0 equiv)	49
13	80 °C	58

a Reaction conditions: 1a (0.1 mmol), 2a (0.5 mmol, 5.0 equiv), 3a (0.2 mmol, 2.0 equiv), Pd(OAc)_2_ (10 mol%), PPh_3_ (20 mol%), norbornene (0.4 mmol, 4.0 equiv), base (0.5 mmol, 5.0 equiv), solvent (1.0 mL), 100 °C, 10 h.

Utilizing the established optimized conditions, we investigated the generality of this three-component domino reaction across a range of bromocoumarins, alkyl bromides, and olefins. As shown in Table 2, different acrylates exhibited favorable performance in the reaction with 4-bromocoumarin and benzyl bromide to afford products 4a–4d in 65% to 75% yields. Following the unambiguous confirmation of the structure of 4d through single-crystal X-ray diffraction (CCDC 2430471), we proceeded to enhance the overall generality of the reaction by introducing greater diversity in both electrophiles and nucleophiles. In this regard, the reactivity of straight-chain alkyl bromides, encompassing both short and long chains, was first investigated. Alkyl bromides such as *n*-heptyl (4e), *n*-hexyl (4f), *n*-butyl (4g), *n*-propyl (4h) and ethyl (4r) delivered the desired products in moderate to good yields. Interestingly, long-chain alkyl bromides such as *n*-decyl (4i) and *n*-dodecyl (4j) afforded the corresponding products in appreciable yields; such hydrophobic substituents are characteristic pharmacophores in antibacterial coumarins.^18^ Isoamyl with a branched alkyl group was also readily accepted in this process and product 4k was obtained in 66% yield. The reaction was well-tolerated towards functional groups such as phenoxy, bromo, and cyano groups in alkyl bromides to deliver the desired products 4l–4n and 4u in moderate yields, highlighting its versatility and applicability in diverse chemical transformations.

**Table 2:** Substrate scope towards Pd/NBE-catalyzed alkylation/vinylation of 4-bromocoumarins^a^

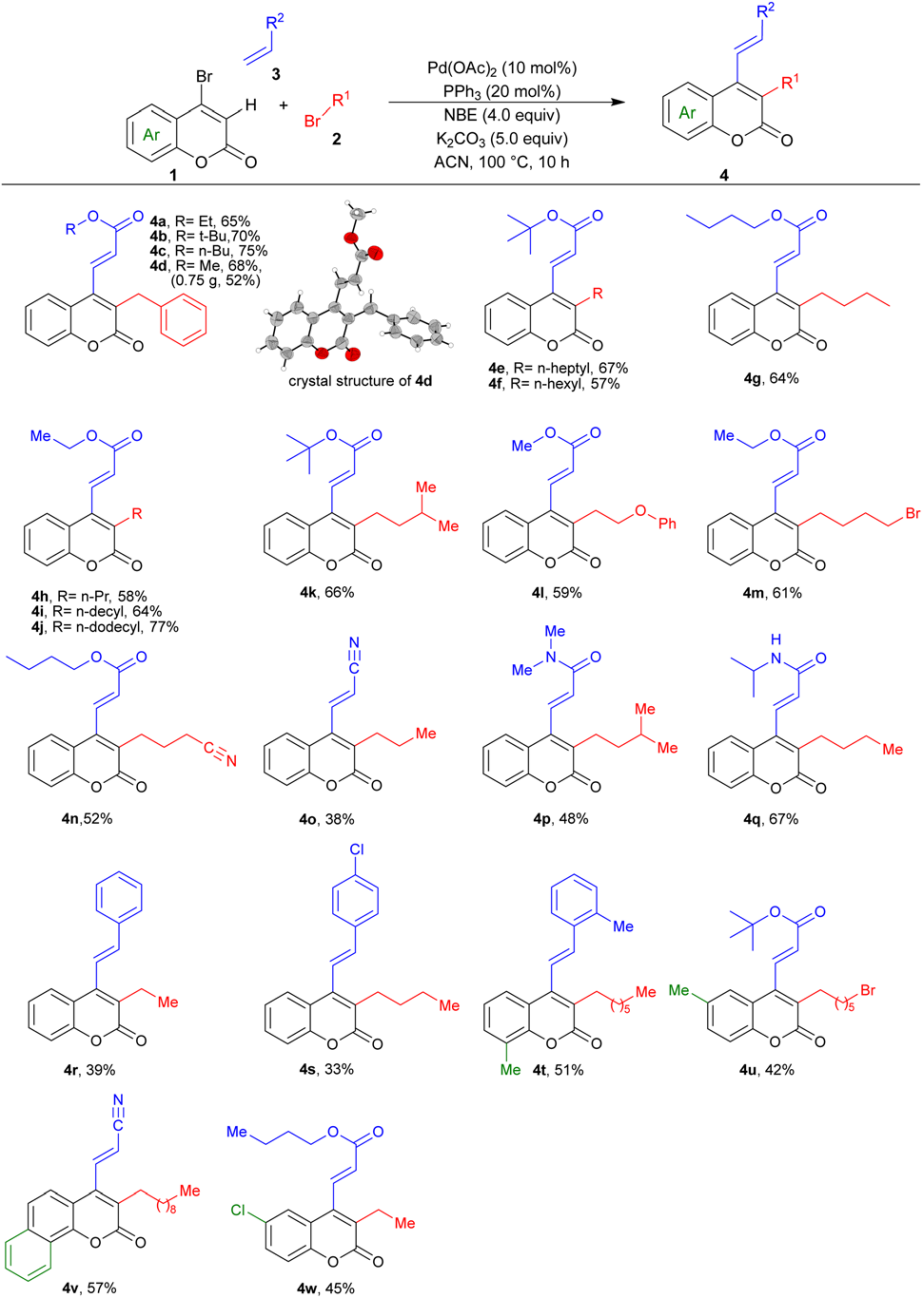

a Reaction conditions: 1 (0.1 mmol), 2 (0.5 mmol), 3 (0.2 mmol), Pd(OAc)_2_ (10 mol%), PPh_3_ (20 mol%), norbornene (0.4 mmol), K_2_CO_3_ (0.5 mmol), ACN (1.0 mL), at 100 °C for 10 h.

Subsequently, the investigation was expanded to explore the reactivity of olefins with different electronic properties. Besides acrylates, a diverse selection of olefins, including acrylonitrile, *N*,*N*-dimethyl acrylamide and *N*-isopropyl acrylamide demonstrated favorable results as terminating agents to afford relevant products 4o–4q. Notably, styrene and its derivatives such as *para*-chloro and *ortho*-methyl substituted ones, presented good efficacy as coupling partners in the ultimate step to produce products 4r–4t, respectively.

A significant trend was also observed in the context of the electronic effects of substituted 4-bromocoumarins reacting with different alkyl bromides and alkenes. The inclusion of methyl groups at different positions of bromocoumarin resulted in a successful formation of the desired products 4tand 4u in satisfactory yields. A particularly delightful outcome was observed when utilizing benzo-fused coumarin, as it led to 57% yield of the product 4v. Furthermore, the incorporation of chlorine in coumarin structure also facilitated the successful generation of the corresponding product 4w, presenting potential avenues for subsequent derivatization. The robustness of the method was further demonstrated by a scale-up of the reaction, affording compound 4d in 52% yield.

A plausible mechanism for the functionalization of 4-bromocoumarin as a partial aromatic scaffold is outlined in Scheme 2, based on literature precedents^8–10^ and its likely resemblance to the mechanism of aromatic systems. The reaction starts with bromocoumarin, which undergoes oxidative addition to generate the vinyl palladium species I. The subsequent carbopalladation with norbornene, followed by vinylic C–H palladation in the presence of a base, leads to the formation of the key alkenyl–norbornyl–palladacycle complex (ANP) III. The second oxidative addition with an alkyl bromide yields Pd(iv) species (intermediate IV). Next, the reductive elimination of intermediate IV, installs the alkyl group at C3 of the coumarin. Following this, the β-carbon elimination reaction leads to the removal of norbornene, yielding intermediate VI, a palladated coumarin at the C4 position. The final step involves a Heck reaction, which yields the alkenylated coumarin at C4 and generates Pd(0) species. Thus the process involves a sequential installation of alkyl at the vinylic position and alkenyl groups at the C4. This is one of the rare examples of the vinylogous Catellani reaction.

**Scheme 2:**
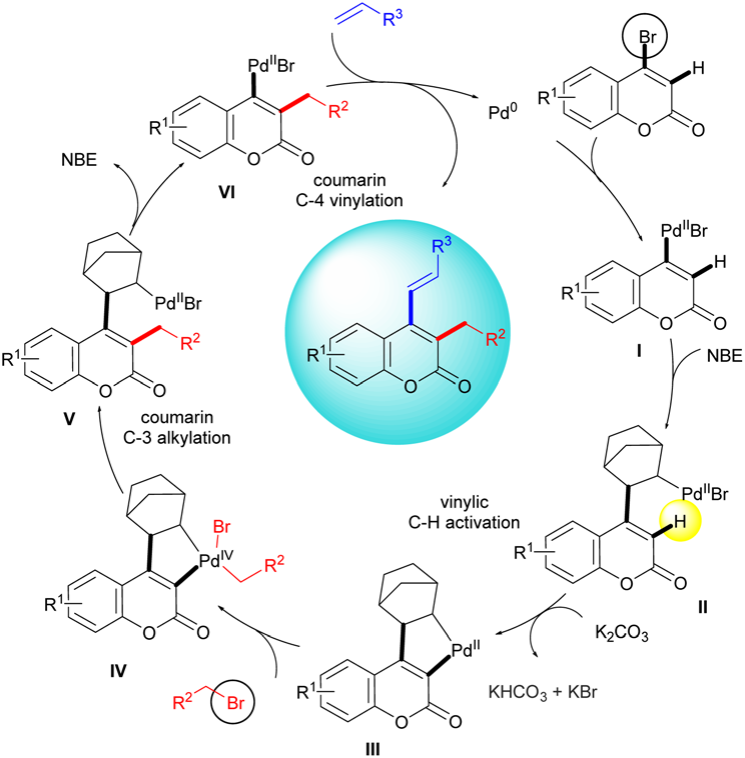
Proposed reaction mechanism.

In conclusion, this study establishes a new vinylic Catellani-type protocol that overcomes longstanding challenges associated with dual bromide systems. The use of 4-bromocoumarins as vinylogous electrophilic platforms enables efficient, regioselective difunctionalization with a variety of alkyl bromides, including electronically and sterically demanding substrates. The mild and operationally simple conditions eliminate the need for tailored ligands or modified norbornenes, expanding the synthetic utility of this transformation. Given the wide bioactivity and material relevance of the coumarin core, the resulting 3,4-disubstituted coumarins represent valuable building blocks for both medicinal chemistry and photonic materials, underscoring the broader applicability of this platform in complex molecule construction.

## Conflicts of interest

There are no conflicts to declare.

## Supplementary Material

RA-015-D5RA05007C-s001

RA-015-D5RA05007C-s002

## Data Availability

CCDC 2430471 contains the supplementary crystallographic data for this paper.^19^ The data underlying this study are available in the published article and its SI. Supplementary information: experimental procedures, compound characterization data, X-ray crystallography data and ^1^H/^13^C NMR spectra (PDF). See DOI: https://doi.org/10.1039/d5ra05007c.
