# Supplementary material for: Regioselective C3–C4 difunctionalization of 4-bromocoumarins via Pd/norbornene catalysis
Source: RSC Adv. 2025 Sep 22;15(41):34434–8. doi: 10.1039/d5ra05007c (PMC12451435; doi:10.1039/d5ra05007c)
Supplement: RA-015-D5RA05007C-s001 [file RA-015-D5RA05007C-s001.pdf]

Supplementary Information (SI) for RSC Advances.
This journal is © The Royal Society of Chemistry 2025

Supporting Information

Regioselective C3–C4 Difunctionalization of 4-Bromocoumarins via Pd/Norbornene Catalysis

Aynaz Feyzi and Farnaz Jafarpour*

School of Chemistry, College of Science, University of Tehran, 14155-6455 Tehran, Iran

jafarpour@ut.ac.ir

Table of Contents

1. General Considerations S2
   1.1 Materials and Methods S2
   1.2 Analytical Techniques S2
2. Experimental procedure S2
   2.1 General procedure for functionalization of 4-bromocoumarin S2
   2.2 Scale-Up Procedure for the Synthesis of **4d** S3
3. Optimization of reaction conditions S3
   3.1 Screening of Pd Catalysts and Ligand S3
   3.2 Screening of Bases S4
   3.3 Screening of Solvents S5
   3.4 Base and Norbornene Stoichiometry Variations S5
   3.5 Screening of Reaction Temperature and Time S6
4. Experimental characterization data S7
5. Single Crystal X-Ray S15
   5.1 Single Crystal X-Ray of **4d** S15
6. Copies of ^{1}H and ^{13}C NMR Spectra S17
7. Reference(s) S40

1. General Considerations

1.1 Materials and Methods

Synthesis of 4-bromocoumarin derivatives were performed according to literature procedures.[1] Other reagents and metal catalysts [$Pd(OAc)_2$, $PdCl_2$, $Pd(dba)_2$, $Pd(acac)_2$, $PdCl_2(NCCH_3)_2$] were commercially available and used as received. The reactions were carried out in oil bath using microwave vials (10 mL). Reaction progress was monitored using thin layer chromatography (TLC) and visualized with UV light (λ = 254 nm).

1.2 Analytical Techniques

^{1}H and ^{13}C NMR spectra were recorded at room temperature on 400, 500 and 100, 125 MHz spectrometers respectively, using $CDCl_3$ as the NMR solvent. ^{1}H NMR spectra are referenced to tetramethylsilane (0.00 ppm) and ^{13}C NMR spectra are referenced from the solvent central peak (77.16 for $CDCl_3$). Chemical shifts are given in ppm (δ). Coupling constants were reported in Hertz (Hz). Multiplicity for ^{1}H NMR spectra were reported using the following abbreviations: s = singlet, d = doublet, t = triplet, q = quartet, quin = quintet, sext = sextet, dd = doublet of doublets, td = triplet of doublets, ddd = doublet of doublet of doublets and m = multiplet. Melting points were measured on an Electrothermal 9100 apparatus. Mass spectra were recorded on an Agilent Technologies (HP) 5973 mass spectrometer operating at an ionization potential of 70 eV. Elemental analyses (CHN) were recorded on a Thermo Finnigan Flash EA 1112 elemental analyzer.

2. Experimental procedure

2.1 General procedure for functionalization of 4-bromocoumarin

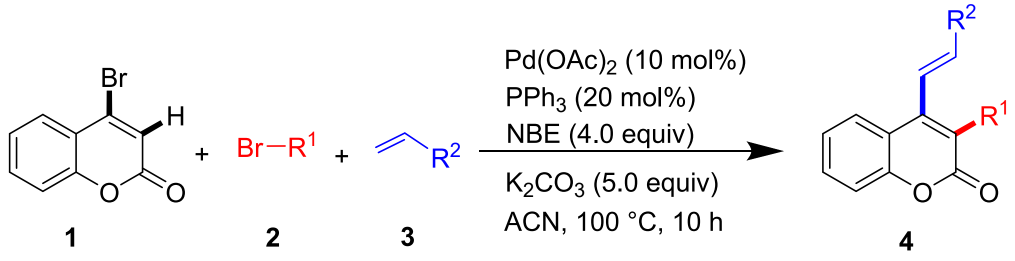

A vial equipped with a stir bar was charged with 4-bromocoumarin **1** (0.1 mmol, 1.0 equiv), alkyl bromide **2** (0.5 mmol, 5.0 equiv), olefin **3** (0.2 mmol, 2.0 equiv), $Pd(OAc)_2$ (0.0022 g, 0.01 mmol, 10 mol%), PPh_3 (0.005 g, 0.02 mmol, 20 mol%), norbornene (0.037 g, 0.4 mmol, 4.0 equiv), K_2CO_3 (0.07 g, 0.5 mmol, 5.0 equiv) and dry ACN (1.0 mL) was added and the vial was capped. The resulting mixture was heated in an oil bath at 100 °C for 10 h, cooled then filtered through a short plug of silica. Removal of the solvent gave a crude mixture which was purified by column chromatography (hexane/EtOAc gradient) to give the desired product **4**.

2.2 Scale-Up Procedure for the Synthesis of 4d

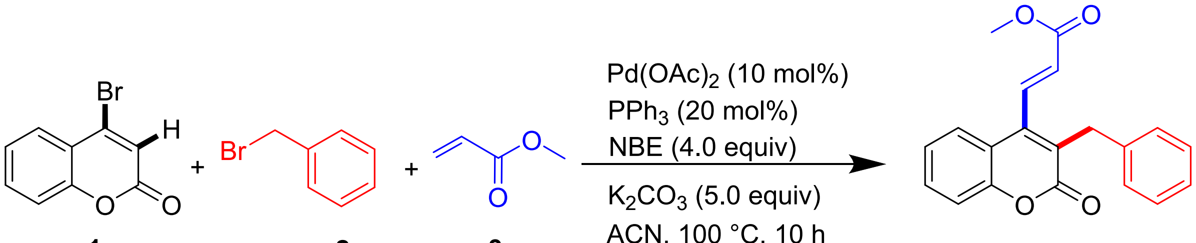

A vial equipped with a stir bar was charged with 4-bromocoumarin **1** (4.5 mmol, 1.0 g), alkyl bromide **2** (5.0 equiv , 3.80 g), olefin **3** (2.0 equiv, 0.77 g), $Pd(OAc)_2$ (10 mol%, 0.1 g), PPh_3 (20 mol%, 0.23 g), norbornene (4.0 equiv, 1.7 g), K_2CO_3 (5.0 equiv, 3.0 g) and dry ACN (45.0 mL) was added and the vial was capped. The resulting mixture was heated in an oil bath at 100 °C for 10 h, cooled then filtered through a short plug of silica. Removal of the solvent gave a crude mixture which was purified by column chromatography (hexane/EtOAc gradient) to give the desired product **4d** in 52% yield.

3. Optimization of reaction conditions

3.1 Screening of Pd Catalysts and Ligand

Table S1. Screening of Pd Catalysts and Ligand[a]

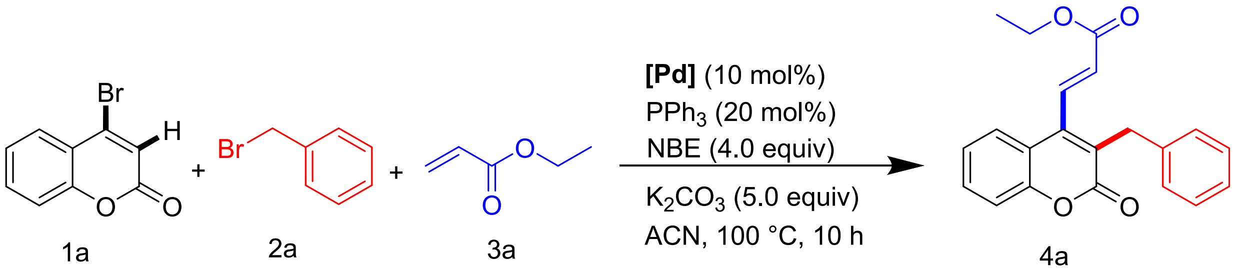

entry	variations	yield of 4a (%)[b]
1	$Pd(OAc)_2/PPh_3$	65
2	$Pd(OAc)_2$/No Ligand[c]	34
3	$PdCl_2$ instead of $Pd(OAc)_2$	43
4	$Pd(dba)_2$ instead of $Pd(OAc)_2$	50
5	$Pd(acac)_2$ instead of $Pd(OAc)_2$	40
6	$PdCl_2(NCCH_3)_2$ instead of $Pd(OAc)_2$	44

[a]All reactions were run under the following conditions: **1a** (0.1 mmol), **2a** (0.5 mmol), **3a** (0.2 mmol), Pd (0.01 mmol), PPh_3 (0.02 mmol), norbornene (0.4 mmol), K_2CO_3 (0.5 mmol), ACN (1.0 mL), at 100 °C for 10 h. [b]Isolated yields. [c]The reaction was carried out without any ligand.

3.2 Screening of bases

Table S2. Screening of bases[a]

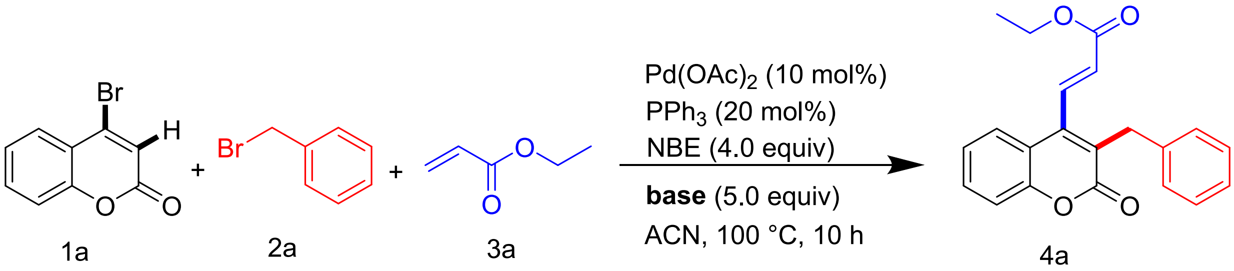

entry	Change from standard conditions	yield of 4a (%)[b]
1	none	65
2	NaOAc instead of K_2CO_3	33
3	Cs_2CO_3 instead of K_2CO_3	45
4	$NaHCO_3$ instead of K_2CO_3	0
5	KOAc instead of K_2CO_3	37

[a]All reactions were run under the following conditions: **1a** (0.1 mmol), **2a** (0.5 mmol), **3a** (0.2 mmol), $Pd(OAc)_2$ (0.01 mmol), PPh_3 (0.02 mmol), norbornene (0.4 mmol), base (0.5 mmol), ACN (1.0 mL), at 100 °C for 10 h. [b]Isolated yields.

3.3 Screening of Solvents

Table S3. Screening of Solvents[a]

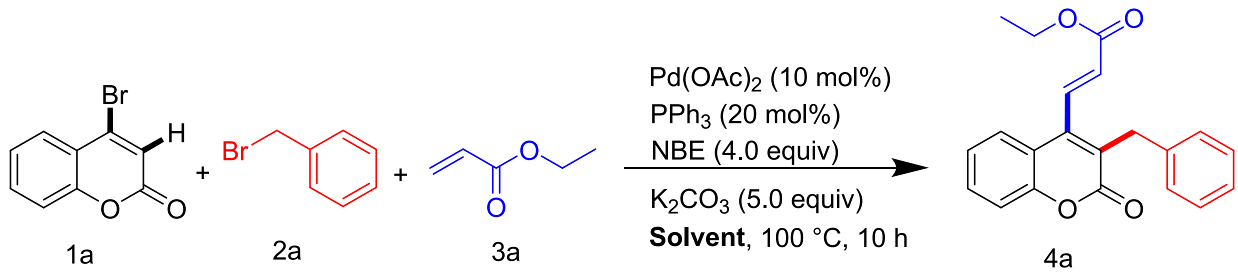

entry	Change from standard conditions	yield of 4a (%)[b]
1	none	65
2	1,4-dioxane instead of ACN	51
3	THF instead of ACN	45
4	toluene instead of ACN	40
5	DMSO instead of ACN	0
6	DMF instead of ACN	32
7	DME instead of ACN	0

[a]All reactions were run under the following conditions: **1a** (0.1 mmol), **2a** (0.5 mmol), **3a** (0.2 mmol), $Pd(OAc)_2$ (0.01 mmol), PPh_3 (0.02 mmol), norbornene (0.4 mmol), K_2CO_3 (0.5 mmol), solvent (1.0 mL), at 100 °C for 10 h. [b]Isolated yields.

3.4 Base and Norbornene Stoichiometry Variations

Table S4. Base and Norbornene Stoichiometry Variations[a]

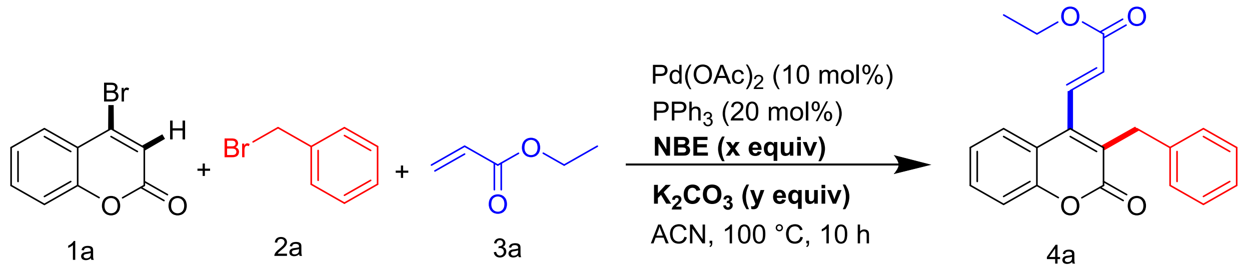

entry	Change from standard conditions	yield of 4a (%)[b]
1	none	65
2	NBE (3.0 equiv)	49
3	NBE (6.0 equiv)	61
4	K_2CO_3 (3.0 equiv)	40

[a]All reactions were run under the following conditions: **1a** (0.1 mmol), **2a** (0.5 mmol), **3a** (0.2 mmol), $Pd(OAc)_2$ (0.01 mmol), PPh_3 (0.02 mmol), norbornene (x mmol), K_2CO_3 (y mmol), ACN (1.0 mL), at 100 °C for 10 h. [b]Isolated yields.

3.5 Screening of Reaction Temperature and Time

Table S5. Screening of Reaction Temperature and Time[a]

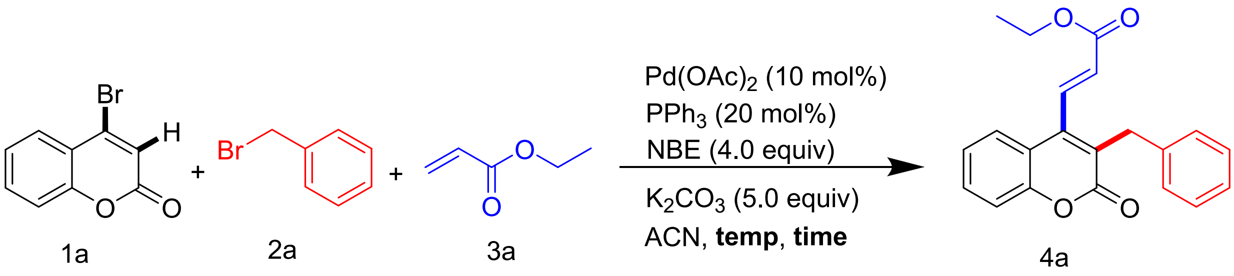

entry	Temperature (°C) / time (h)	yield of 4a (%)[b]
1	60 °C instead of 100 °C / 18 h	25
2	80 °C instead of 100 °C / 10 h	58
3	100 °C / 10 h	65
4	100 °C / 16 h instead of 10 h	63

[a]All reactions were run under the following conditions: **1a** (0.1 mmol), **2a** (0.5 mmol), **3a** (0.2 mmol), $Pd(OAc)_2$ (0.01 mmol), PPh_3 (0.02 mmol), norbornene (0.4 mmol), K_2CO_3 (0.5 mmol), ACN (1.0 mL). [b]Isolated yields.

4. Experimental characterization data

ethyl (E)-3-(3-benzyl-2-oxo-2H-chromen-4-yl)acrylate (4a)

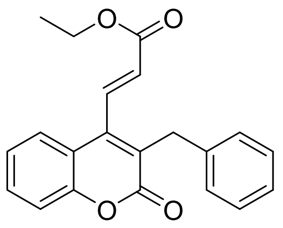

Light brown solid; yield 65% (22 mg); mp 84-86 °C; R_f= 0.08 on silica gel (Hexane/EtOAc 95:5). **^{1}H NMR** (500 MHz, $CDCl_3$) δ 7.77 (d, *J*=16.4 Hz, 1H), 7.54 (d, *J*=8.0 Hz, 1H), 7.51 (t, *J*=7.8 Hz, 1H), 7.34 (d, *J*=8.3 Hz, 1H), 7.31-7.23 (m, 5H), 7.22-7.16 (m, 1H), 6.22 (d, *J*=16.5 Hz, 1H), 4.33 (q, *J*=7.1 Hz, 2H), 4.02 (s, 2H), 1.37 (t, *J*= 7.1 Hz, 3H); **^{13}C NMR** (125 MHz, $CDCl_3$) δ 165.1, 161.2, 152.8, 145.2, 138.4, 137.4, 131.5, 128.7, 128.5, 126.6, 125.9, 125.9, 124.4, 118.5, 117.1, 61.4, 33.8, 14.3; **EI-MS** *m/z* (%): 334 (M•+, 42), 289 (12), 261 (100), 215 (54), 183 (41), 155 (29), 115 (22), 91 (35). Anal. Calcd for $C_{21}H_{18}O_4$: C, 75.43; H, 5.43; O; Found: C, 75.68; H, 5.45.

tert-butyl (E)-3-(3-benzyl-2-oxo-2H-chromen-4-yl)acrylate (4b)

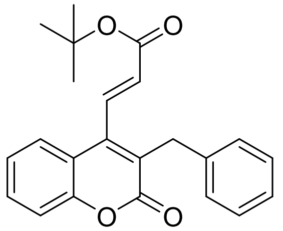

Beige solid; yield 70% (25 mg); mp 106-108 °C; R_f= 0.13 on silica gel (Hexane/EtOAc 95:5). **^{1}H NMR** (500 MHz, $CDCl_3$): δ 7.66 (d, *J* = 16.5 Hz, 1H), 7.56 (d, *J* = 8.0 Hz, 1H), 7.53– 7.48 (m, 1H), 7.35 (d, *J* = 8.4 Hz, 1H), 7.28 – 7.24 (m, 5H), 7.22-7.17 (m, 1H), 6.12 (d, *J* = 16.5 Hz, 1H), 4.01 (s, 2H), 1.56 (s, 9H); **^{13}C NMR** (125 Hz, $CDCl_3$) δ 164.4, 161.4, 152.8, 145.6, 138.5, 136.3, 131.5, 130.4, 128.7, 128.7, 126.7, 126.0, 125.9, 124.4, 118.6, 117.1, 81.9, 33.9, 28.3; **EI-MS** *m/z* (%): 362 (M•+, 26), 306 (63), 289 (48), 261 (100), 231 (48), 202 (76), 183 (43), 155 (35), 91 (65), 57 (93), 41 (60). Anal. Calcd for $C_{23}H_{22}O_4$: C, 76.22; H, 6.12; Found: C, 76.37; H, 6.14.

butyl (E)-3-(3-benzyl-2-oxo-2H-chromen-4-yl)acrylate (4c)

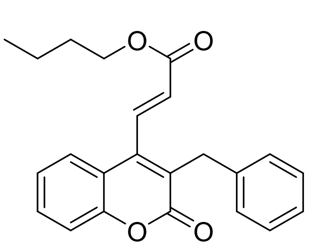

Light brown solid; yield 75% (27 mg); mp 70-72 °C; R_f= 0.11 on silica gel (Hexane/EtOAc 95:5). **^{1}H NMR** (500 MHz, $CDCl_3$): δ 7.77 (d, *J* = 16.3 Hz, 1H), 7.54 (d, *J* = 8.1 Hz, 1H), 7.50 (d, *J* = 8.2 Hz, 1H), 7.35 (d, *J* = 8.2 Hz, 1H), 7.30-7.23 (m, 5H), 7.22 – 7.16 (m, 1H), 6.22 (d, *J* = 16.5 Hz, 1H), 4.27 (t, *J* = 6.7 Hz, 2H), 4.02 (s, 2H), 1.72 (quin, *J* = 6.9 Hz, 2H), 1.45 (sext, *J* = 7.4 Hz, 2H), 0.99 (t, *J* = 7.4 Hz, 3H); **^{13}C NMR** (125 Hz, $CDCl_3$) δ 165.1, 161.1, 152.7, 145.1, 138.3, 137.3, 131.4, 128.6, 128.5, 128.4, 126.5, 125.8, 125.8, 124.3, 118.4, 116.9, 65.1, 33.7, 30.6, 19.1, 13.7; **EI-MS** *m/z* (%): 362 (M•+, 16), 305 (18), 261 (100), 203 (57), 133 (25), 96 (47), 64 (75) . Anal. Calcd for $C_{23}H_{22}O_4$: C, 76.22; H, 6.12; Found: C, 75.92; H, 6.10.

methyl (E)-3-(3-benzyl-2-oxo-2H-chromen-4-yl)acrylate (4d)

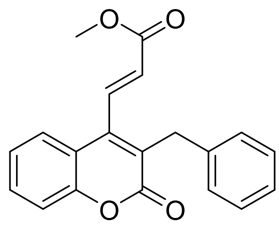

Beige solid; yield 68% (22 mg); mp 96-98 °C; R_f= 0.22 on silica gel (Hexane/EtOAc 95:5). **^{1}H NMR** (500 MHz, $CDCl_3$): δ 7.79 (d, *J* = 16.5 Hz, 1H), 7.58 – 7.48 (m, 2H), 7.35 (d, *J*= 8.2 Hz, 1H), 7.31 – 7.23 (m, 5H), 7.22-7.17 (m, 1H), 6.23 (d, *J* = 16.5 Hz, 1H), 4.02 (s, 2H), 3.87 (s, 3H). **^{13}C NMR** (125 MHz, $CDCl_3$): δ 165.5, 161.2, 152.7, 145.1, 138.3, 137.7, 131.5, 128.7, 128.4, 128.1, 126.6, 125.9, 125.8, 124.4, 118.4, 117.1, 52.3, 33.8; **EI-MS** *m/z* (%): 320 (M•+, 56), 289 (10), 261 (100), 215 (35), 183 (52), 155(36), 115(23), 91(35). Anal. Calcd for $C_{20}H_{16}O_4$: C, 74.99; H, 5.03; Found: C, 75.31; H, 5.05.

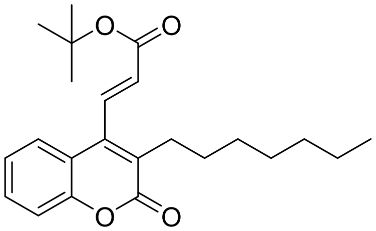

tert-butyl (E)-3-(3-heptyl-2-oxo-2H-chromen-4-yl)acrylate (4e)

Beige solid; yield 67% (25 mg); mp 55-57 °C; R_f= 0.28 on silica gel (Hexane/EtOAc 95:5). **^{1}H NMR** (500 MHz, $CDCl_3$): δ 7.64 (d, *J* = 16.5 Hz, 1H), 7.54 (d, *J* = 8.0 Hz, 1H), 7.49 (t, *J* = 7.8 Hz, 1H), 7.33 (d, *J* = 8.3 Hz, 1H), 7.26 (t, *J* = 7.6 Hz, 1H), 6.17 (d, *J* = 16.3 Hz, 1H), 2.61 (t, *J* = 7.9 Hz, 2H), 1.58 (s, 9H), 1.58 – 1.49 (m, 2H), 1.41 – 1.23 (m, 8H), 0.88 (t, *J* = 7.0 Hz, 3H); **^{13}C NMR** (125 MHz, $CDCl_3$) δ 164.5, 161.1, 152.5, 144.0, 136.4, 130.9, 129.8, 127.8, 125.7, 124.2, 118.6, 116.9, 81.7, 31.7, 29.5, 28.9, 28.7, 28.5, 28.1, 22.6, 14.1; **EI-MS** *m/z* (%): 370 (M•+,15), 314 (32), 270 (100), 250 (30), 219 (64), 115 (43) 93 (71), 65 (38). Anal. Calcd for $C_{23}H_{30}O_4$: C, 74.56; H, 8.16; Found: C, 74.24; H, 8.13.

tert-butyl (E)-3-(3-hexyl-2-oxo-2H-chromen-4-yl)acrylate (4f)

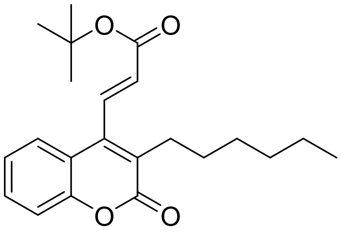

White solid; yield 57% (20 mg); mp 65-67 °C; R_f= 0.25 on silica gel (Hexane/EtOAc 95:5). **^{1}H NMR** (500 MHz, $CDCl_3$): δ 7.62 (d, *J* = 16.3 Hz, 1H), 7.53 (d, *J* = 8.0 Hz, 1H), 7.50 – 7.44 (m, 1H), 7.31 (d, *J* = 8.4 Hz, 1H), 7.27 – 7.22 (m, 1H), 6.16 (d, *J* = 16.3 Hz, 1H), 2.60 (t, *J*= 8.0 Hz, 2H), 1.58 – 1.50 (m, 11H), 1.41 – 1.33 (m, 2H), 1.33 – 1.23 (m, 4H), 0.87 (t, *J*= 7.3 Hz, 3H); **^{13}C NMR** (125 MHz, $CDCl_3$) δ 164.6, 161.3, 152.6, 144.1, 136.5, 131.0, 130.0, 127.9, 125.8, 124.3, 118.8, 117.0, 81.8, 31.5, 29.3, 28.7, 28.6, 28.2, 22.6, 14.1; **EI-MS** *m/z* (%): 356 (M•+,12), 283 (43), 271 (10), 255 (100), 197 (52), 141 (45), 77 (33), 57 (26). Anal. Calcd for $C_{22}H_{28}O_4$: C, 74.13; H, 7.92; Found: C, 74.11; H, 7.91.

butyl (E)-3-(3-butyl-2-oxo-2H-chromen-4-yl)acrylate (4g)

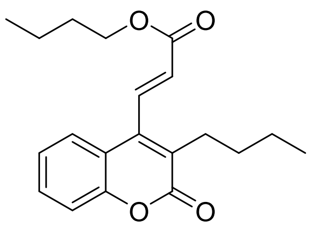

Yellow oil; yield 64% (21 mg); R_f= 0.21 on silica gel (Hexane/EtOAc 95:5). **^{1}H NMR** (500 MHz, $CDCl_3$): δ 7.67 (d, *J* = 16.4 Hz, 1H), 7.46 (d, *J* = 8.0 Hz, 1H), 7.42 (t, *J* = 7.8 Hz, 1H), 7.25 (d, *J* = 8.3 Hz, 1H), 7.19 (t, *J* = 7.7 Hz, 1H), 6.20 (d, *J* = 16.4 Hz, 1H), 4.22 (t, *J* = 6.8 Hz, 2H), 2.5 (t, *J*= 8.0 Hz, 2H), 1.67 (quin, *J* = 6.7 Hz, 2H), 1.523– 1.44 (m, 2H), 1.43-1.27 (m, 4H), 0.92 (t, *J* = 7.4 Hz, 3H), 0.87 (t, *J* = 7.3 Hz, 3H); **^{13}C NMR** (125 MHz, $CDCl_3$) δ 165.3, 161.0, 152.5, 143.7, 137.5, 130.9, 128.1, 127.8, 125.6, 124.2, 118.5, 116.9, 65.1, 30.8, 30.6, 28.3, 22.7, 19.2, 13.8, 13.7; **EI-MS** *m/z* (%): 328 (M•+, 12), 271 (5), 227 (100), 185 (41), 155 (5), 128 (12), 57 (12). Anal. Calcd for $C_{20}H_{24}O_4$: C, 73.15; H, 7.37; Found: C, 72.94; H, 7.36.

ethyl (E)-3-(2-oxo-3-propyl-2H-chromen-4-yl)acrylate (4h)

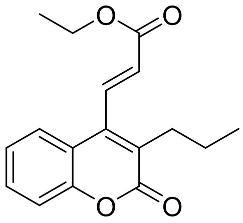

White solid; yield 58% (16.5 mg); mp 94-96 °C; R_f= 0.18 on silica gel (Hexane/EtOAc 95:5). **^{1}H NMR** (500 MHz, $CDCl_3$): δ 7.74 (d, *J* = 16.5 Hz, 1H), 7.56 – 7.46 (m, 2H), 7.34 (d, *J* = 8.2 Hz, 1H), 7.26 (t, *J* = 8.2 Hz, 1H), 6.25 (d, *J* = 16.5 Hz, 1H), 4.34 (q, *J* = 7.1 Hz, 2H), 2.60 (t, *J* = 7.8 Hz, 2H), 1.60 (sext, *J* = 7.4 Hz, 2H), 1.39 (t, *J* = 7.1 Hz, 3H), 0.99 (t, *J* = 7.4 Hz, 3H); **^{13}C NMR** (125 MHz, $CDCl_3$): δ 165.4, 161.2, 152.6, 144.1, 137.7, 131.1, 128.2, 127.8, 125.7, 124.3, 118.7, 117.1, 61.4, 30.6, 22.2, 14.3, 14.2; **EI-MS** m/z (%): 286 (M•+, 41), 241 (23), 213 (100), 185 (76), 155 (30), 128 (50), 102 (15), 77 (14), 51 (7). Anal. Calcd for $C_{17}H_{18}O_4$: C, 71.31; H, 6.34; Found: C, 72.21; H, 6.35.

ethyl (E)-3-(3-decyl-2-oxo-2H-chromen-4-yl)acrylate (4i)

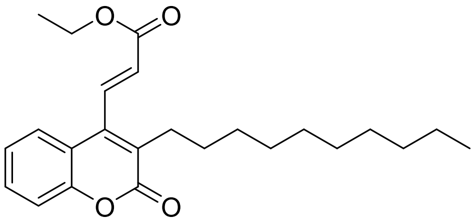

Colorless oil; yield 64% (24.5 mg); R_f= 0.22 on silica gel (Hexane/EtOAc 95:5). **^{1}H NMR** (500 MHz, $CDCl_3$): δ 7.72 (d, *J* = 16.4 Hz, 1H), 7.51 (d, *J* = 8.0 Hz, 1H), 7.50-7.46 (m, 1H), 7.32 (d, *J* = 8.1 Hz, 1H), 7.24 (t, *J* = 7.7 Hz, 1H), 6.24 (d, *J* = 16.4 Hz, 1H), 4.33 (q, *J* = 7.2 Hz, 2H), 2.60 (t, *J* = 8.0 Hz, 2H), 1.55 (quin, *J* = 7.3 Hz, 2H), 1.38 (t, *J* = 7.1 Hz, 2H), 1.32-1.15 (m, 15H), 0.86 (t, *J* = 6.9 Hz, 3H); **^{13}C NMR** (125 MHz, $CDCl_3$) δ 165.3, 161.1, 152.5, 143.8, 137.6, 131.0, 128.1, 128.0, 125.6, 124.3, 118.6, 117.0 61.3, 31.9, 29.7, 29.6, 29.5, 29.4, 29.3, 28.8, 28.6, 22.7, 14.3, 14.1; **EI-MS** *m/z* (%): 384 (M•+, 12), 339 (6), 311 (100), 285 (32), 185 (85), 128 (23), 105 (14), 77 (7), 43 (18). Anal. Calcd for $C_{24}H_{32}O_4$: C, 74.97; H, 8.39; Found: C, 74.72; H, 8.36.

ethyl (E)-3-(3-dodecyl-2-oxo-2H-chromen-4-yl)acrylate (4j)

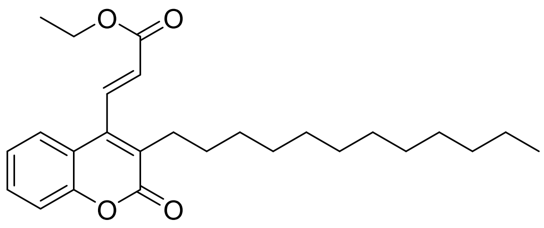

Colorless oil; yield 77% (32 mg); R_f= 0.21 on silica gel (Hexane/EtOAc 95:5). **^{1}H NMR** (500 MHz, $CDCl_3$): δ 7.69 (d, *J* = 16.4 Hz, 1H), 7.47 (d, *J* = 8.0 Hz, 1H), 7.43 (t, *J* = 7.8 Hz, 1H), 7.25 (d, *J* = 8.3 Hz, 1H), 7.20 (t, *J* = 7.7 Hz, 1H), 6.21 (d, *J* = 16.4 Hz, 1H), 4.30 (q, *J* = 7.1 Hz, 2H), 2.56 (t, *J* = 8.0 Hz, 2H), 1.51 (quin, *J* = 7.4 Hz, 2H), 1.34 (t, *J* = 7.2 Hz, 4H), 1.27-1.15 (m, 17H), 0.83 (t, *J* = 6.9 Hz, 3H); **^{13}C NMR** (125 MHz, $CDCl_3$) δ 165.0, 160.8, 152.4, 143.5, 137.4, 130.8, 128.0, 127.9, 127.8, 125.5, 124.1, 118.4, 116.8, 61.1, 31.8, 29.5, 29.5, 29.4, 29.2, 29.2, 28.6, 28.5, 22.6, 14.1, 14.0; **EI-MS** *m/z* (%): 412 (M•+, 14), 339 (100), 285 (33), 185 (5.9), 128 (14), 91 (70), 43 (15). Anal. Calcd for $C_{26}H_{36}O_4$: C, 75.69; H, 8.80; Found: C, 75.81; H, 8.81.

tert-butyl (E)-3-(3-isopentyl-2-oxo-2H-chromen-4-yl)acrylate (4k)

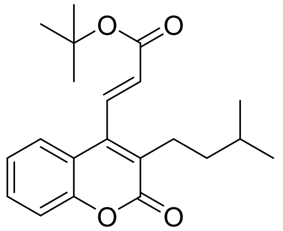

White solid; yield 66% (22.5 mg); mp 73-75 °C; R_f= 0.25 on silica gel (Hexane/EtOAc 95:5). **^{1}H NMR** (500 MHz, $CDCl_3$): δ 7.61 (d, *J* = 16.3 Hz, 1H), 7.53 (d, *J* = 8.0 Hz, 1H), 7.50 – 7.44 (m, 1H), 7.32 (d, *J* = 8.4 Hz, 1H), 7.27 – 7.22 (m, 1H), 6.17 (d, *J* = 16.3 Hz, 1H), 2.60 (t, *J*= 8.1 Hz, 2H), 1.65-1.59 (m, 1H), 1.55 (s, 9H), 1.46 – 1.38 (m, 2H), 0.93 (d, *J* = 6.6 Hz, 6H); **^{13}C NMR** (125 MHz, $CDCl_3$) δ 164.5, 161.3, 152.6, 144.0, 136.4, 131.0, 130.0, 128.1, 125.8, 124.3, 118.8, 117.0, 81.8, 37.8, 28.4, 28.2, 26.6, 22.4; **EI-MS** *m/z* (%): 342 (M•+, 5), 286 (26), 241 (100), 128 (37), 69 (26), 41 (86). Anal. Calcd for $C_{21}H_{26}O_4$: C, 73.66; H, 7.65; Found: C, 73.31; H, 7.62.

methyl (E)-3-(2-oxo-3-(2-phenoxyethyl)-2H-chromen-4-yl)acrylate (4l)

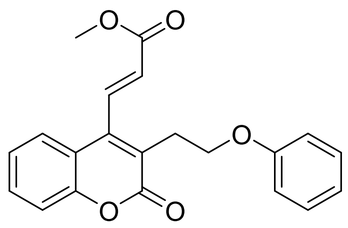

White solid; yield 59% (20.6 mg); mp 138-140 °C; R_f= 0.08 on silica gel (Hexane/EtOAc 90:10). **^{1}H NMR** (500 MHz, $CDCl_3$): δ 7.84 (d, *J* = 16.5 Hz, 1H), 7.59-7.50 (m, 2H), 7.35 (d, *J* = 8.4 Hz, 1H), 7.31 – 7.22 (m, 3H), 6.96 – 6.87 (m, 3H), 6.50 (d, *J* = 16.3 Hz, 1H), 4.28 (t, *J* = 6.0 Hz, 2H), 3.89 (s, 3H), 3.13 (t, *J* = 6.0 Hz, 2H); **^{13}C NMR** (125 MHz, $CDCl_3$) δ 165.7, 161.1, 158.5, 152.6, 146.5, 137.8, 131.5, 129.5, 128.5, 125.9, 124.4, 123.4, 120.9, 118.5, 117.0, 114.3, 65.4, 52.2, 29.0; **EI-MS** *m/z* (%): 350 (M•+, 5), 257 (100), 197 (73), 169 (18), 153 (33), 115 (20), 77 (50), 59 (13). Anal. Calcd for $C_{21}H_{18}O_5$: C, 71.99; H, 5.18; Found: C, 71.87; H, 5.14.

ethyl (E)-3-(3-(4-bromobutyl)-2-oxo-2H-chromen-4-yl)acrylate (4m)

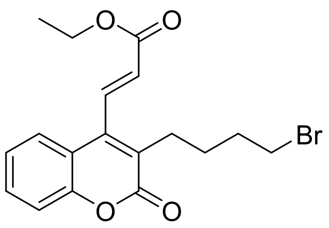

Yellow oil; yield 61% (23 mg); R_f= 0.08 on silica gel (Hexane/EtOAc 90:10). **^{1}H NMR** (500 MHz, $CDCl_3$): δ 7.67 (d, *J* = 16.5 Hz, 1H), 7.50 – 7.42 (m, 2H), 7.27 (d, *J* = 8.0 Hz, 1H), 7.21 (t, *J* = 7.6 Hz, 1H), 6.21 (d, *J* = 16.5 Hz, 1H), 4.28 (q, *J* = 7.1 Hz, 2H), 3.37 (t, *J* = 6.8 Hz, 2H), 2.62 – 2.55 (m, 2H), 1.89 (quin, *J* = 6.9 Hz, 2H), 1.66 (quin, *J* = 7.5 Hz, 2H), 1.33 (t, *J* = 7.2 Hz, 3H); **^{13}C NMR** (125 MHz, $CDCl_3$) δ 165.1, 160.9, 152.5, 144.4, 137.1, 131.2, 128.4, 126.8, 125.7, 124.3, 118.4, 117.0, 61.3, 33.1, 32.3, 27.5, 27.1, 14.2; **EI-MS** *m/z* (%): 379 (M•+, 6), 299 (100), 225 (16), 185 (36), 155 (13), 128 (19), 72 (5), 55 (6). Anal. Calcd for $C_{18}H_{19}BrO_4$: C, 57.01; H, 5.05; Found: C, 57.25; H, 5.07.

butyl (E)-3-(3-(3-cyanopropyl)-2-oxo-2H-chromen-4-yl)acrylate (4n)

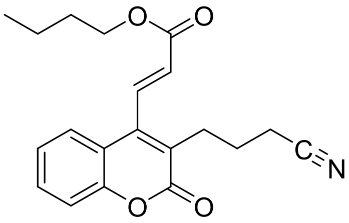

Colorless oil; yield 52% (17.6 mg); R_f= 0.17 on silica gel (Hexane/EtOAc 85:15). **^{1}H NMR** (400 MHz, $CDCl_3$): δ 7.74 (d, *J* = 16.4 Hz, 1H), 7.58-7.48 (m, 2H), 7.36 (d, *J* = 4.5 Hz, 1H), 7.32 – 7.25 (m, 1H), 6.26 (d, *J* = 16.4 Hz, 1H), 4.28 (t, *J* = 6.7 Hz, 2H), 2.81 – 2.72 (m, 2H), 2.43 (t, *J* = 7.2 Hz, 2H), 1.95 (quin, *J* = 7.3 Hz, 2H), 1.78 – 1.67 (m, 2H), 1.45 (sext, *J* = 7.4 Hz, 2H), 0.97 (t, *J* = 7.4 Hz, 3H); **^{13}C NMR** (100 MHz, $CDCl_3$): δ 165.1, 161.1, 152.7, 145.7, 136.8, 131.8, 129.0, 126.0, 125.1, 124.7, 119.2, 118.4, 117.2, 65.5, 30.7, 27.8, 24.5, 19.3, 17.3, 13.9; **EI-MS** *m/z* (%): 339 (M•+, 26), 299 (9), 265 (11), 238 (100), 197 (27), 169 (12), 128 (9), 41 (9). Anal. Calcd for $C_{20}H_{21}NO_4$: C, 70.78; H, 6.24; N, 4.13; Found: C, 70.91; H, 6.26; N, 4.15.

(E)-3-(2-oxo-3-propyl-2H-chromen-4-yl)acrylonitrile (4o)

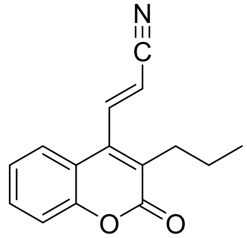

Colorless oil; yield 38% (9.0 mg); R_f= 0.13 on silica gel (Hexane/EtOAc 90:10). **^{1}H NMR** (500 MHz, $CDCl_3$): δ 7.54-7.51 (m, 1H), 7.50-7.45 (m, 1H), 7.45 (d, *J*= 7.9 Hz, 1H), 7.33 (d, *J* = 8.3 Hz, 1H), 7.31 – 7.25 (m, 1H), 5.80 (d, *J* = 16.9 Hz, 1H), 2.57 (t, *J*= 7.9 Hz, 2H), 1.58 (sext, *J* = 7.5 Hz, 2H), 0.99 (t, *J* = 7.4 Hz, 3H); **^{13}C NMR** (125 MHz, $CDCl_3$) δ 160.7, 152.6, 144.3, 142.4, 131.5, 128.4, 125.2, 124.6, 117.9, 117.3, 116.1, 107.3, 30.7, 22.3, 14.2; **EI-MS** *m/z* (%): 239 (M•+,60), 224 (100), 199 (75), 182 (35), 166 (39), 127 (56), 77 (23), 63 (15). Anal. Calcd for $C_{15}H_{13}NO_2$: C, 75.30; H, 5.48; N, 5.85; Found: C, 74.94; H, 5.45; N, 5.69.

(E)-3-(3-isopentyl-2-oxo-2H-chromen-4-yl)-N,N-dimethylacrylamide (4p)

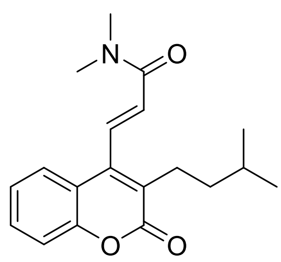

Yellow oil; yield 48% (15 mg); R_f= 0.11 on silica gel (Hexane/EtOAc 70:30). **^{1}H NMR** (400 MHz, $CDCl_3$): δ 7.72 (d, *J* = 15.9 Hz, 1H), 7.55 (d, *J* = 7.9 Hz, 1H), 7.52 – 7.45 (m, 1H), 7.35 – 7.28 (m, 1H), 7.24 (t, *J* = 8.3 Hz, 1H), 6.72 (d, *J* = 15.8 Hz, 1H), 3.16 (s, 3H), 3.13 (s, 3H), 2.68 – 2.57 (m, 2H), 1.67- 1.59 (m, 1H), 1.51 – 1.37 (m, 2H), 0.94 (d, *J* = 6.6 Hz, 6H); **^{13}C NMR** (100 Hz, $CDCl_3$) δ 165.0, 161.3, 152.5, 145.1, 135.5, 130.9, 127.7, 126.9, 125.8, 124.2, 119.1, 116.9, 37.9, 37.5, 36.1, 28.5, 26.8, 22.5; **EI-MS** *m/z* (%): 313 (M•+, 14), 269 (9), 241 (100), 158 (64), 72 (24), 57 (39). Anal. Calcd for $C_{19}H_{23}NO_3$: C, 72.82; H, 7.40; N, 4.47; Found: C, 72.52; H, 7.38; N, 4.44.

(E)-3-(3-butyl-2-oxo-2H-chromen-4-yl)-N-isopropylacrylamide (4q)

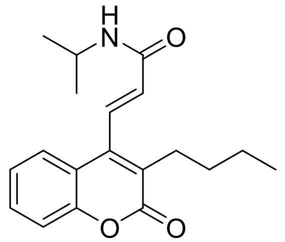

Beige solid; yield 67% (21 mg); mp 176-178 °C; R_f= 0.09 on silica gel (Hexane/EtOAc 80:20). **^{1}H NMR** (500 MHz, $CDCl_3$) δ 7.66 (d, *J* = 16.1 Hz, 1H), 7.52 (dd, *J* = 8.0, 1.6 Hz, 1H), 7.46-7.42 (m, 1H), 7.29 – 7.24 (m, 1H), 7.23 – 7.18 (m, 1H), 6.20 (d, *J* = 15.9 Hz, 1H), 6.01 (d, *J* = 7.7 Hz, 1H), 4.29-4.20 (m, 1H), 2.57 (t, *J* = 7.9 Hz, 2H), 1.53-1.44 (m, 2H), 1.35 (sext, *J* = 7.3 Hz, 2H), 1.26 (d, *J* = 6.6 Hz, 6H), 0.89 (t, *J* = 7.3 Hz, 3H); **^{13}C NMR** (125 MHz, $CDCl_3$) δ 163.2, 161.4, 152.6, 145.0, 133.9, 130.9, 130.7, 127.6, 125.9, 124.2, 119.0, 116.9, 42.1, 30.9, 28.3, 22.8, 13.9; **EI-MS** *m/z* (%): 313 (M•+, 12), 270 (6), 256 (100), 112 (28), 99 (11), 57 (30). Anal. Calcd for $C_{19}H_{23}NO_3$: C, 72.82; H, 7.40; N, 4.47; Found: C, 73.06; H, 7.42; N, 4.50.

(E)-3-ethyl-4-styryl-2H-chromen-2-one (4r)

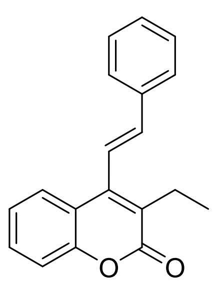

Yellow oil; yield 39% (10.7 mg); R_f= 0.20 on silica gel (Hexane/EtOAc 95:5). **^{1}H NMR** (400 MHz, $CDCl_3$): δ 7.74 (dd, *J* = 7.9, 1.6 Hz, 1H), 7.61 (d, *J* = 6.8 Hz, 2H), 7.53 – 7.45 (m, 3H), 7.43 (d, *J*= 7.3 Hz, 1H), 7.38 (dd, *J* = 8.2, 1.2 Hz, 1H), 7.29 (dd, *J* = 8.1, 1.1 Hz, 1H), 7.13 (d, *J*= 61.6 Hz, 1H), 6.92 (d, *J* = 16.6 Hz, 1H), 2.77 (q, *J* = 7.5 Hz, 2H), 1.25 (t, *J* = 7.5 Hz, 3H); **^{13}C NMR** (100 MHz, $CDCl_3$) δ 161.8, 152.7, 146.3, 138.0, 135.9, 130.7, 129.1, 129.1, 128.0, 127.0, 126.2, 124.1, 120.8, 119.8, 116.9, 22.1, 13.4; **EI-MS** *m/z* (%): 276 (M•+, 12), 261 (84), 199 (100), 167 (28), 77 (30). Anal. Calcd for $C_{19}H_{16}O_2$: C, 82.58; H, 5.84; Found: C, 82.70; H, 5.85.

(E)-3-butyl-4-(4-chlorostyryl)-2H-chromen-2-one (4s)

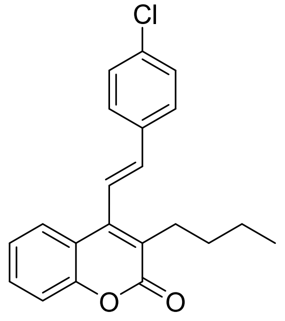

Yellow oil; yield 33% (11 mg); R_f= 0.21 on silica gel (Hexane/EtOAc 95:5). **^{1}H NMR** (500 MHz, $CDCl_3$): δ 7.68 (d, *J* = 8.0 Hz, 1H), 7.52-7.45 (m, 3H), 7.42 (d, *J* = 8.4 Hz, 2H), 7.36 (d, *J* = 8.2 Hz, 1H), 7.29 – 7.23 (m, 1H), 7.07 (d, *J* = 16.6 Hz, 1H), 6.83 (d, *J* = 16.8 Hz, 1H), 2.70 (t, *J*=7.7 Hz, 2H), 1.67 – 1.55 (m, 2H), 1.42 (sext, *J* = 7.3 Hz, 2H), 0.93 (t, *J* = 7.4 Hz, 3H); **^{13}C NMR** (125 MHz, $CDCl_3$): δ 161.8, 152.8, 146.1, 136.7, 135.0, 134.5, 130.8, 129.3, 128.1, 127.1, 126.1, 124.1, 121.7, 119.7, 117.1, 31.0, 28.4, 23.0, 14.0; **EI-MS** *m/z* (%): 338 (M•+, 88), 311 (28), 295 (91), 261 (15), 231 (48), 213 (100), 185 (32), 125 (6), 101 (12), 77 (6). Anal. Calcd for $C_{21}H_{19}ClO_2$: C, 74.44; H, 5.65; Found: C, 74.79; H, 5.69.

(E)-3-heptyl-8-methyl-4-(2-methylstyryl)-2H-chromen-2-one (4t)

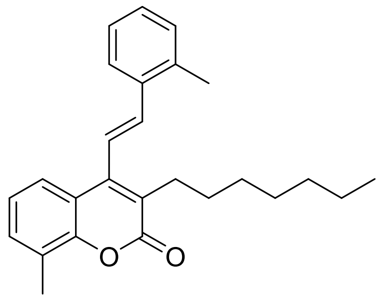

Yellow oil; yield 51% (19 mg); R_f= 0.38 on silica gel (Hexane/EtOAc 95:5). **^{1}H NMR** (400 MHz, $CDCl_3$): δ 7.69 – 7.65 (m, 1H), 7.58 – 7.54 (m, 1H), 7.34 (ddd, *J* = 7.3, 1.6, 0.9 Hz, 1H), 7.31 – 7.28 (m, 2H), 7.27 – 7.22 (m, 1H), 7.15 (t, *J* = 7.7 Hz, 1H), 7.09 (d, *J* = 16.6 Hz, 1H), 6.96 (d, *J* = 16.5 Hz, 1H), 2.71 (t, *J*= 7.9 Hz, 2H), 2.49 (s, 3H), 2.40 (s, 3H), 1.66 – 1.57 (m, 2H), 1.43 – 1.19 (m, 8H), 0.85 (t, *J*= 6.9 Hz, 3H); **^{13}C NMR** (100 MHz, $CDCl_3$) δ 162.0, 151.1, 147.0, 136.3, 135.8, 135.4, 132.0, 130.8, 128.9, 126.6, 126.6, 126.3, 125.8, 123.9, 123.5, 122.8, 119.7, 31.9, 29.9, 29.3, 29.1, 28.7, 22.8, 20.0, 15.9, 14.2; **EI-MS** *m/z* (%): 374 (M•+, 100), 331 (18), 303 (56), 275 (94), 227 (41), 199 (31), 105 (22), 43 (5). Anal. Calcd for $C_{26}H_{30}O_2$: C, 83.38; H, 8.07; Found: C, 83.03; H, 8.04.

tert-butyl (E)-3-(3-(6-bromohexyl)-6-methyl-2-oxo-2H-chromen-4-yl)acrylate (4u)

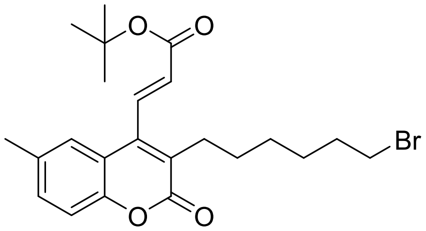

Light yellow oil; yield 42% (19 mg); R_f= 0.16 on silica gel (Hexane/EtOAc 95:5). **^{1}H NMR** (400 MHz, $CDCl_3$): δ 7.61 (d, *J* = 16.4 Hz, 1H), 7.34 – 7.26 (m, 2H), 7.20–7.16 (m, 1H), 6.14 (d, *J* = 16.4 Hz, 1H), 3.39 (t, *J* = 6.8 Hz, 2H), 2.64 – 2.56 (m, 2H), 2.39 (s, 3H), 1.84 (td, *J* = 12.5, 5.6 Hz, 2H), 1.58-1.53 (m, 11H), 1.45 –1.34 (m, 4H); **^{13}C NMR** (125 Hz, $CDCl_3$) δ 164.6, 161.4, 150.7, 144.3, 136.7, 134.0, 132.1, 129.8, 127.4, 125.6, 118.5, 116.8, 81.9, 33.8, 32.8, 28.8, 28.6, 28.5, 28.3, 27.9, 21.1; **EI-MS** *m/z* (%): 449 (M•+, 10), 369 (100), 299 (31), 254 (30), 149 (74), 91 (77), 57 (100). Anal. Calcd for $C_{23}H_{29}BrO_4$: C, 61.47; H, 6.50; Found: C, 61.57; H, 6.51.

(E)-3-(3-decyl-2-oxo-2H-benzo[h]chromen-4-yl)acrylonitrile (4v)

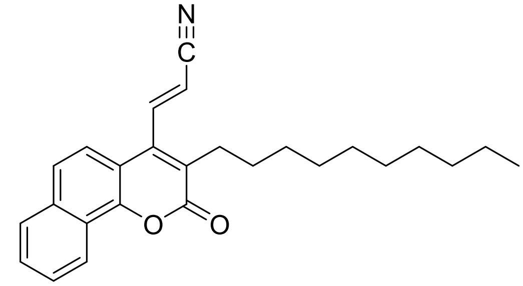

Orange solid; yield 57% (22 mg); mp 81-83 °C; R_f= 0.22 on silica gel (Hexane/EtOAc 90:10). **^{1}H NMR** (400 MHz, $CDCl_3$): δ 8.57-8.49 (m, 1H), 7.90 – 7.82 (m, 1H), 7.71 – 7.61 (m, 3H), 7.58 (dd, *J* = 17.1, 1.8 Hz, 1H), 7.43 (dd, *J* = 8.8, 1.6 Hz, 1H), 5.83 (dd, *J* = 17.0, 1.0 Hz, 1H), 2.64 (t, *J* = 7.2 Hz, 2H), 1.58 (quin, *J* = 7.3 Hz, 2H), 1.45 – 1.36 (m, 2H), 1.35-1.17 (m, 12H), 0.88 (t, *J*= 6.7 Hz, 3H); **^{13}C NMR** (100 MHz, $CDCl_3$) δ 160.9, 149.7, 144.8, 143.1, 134.4, 128.9, 128.1, 127.8, 127.6, 124.6, 123.2, 122.5, 120.8, 116.2, 113.1, 107.3, 32.0, 29.9, 29.7, 29.5, 29.4, 29.0, 28.9, 22.8, 14.3; **EI-MS** *m/z* (%): 387 (M•+, 77), 347 (42), 288 (100), 260 (81), 232 (72), 203 (52), 152 (22), 97 (24), 43 (42). Anal. Calcd for $C_{26}H_{29}NO_2$: C, 80.59; H, 7.54; N, 3.61; Found: C, 80.19; H, 7.51; N, 3.57.

butyl (E)-3-(6-chloro-3-ethyl-2-oxo-2H-chromen-4-yl)acrylate (4w)

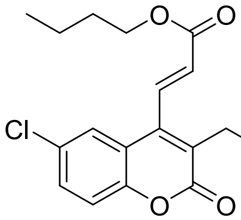

Colorless oil; yield 45% (15 mg); R_f= 0.15 on silica gel (Hexane/EtOAc 95:5). **^{1}H NMR** (400 MHz, $CDCl_3$): δ 7.67 (d, *J* = 16.4 Hz, 1H), 7.49 – 7.38 (m, 2H), 7.27 (d, *J* = 8.6 Hz, 1H), 6.25 (d, *J* = 16.4 Hz, 1H), 4.28 (t, *J* = 6.8 Hz, 2H), 2.70 – 2.57 (m, 2H), 1.73 (quin, *J* = 6.9 Hz, 2H), 1.45 (sext, *J* = 7.4 Hz, 2H), 1.17 (t, *J* = 7.5 Hz, 3H), 0.98 (t, *J* = 7.4 Hz, 3H); **^{13}C NMR** (100 Hz, $CDCl_3$) δ 165.3, 160.5, 150.9, 142.7, 136.7, 131.1, 130.2, 129.8, 128.6, 125.1, 119.8, 118.5, 65.5, 30.7, 22.3, 19.3, 13.9, 13.3; **EI-MS** *m/z* (%): 334 (M•+,62), 261 (76), 233 (100), 207 (48), 177 (35), 101(18), 57 (64). Anal. Calcd for $C_{18}H_{19}ClO_4$: C, 64.58; H, 5.72; Found: C, 64.34; H, 5.69.

5. Single Crystal X-Ray

5.1 Single Crystal X-Ray of 4d

Crystallization. The product was recrystallized using ethyl acetate and small amounts of hexane to afford analytically pure 4d.

Figure S1. X-ray crystal structure of **methyl (E)-3-(3-benzyl-2-oxo-2H-chromen-4-yl)acrylate**. Thermal ellipsoids are shown at the 50% probability level.

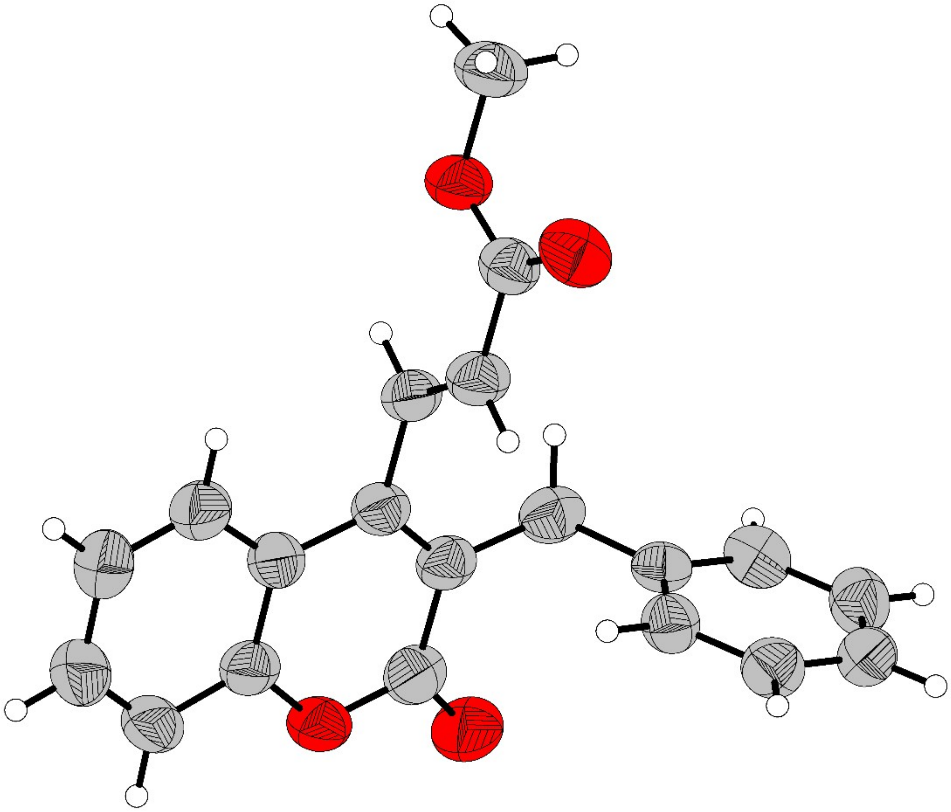

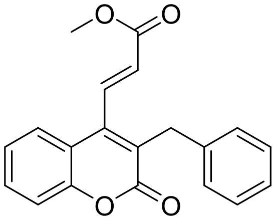

4d

Table S6. Crystal data

Datablock: 1

Bond precision:	C-C = 0.0049 A		Wavelength=0.71073
Cell:	a=5.6698(11)	b=10.893(2)	c=13.585(3)
	alpha=72.75(3)	beta=85.55(3)	gamma=86.34(3)
Temperature:	290 K		

	Calculated	Reported
Volume	798.1(3)	798.1(3)
Space group	P -1	P -1
Hall group	-P 1	-P 1
Moiety formula	C20 H16 O4	C20 H16 O4
Sum formula	C20 H16 O4	C20 H16 O4
Mr	320.33	320.33
Dx,g cm-3	1.333	1.333
Z	2	2
Mu (mm-1)	0.093	0.093
F000	336.0	336.0
F000’	336.18	
h,k,lmax	6,12,16	6,12,16
Nref	2806	2478
Tmin,Tmax	0.983,0.986	0.867,1.216
Tmin’	0.972	

Correction method= # Reported T Limits: Tmin=0.867 Tmax=1.216
AbsCorr = MULTI-SCAN

Data completeness= 0.883 Theta(max)= 25.000

R(reflections)= 0.0772(2057) wR2(reflections)= 0.2259(2478)

S = 1.142 Npar= 218

6. Copies of ^1H and ^{13}C NMR Spectra

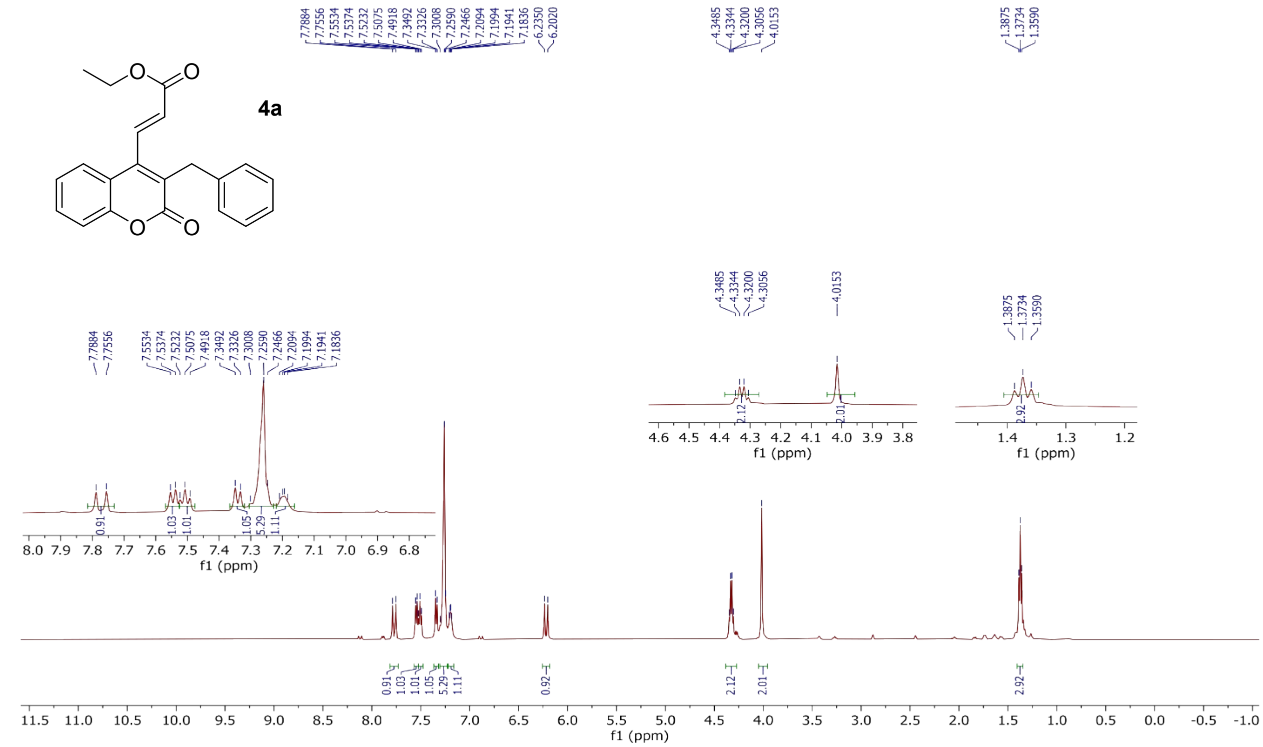

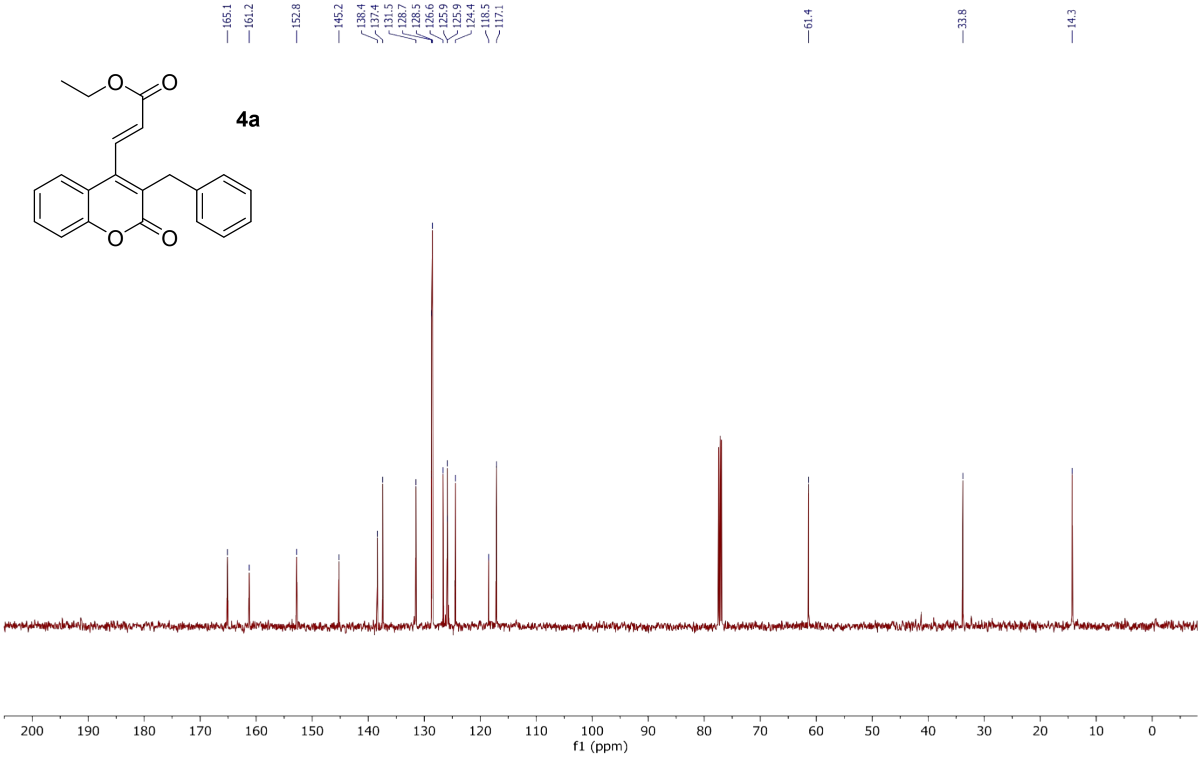

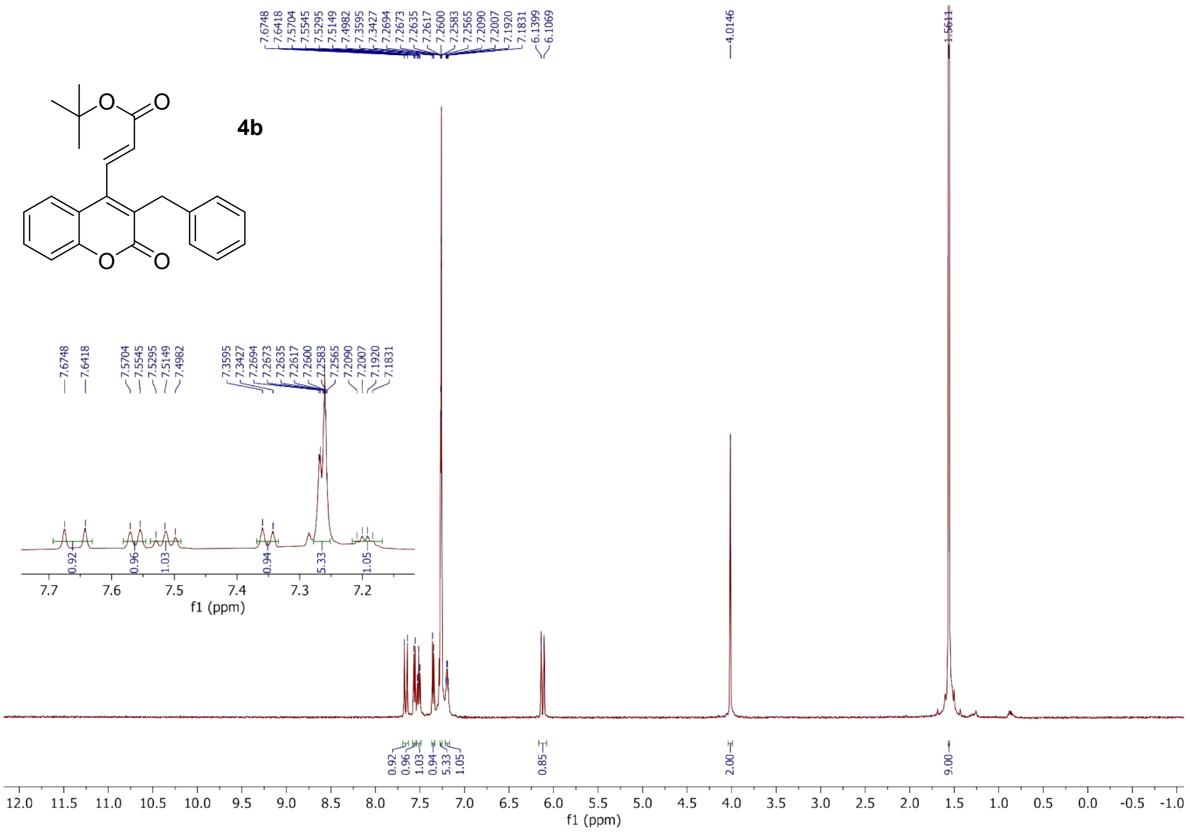

4b

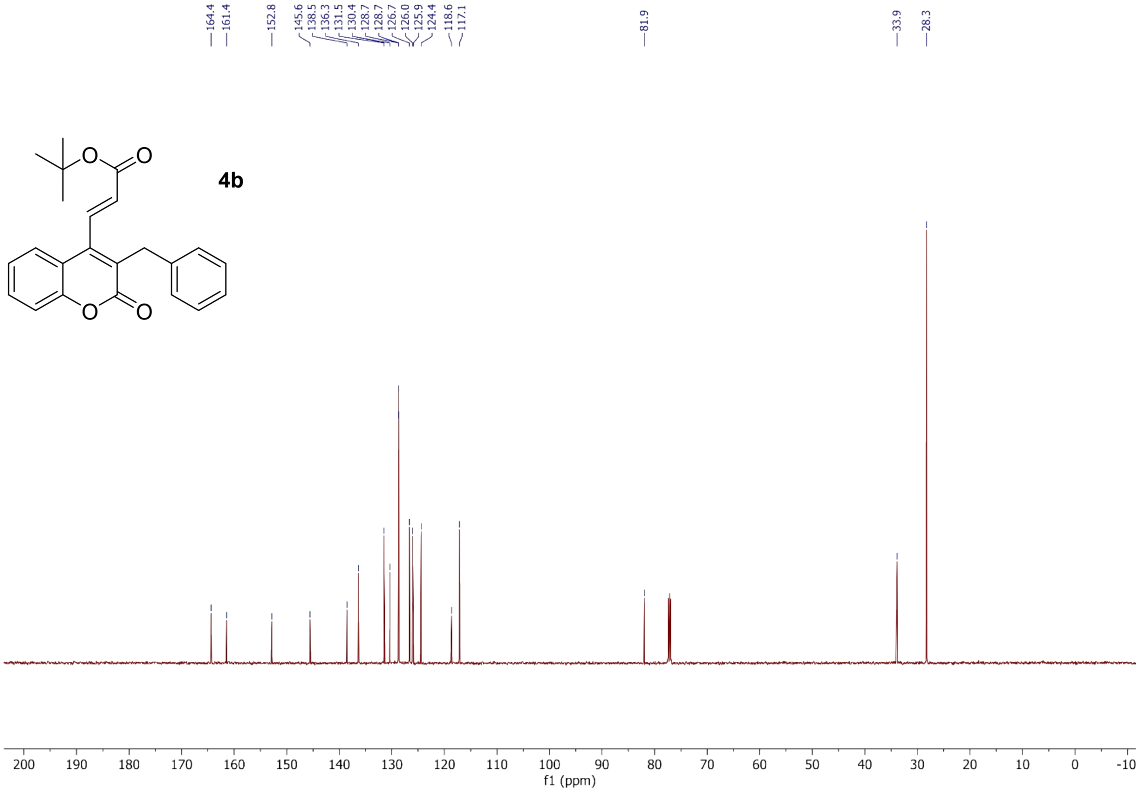

4b

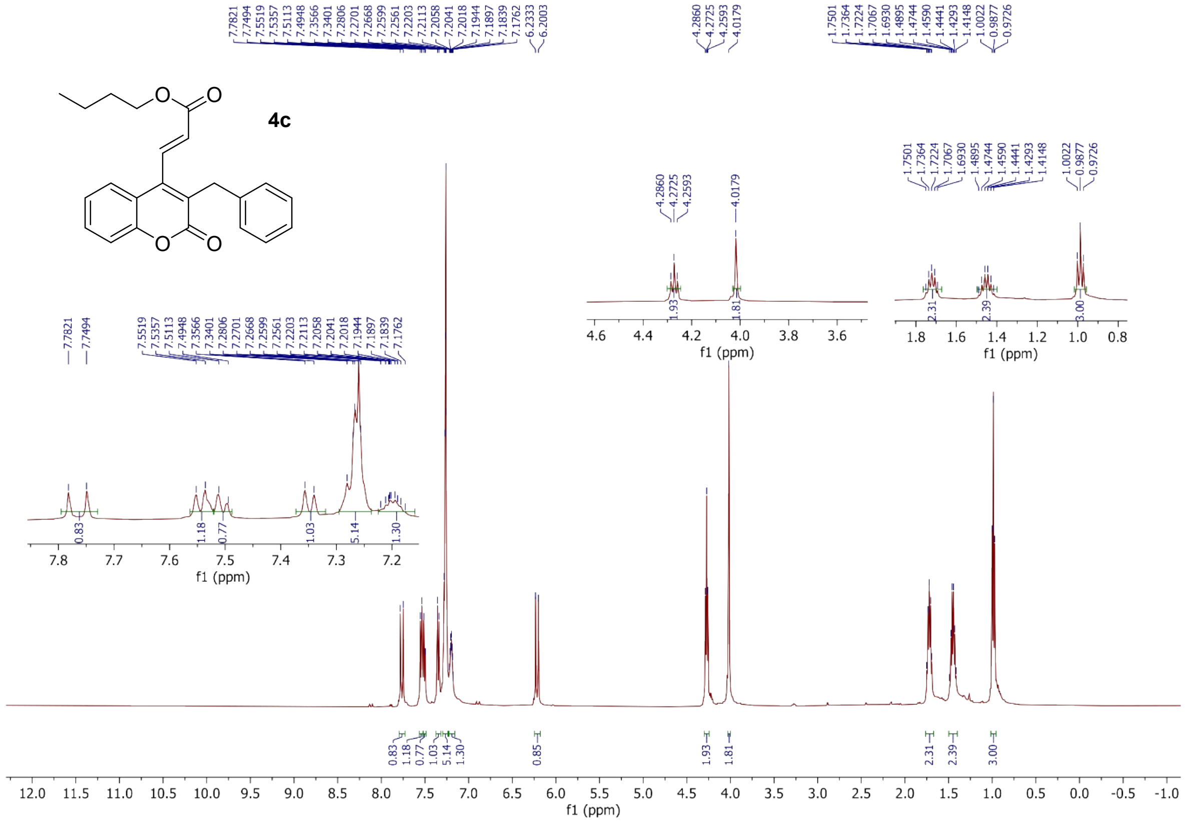
4c
f1 (ppm)

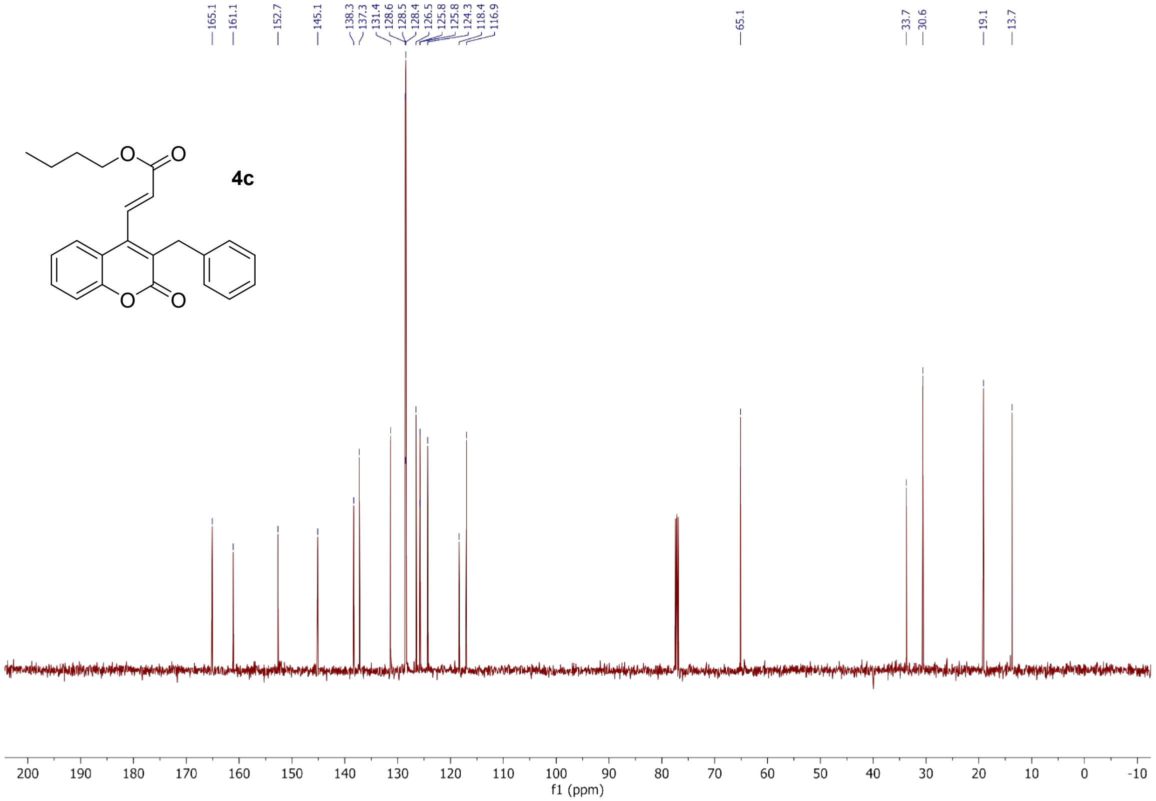
4c
f1 (ppm)

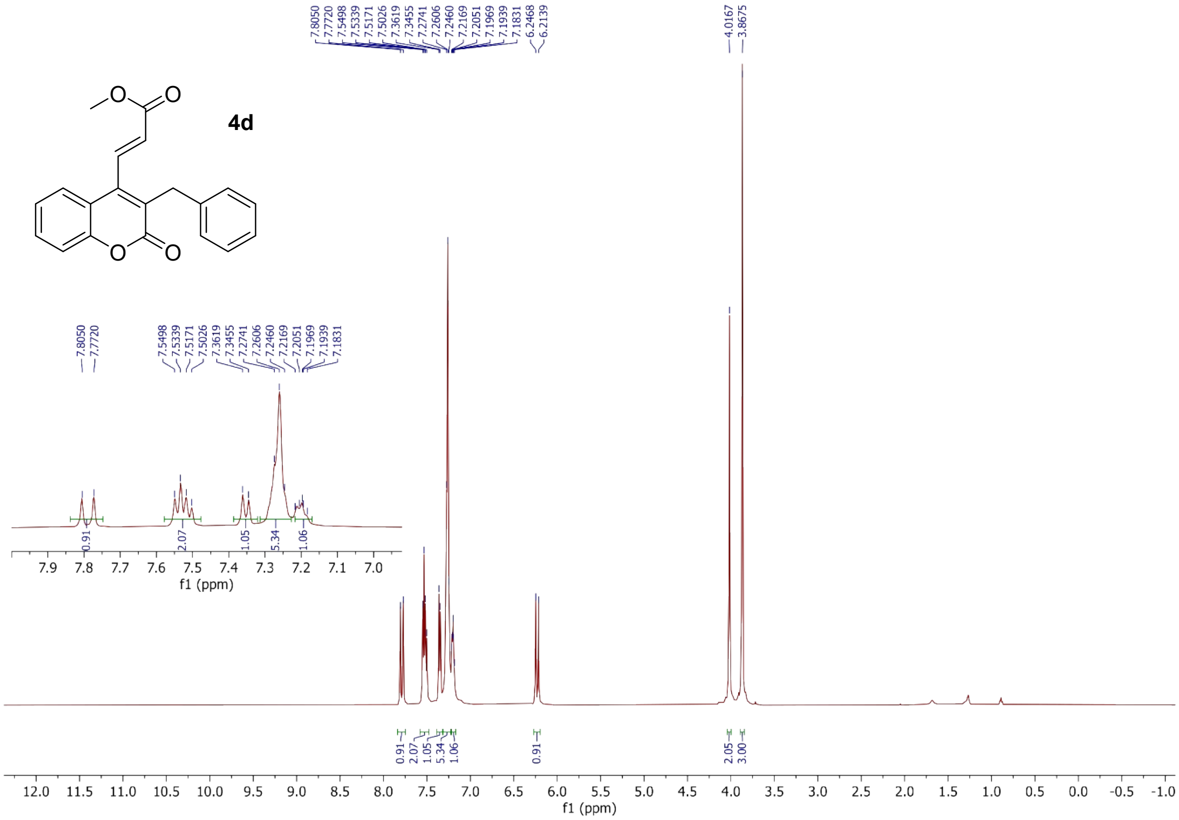
4d
7.8050
7.7720
7.5498
7.5339
7.5171
7.5026
7.3619
7.3455
7.2741
7.2606
7.2460
7.2169
7.2051
7.1969
7.1939
7.1831
6.2468
6.2139
4.0167
3.8675
0.91
2.07
1.05
5.34
1.06
0.91
2.05
3.00
f1 (ppm)

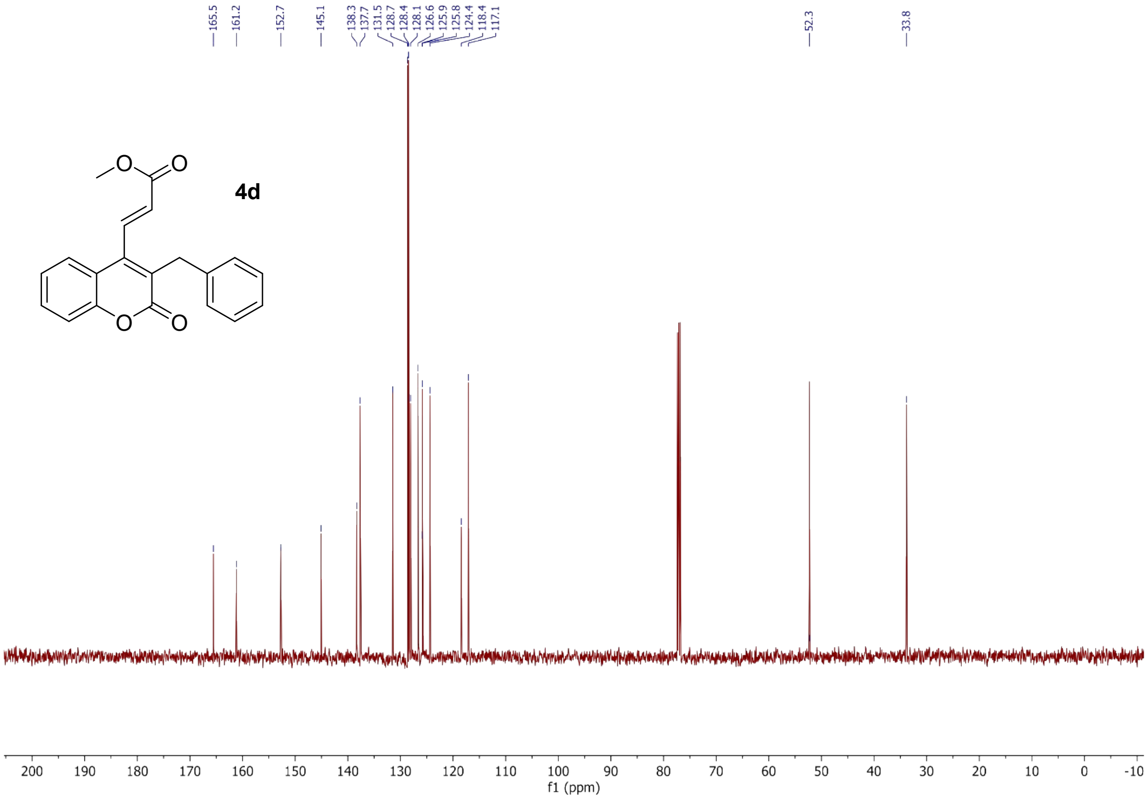
4d
165.5
161.2
152.7
145.1
138.3
137.7
131.5
128.7
128.4
128.1
126.6
125.9
125.8
124.4
118.4
117.1
52.3
33.8
f1 (ppm)

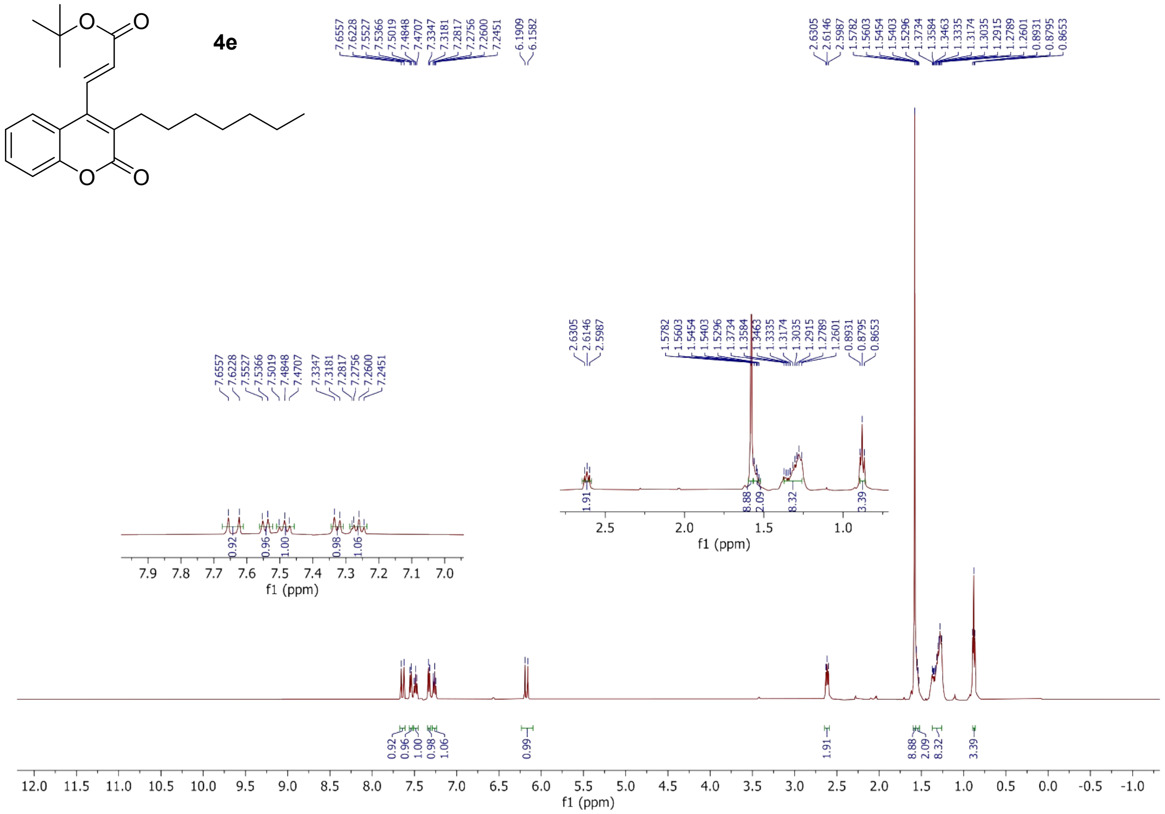
4e
f1 (ppm)

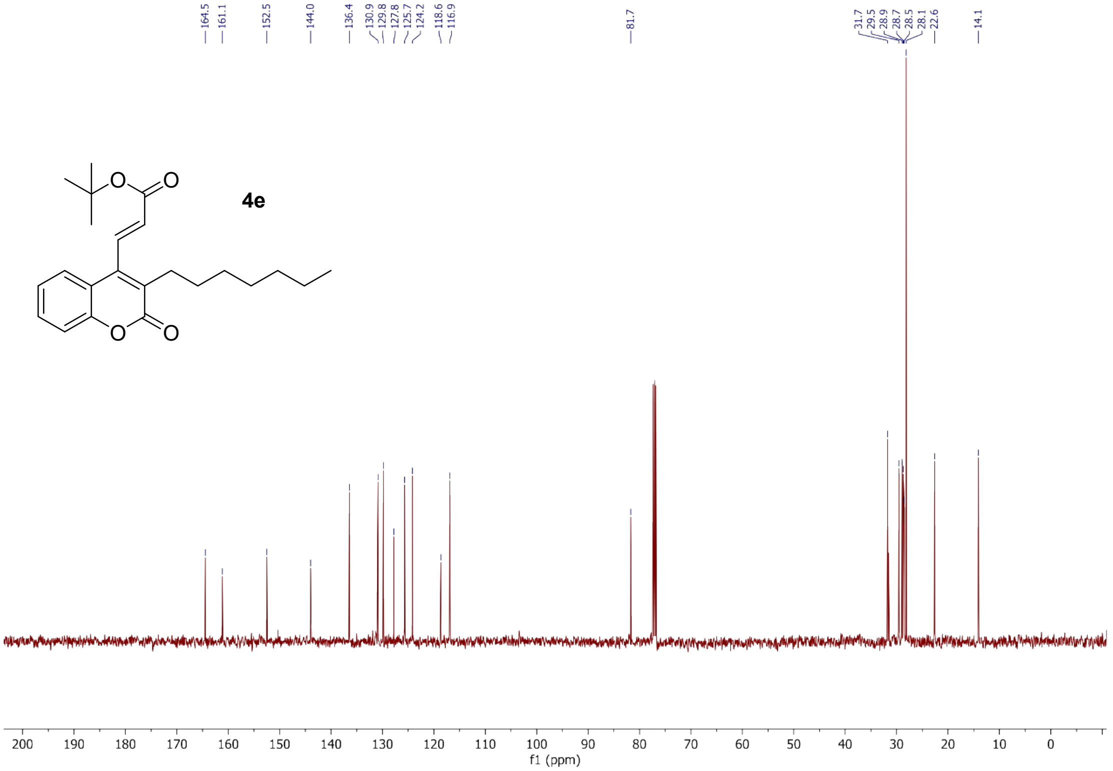
164.5
161.1
152.5
144.0
136.4
130.9
129.8
127.8
125.7
124.2
118.6
116.9
81.7
31.7
29.5
28.9
28.7
28.5
28.1
22.6
14.1
4e
f1 (ppm)

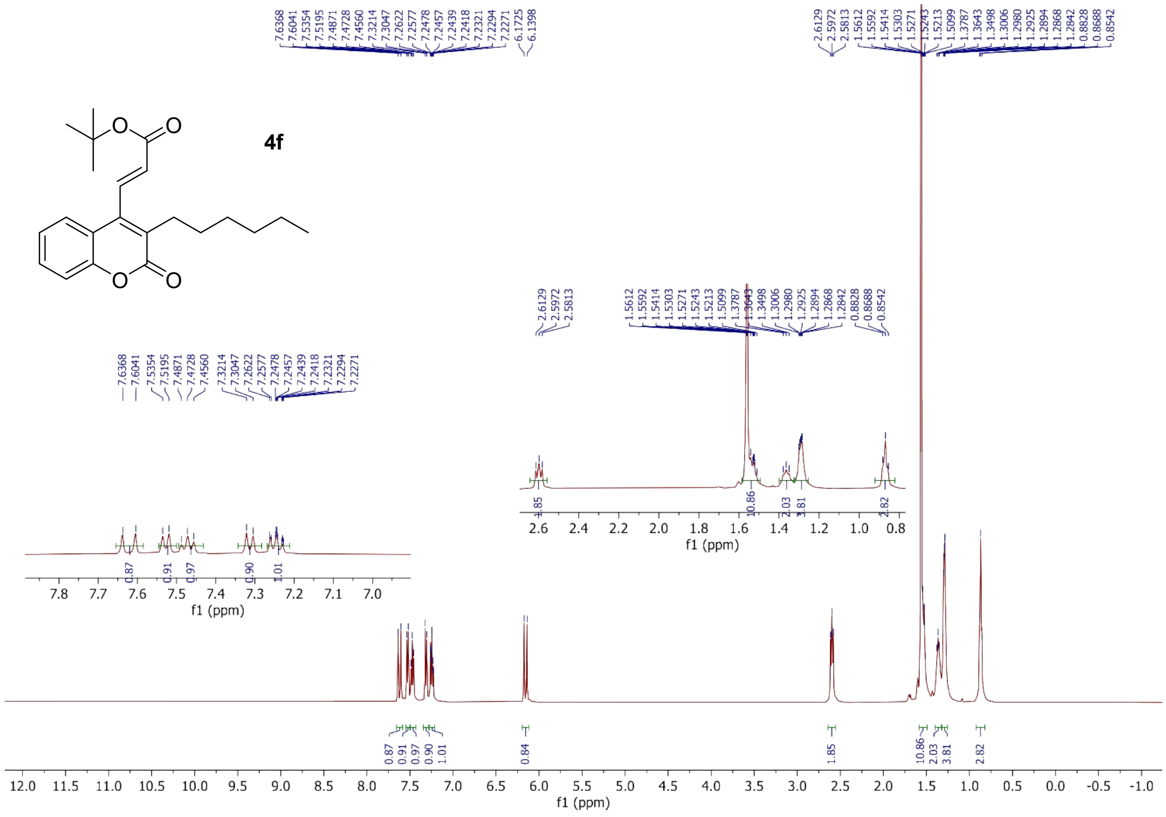
4f
7.6368
7.6041
7.5354
7.5195
7.4871
7.4728
7.4560
7.3214
7.3047
7.2622
7.2577
7.2478
7.2457
7.2439
7.2418
7.2321
7.2294
7.2271
6.1725
6.1398
2.6129
2.5972
2.5813
1.5612
1.5592
1.5414
1.5303
1.5271
1.5243
1.5213
1.5099
1.3787
1.3643
1.3498
1.3006
1.2980
1.2925
1.2894
1.2868
1.2842
0.8828
0.8688
0.8542
0.87
0.91
0.97
0.90
1.01
0.84
1.85
10.86
2.03
3.81
2.82
f1 (ppm)

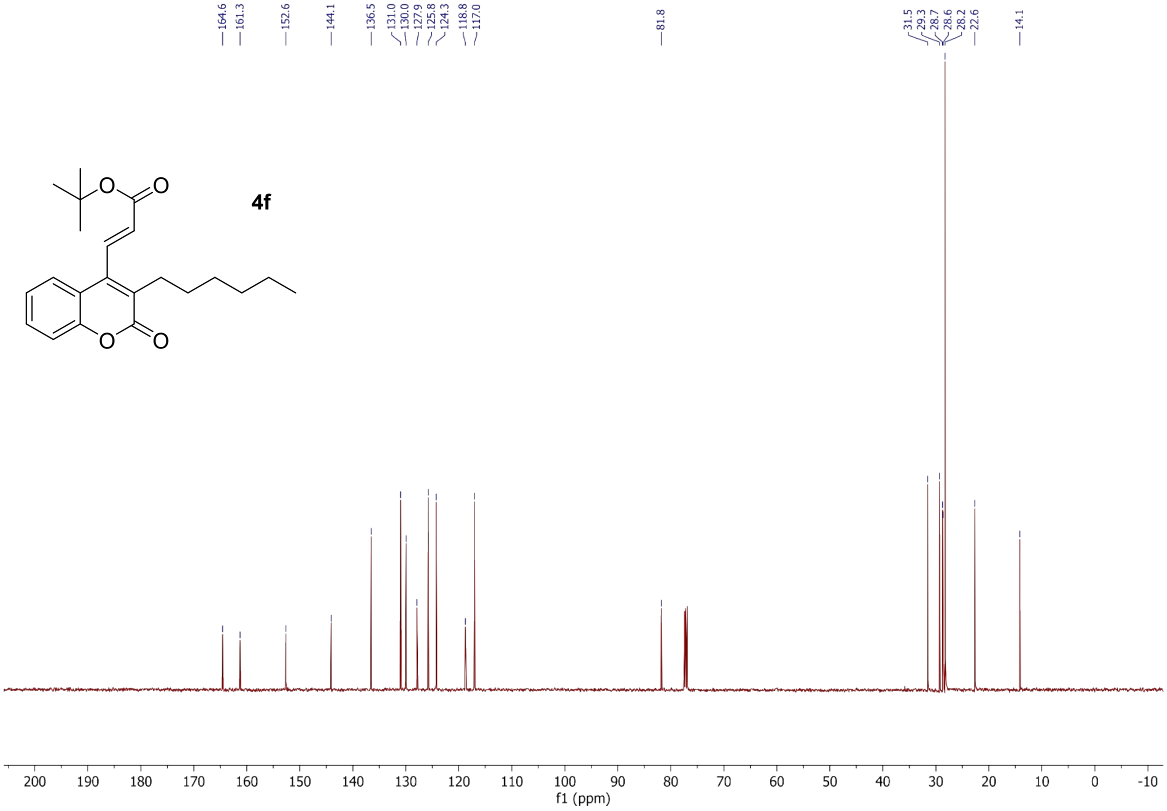
4f
164.6
161.3
152.6
144.1
136.5
131.0
130.0
127.9
125.8
124.3
118.8
117.0
81.8
31.5
29.3
28.7
28.6
28.2
22.6
14.1
f1 (ppm)

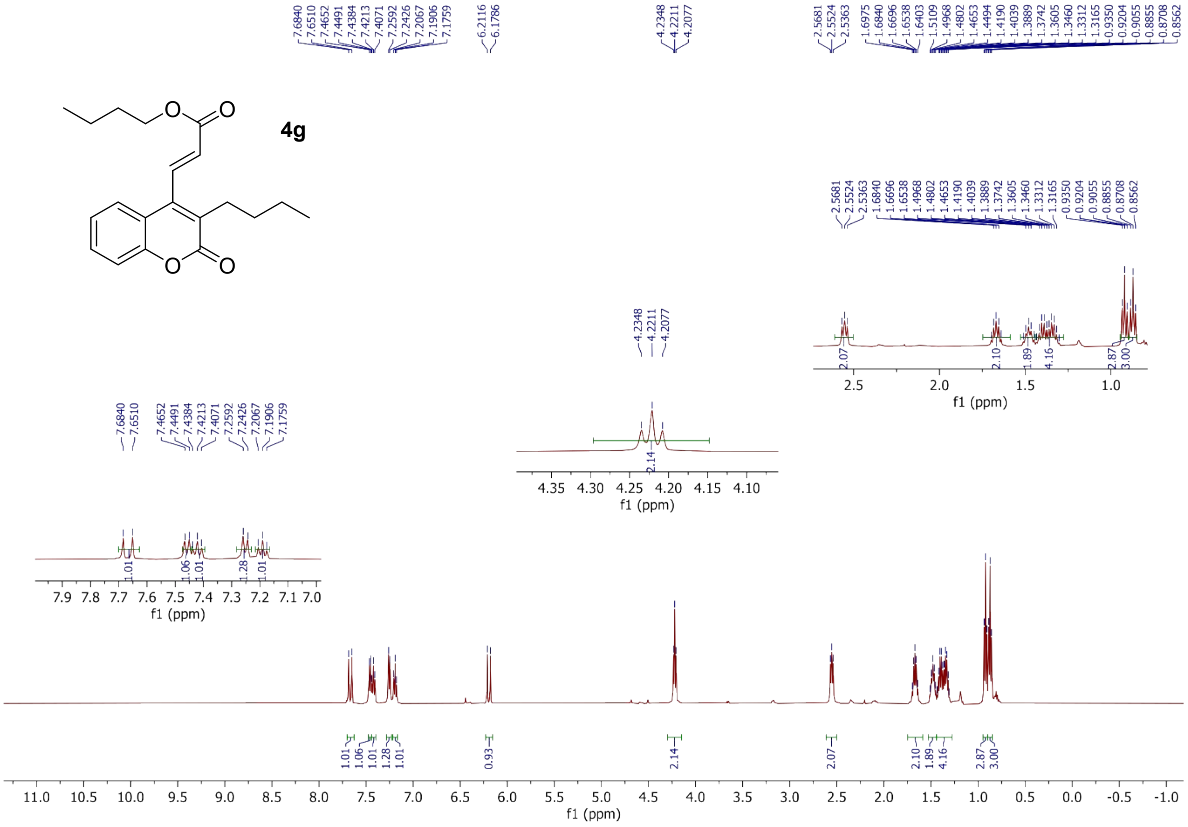

7.6840 7.6510 7.4652 7.4491 7.4384 7.4213 7.4071 7.2592 7.2426 7.2067 7.1906 7.1759
6.2116 6.1786
4.2348 4.2211 4.2077
2.5681 2.5524 2.5363 1.6975 1.6840 1.6696 1.6538 1.6403 1.5109 1.4968 1.4802 1.4653 1.4494 1.4190 1.4039 1.3889 1.3742 1.3605 1.3460 1.3312 1.3165 0.9350 0.9204 0.9055 0.8855 0.8708 0.8562
4g
f1 (ppm)

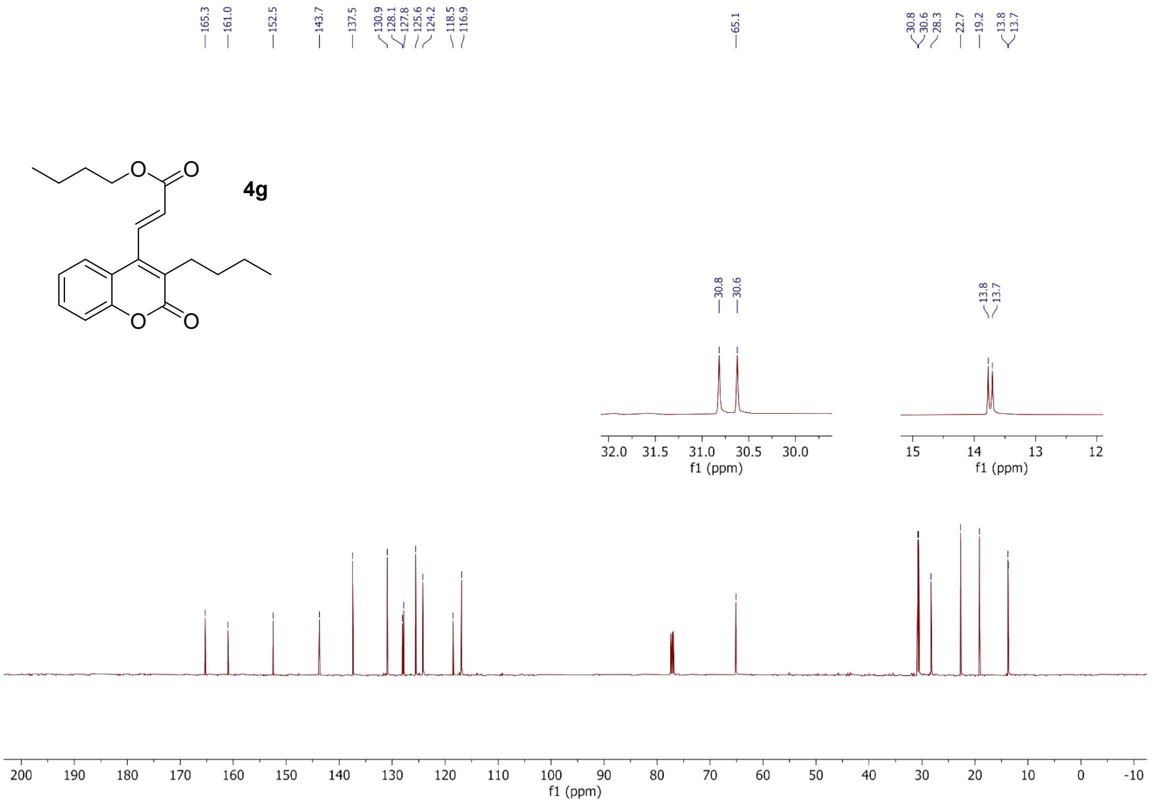

165.3 161.0 152.5 143.7 137.5 130.9 128.1 127.8 125.6 124.2 118.5 116.9
65.1
30.8 30.6 28.3 22.7 19.2 13.8 13.7
4g
f1 (ppm)

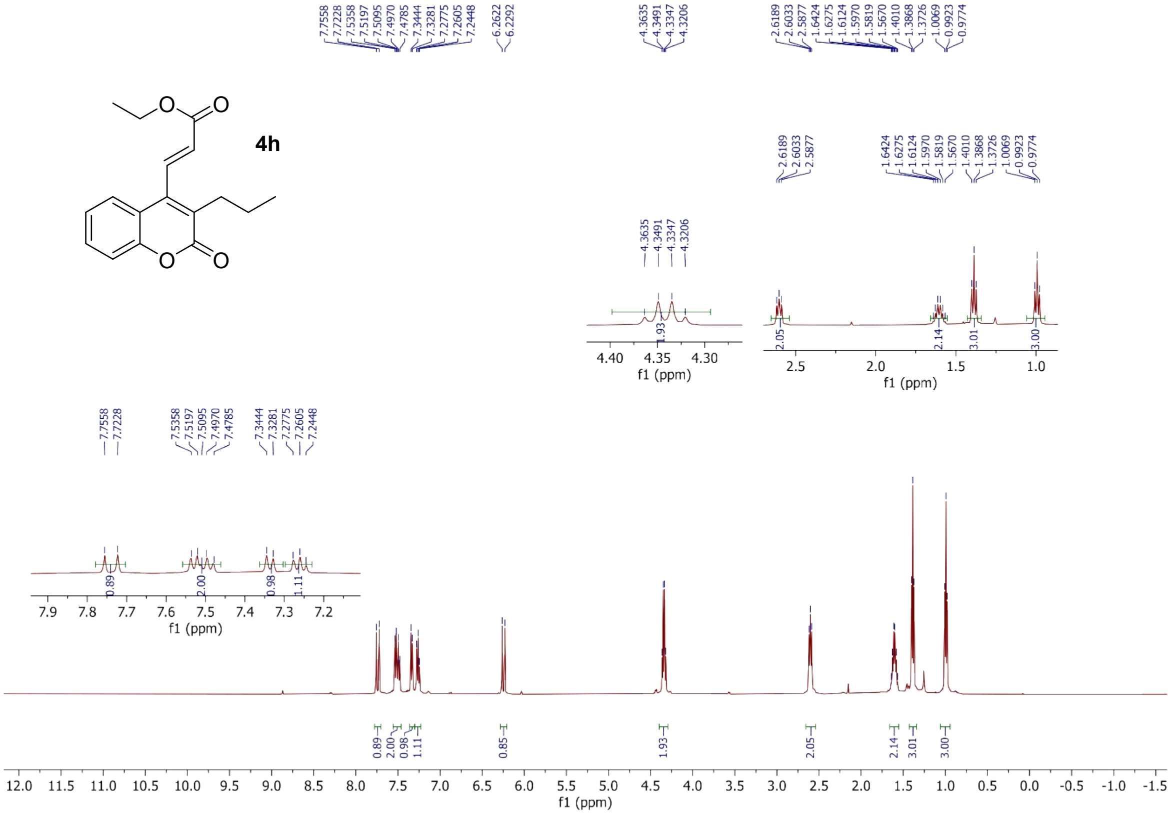
4h
7.7558
7.7228
7.5358
7.5197
7.5095
7.4970
7.4785
7.3444
7.3281
7.2775
7.2605
7.2448
6.2622
6.2292
4.3635
4.3491
4.3347
4.3206
2.6189
2.6033
2.5877
1.6424
1.6275
1.6124
1.5970
1.5819
1.5670
1.4010
1.3868
1.3726
1.0069
0.9923
0.9774
0.89
2.00
0.98
1.11
0.85
1.93
2.05
2.14
3.01
3.00
f1 (ppm)

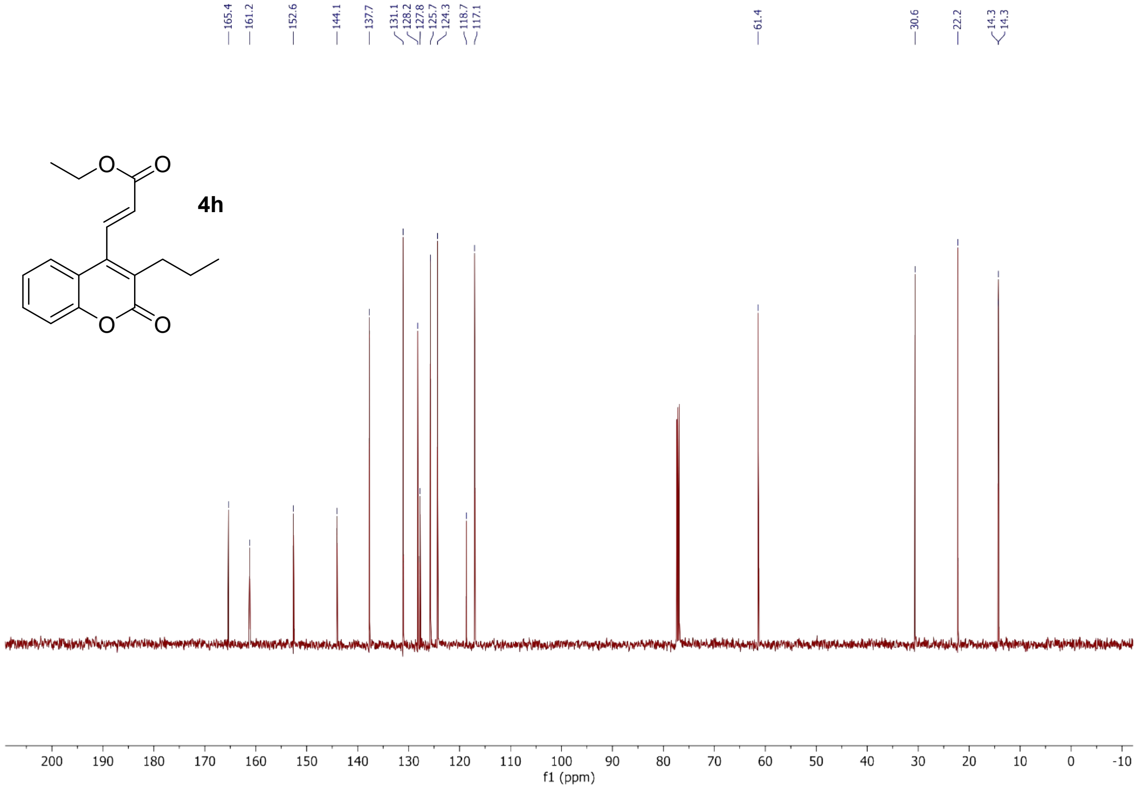
4h
165.4
161.2
152.6
144.1
137.7
131.1
128.2
127.8
125.7
124.3
118.7
117.1
61.4
30.6
22.2
14.3
14.3
f1 (ppm)

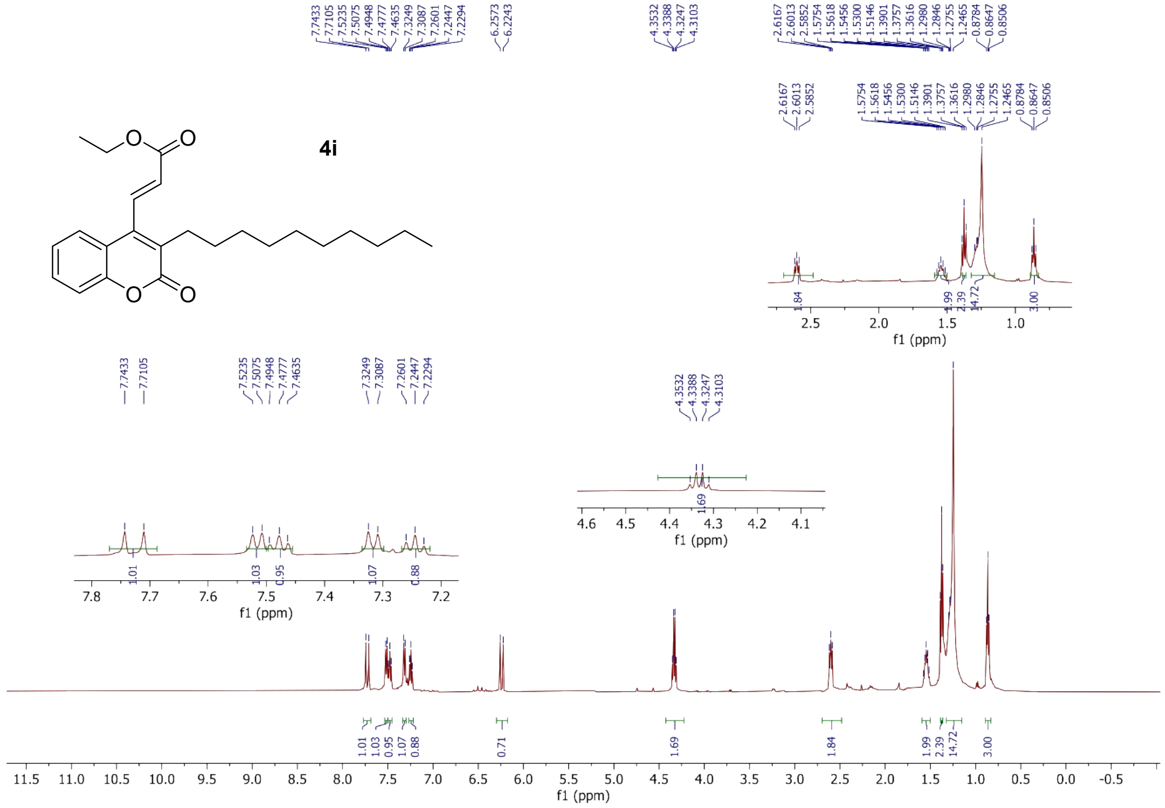
4i
7.7433
7.7105
7.5235
7.5075
7.4948
7.4777
7.4635
7.3249
7.3087
7.2601
7.2447
7.2294
6.2573
6.2243
4.3532
4.3388
4.3247
4.3103
2.6167
2.6013
2.5852
1.5754
1.5618
1.5456
1.5300
1.5146
1.3901
1.3757
1.3616
1.2980
1.2846
1.2755
1.2465
0.8784
0.8647
0.8506
1.01
1.03
0.95
1.07
0.88
0.71
1.69
1.84
1.99
2.39
14.72
3.00
f1 (ppm)

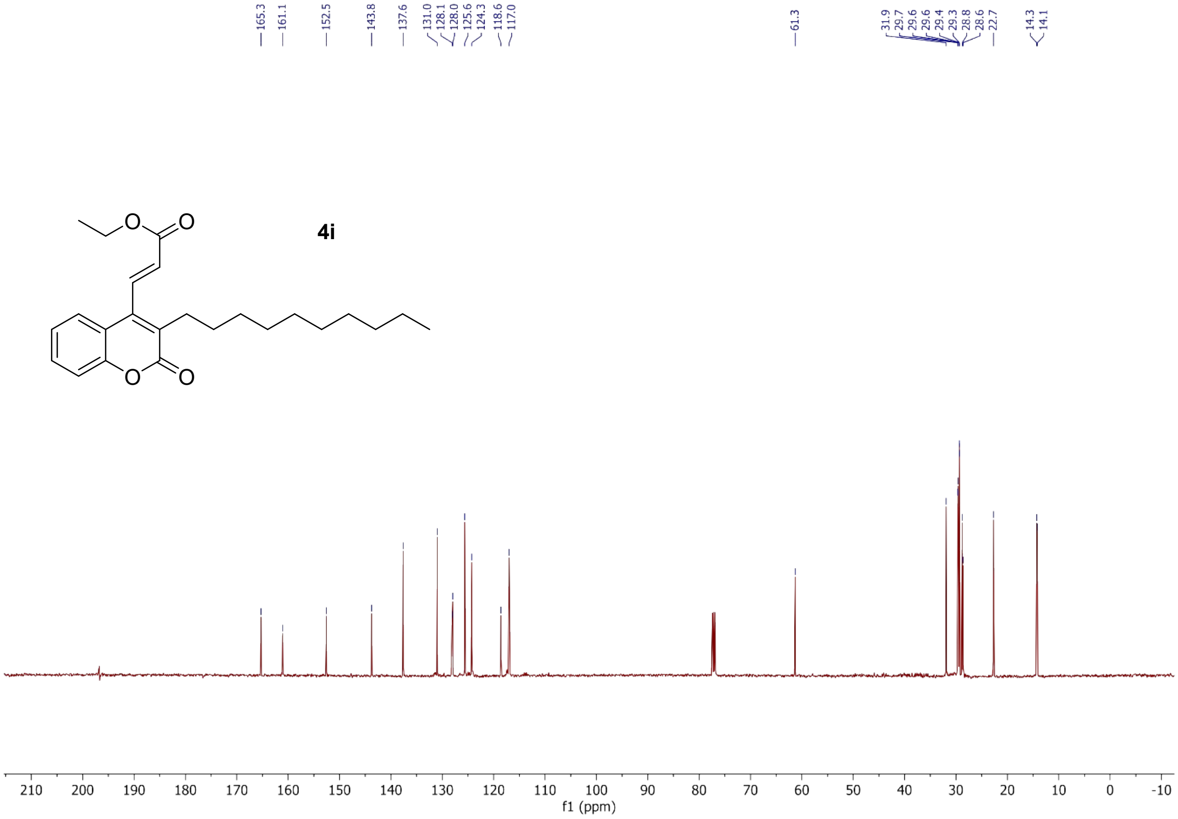
4i
165.3
161.1
152.5
143.8
137.6
131.0
128.1
128.0
125.6
124.3
118.6
117.0
61.3
31.9
29.7
29.6
29.6
29.4
29.3
28.8
28.6
22.7
14.3
14.1
f1 (ppm)

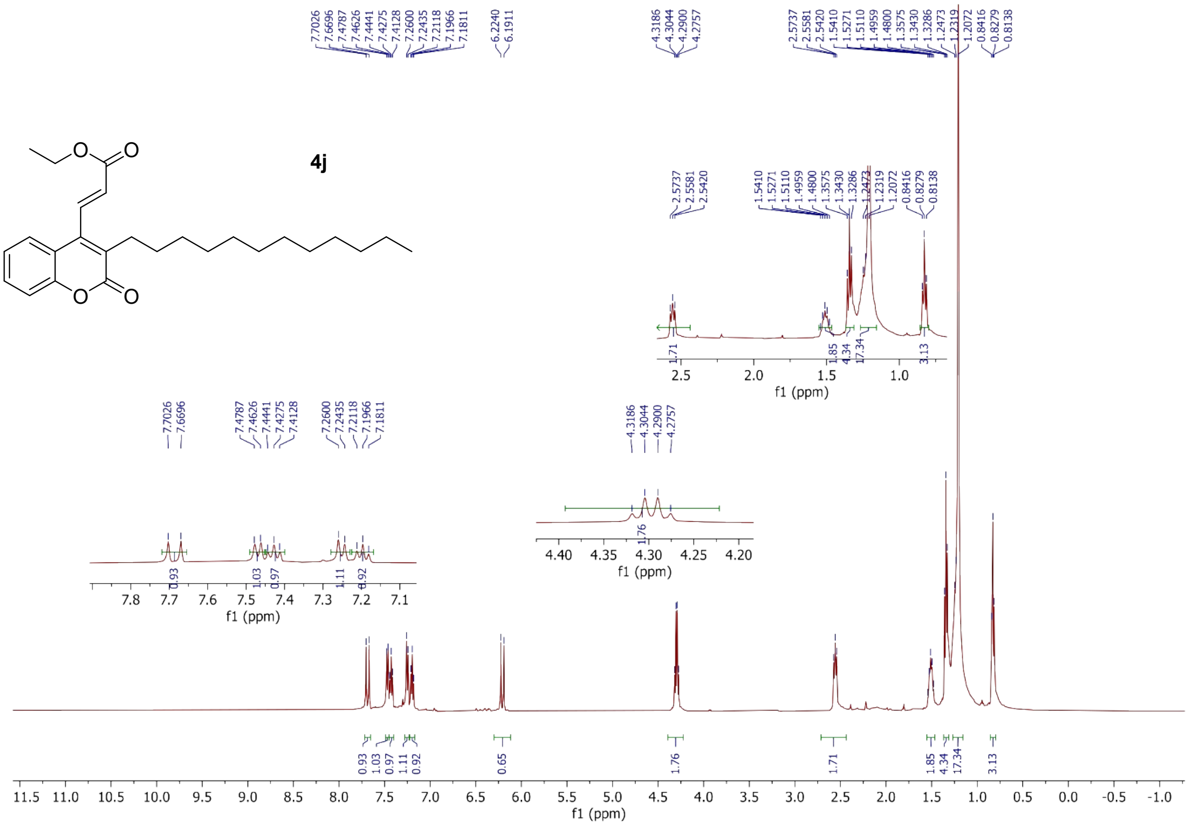

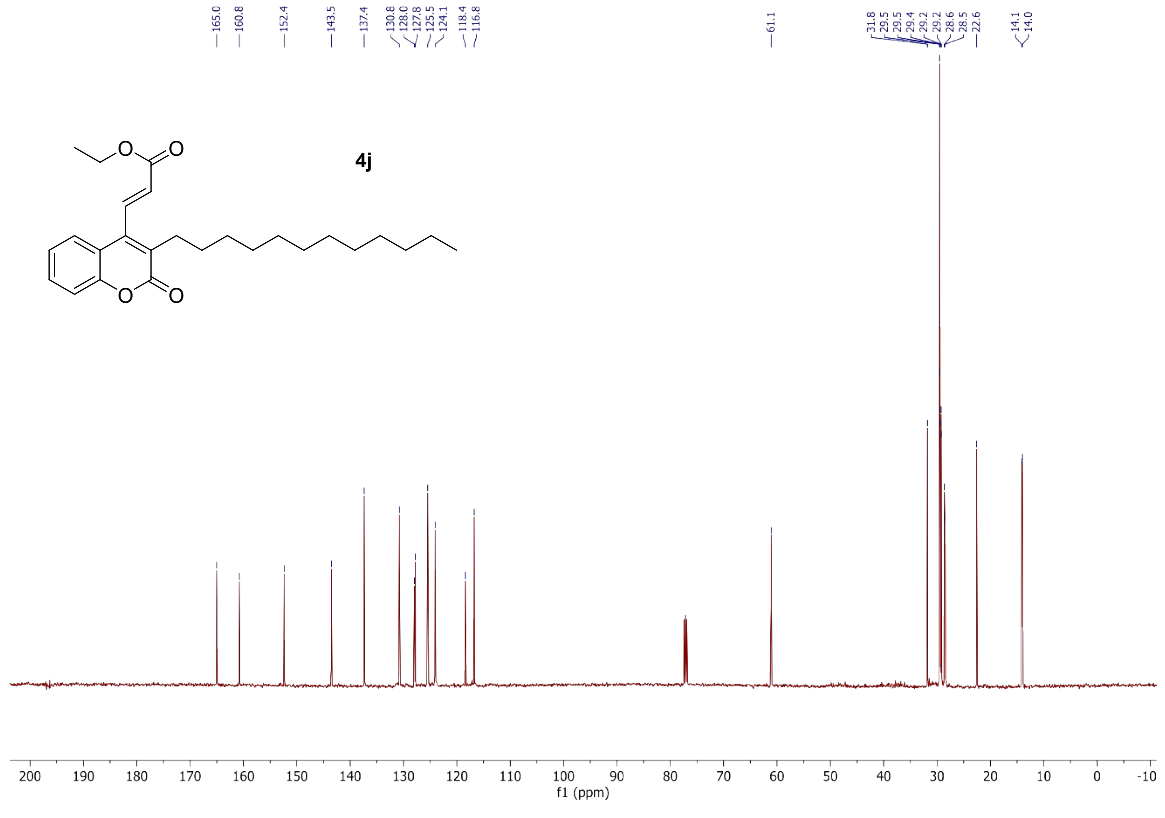

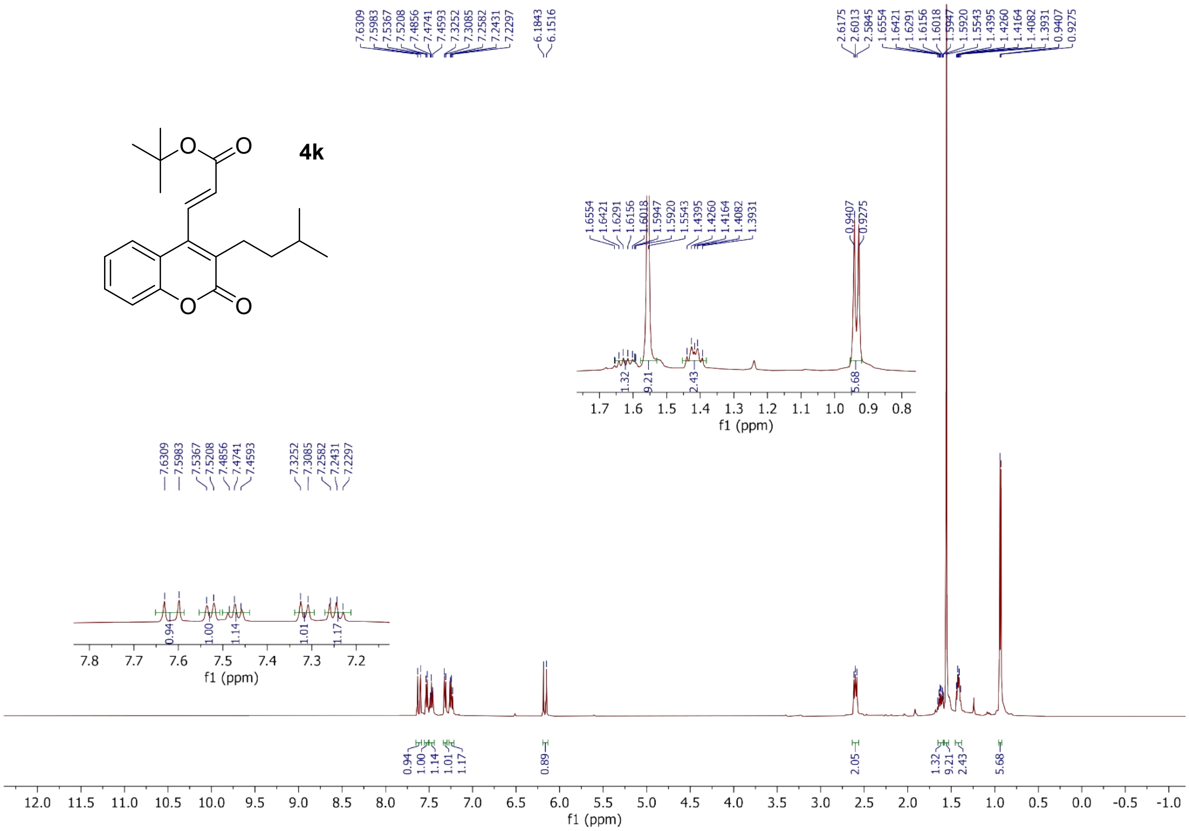
4k
7.6309
7.5983
7.5367
7.5208
7.4856
7.4741
7.4593
7.3252
7.3085
7.2582
7.2431
7.2297
6.1843
6.1516
2.6175
2.6013
2.5845
1.6554
1.6421
1.6291
1.6156
1.6018
1.5947
1.5920
1.5543
1.4395
1.4260
1.4164
1.4082
1.3931
0.9407
0.9275
0.94
1.00
1.14
1.01
1.17
0.89
2.05
1.32
9.21
2.43
5.68
f1 (ppm)

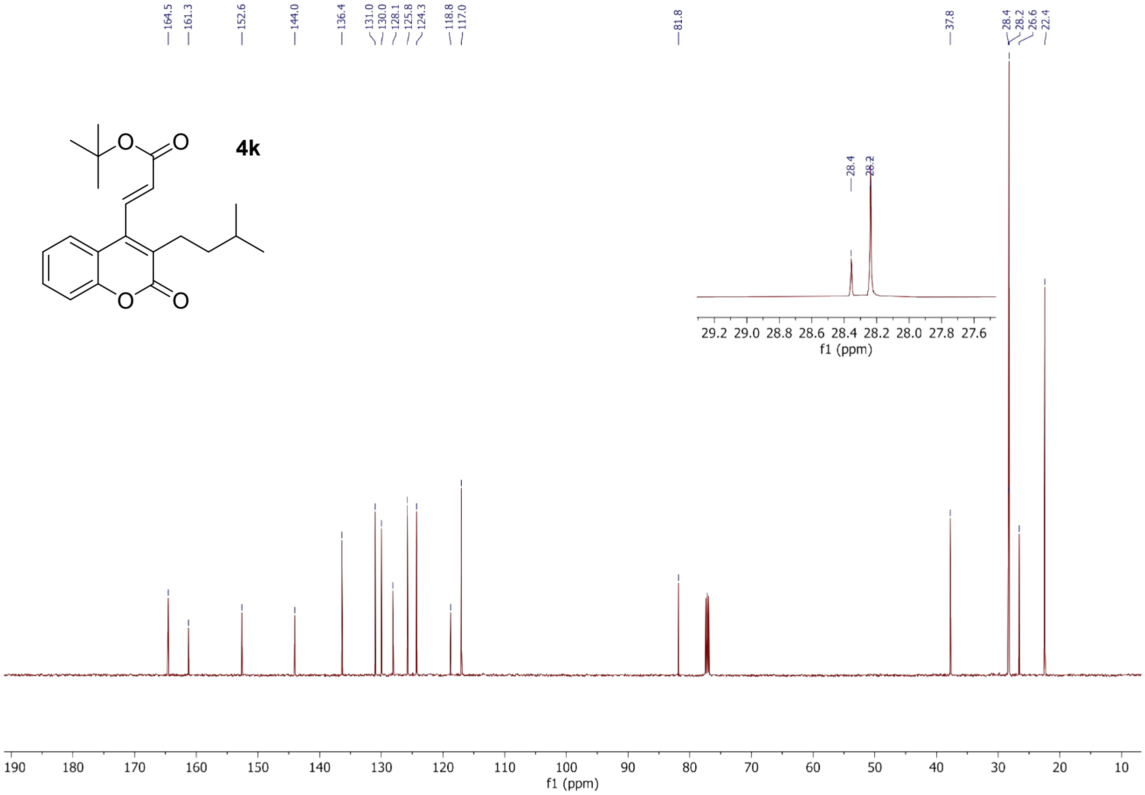
4k
164.5
161.3
152.6
144.0
136.4
131.0
130.0
128.1
125.8
124.3
118.8
117.0
81.8
37.8
28.4
28.2
26.6
22.4
f1 (ppm)

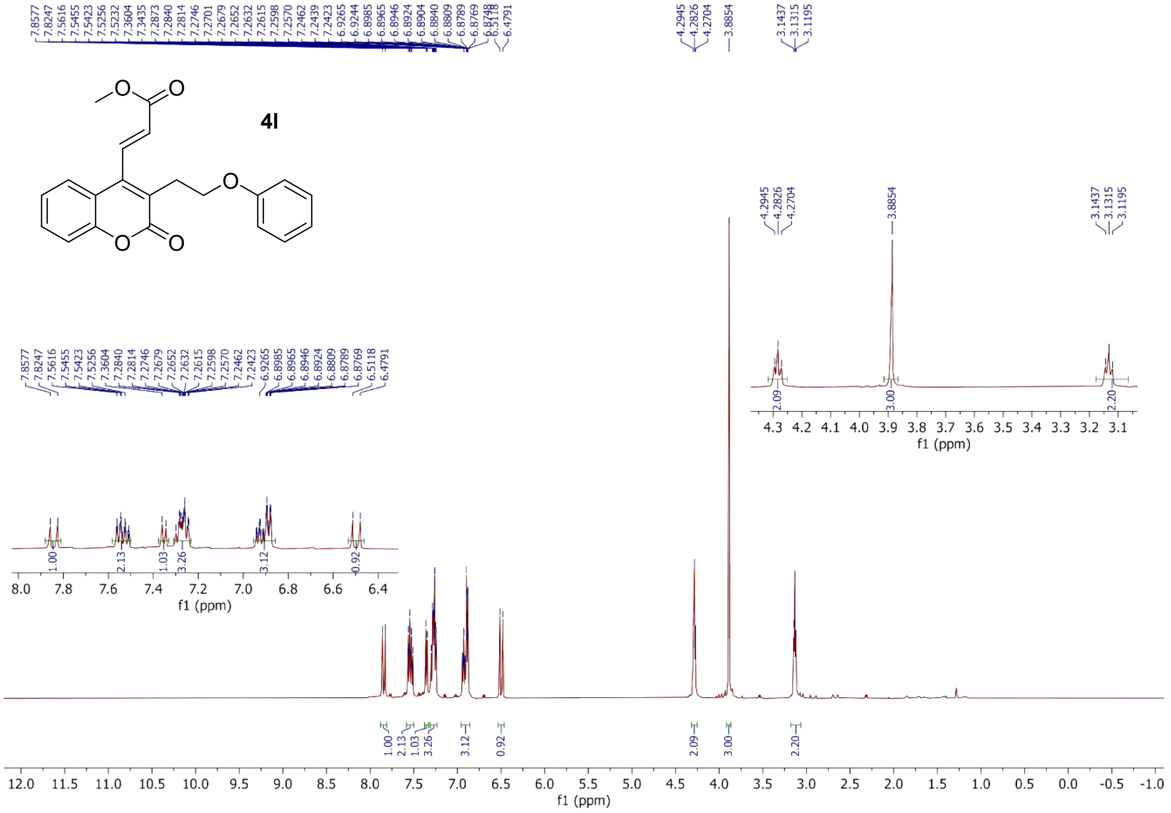

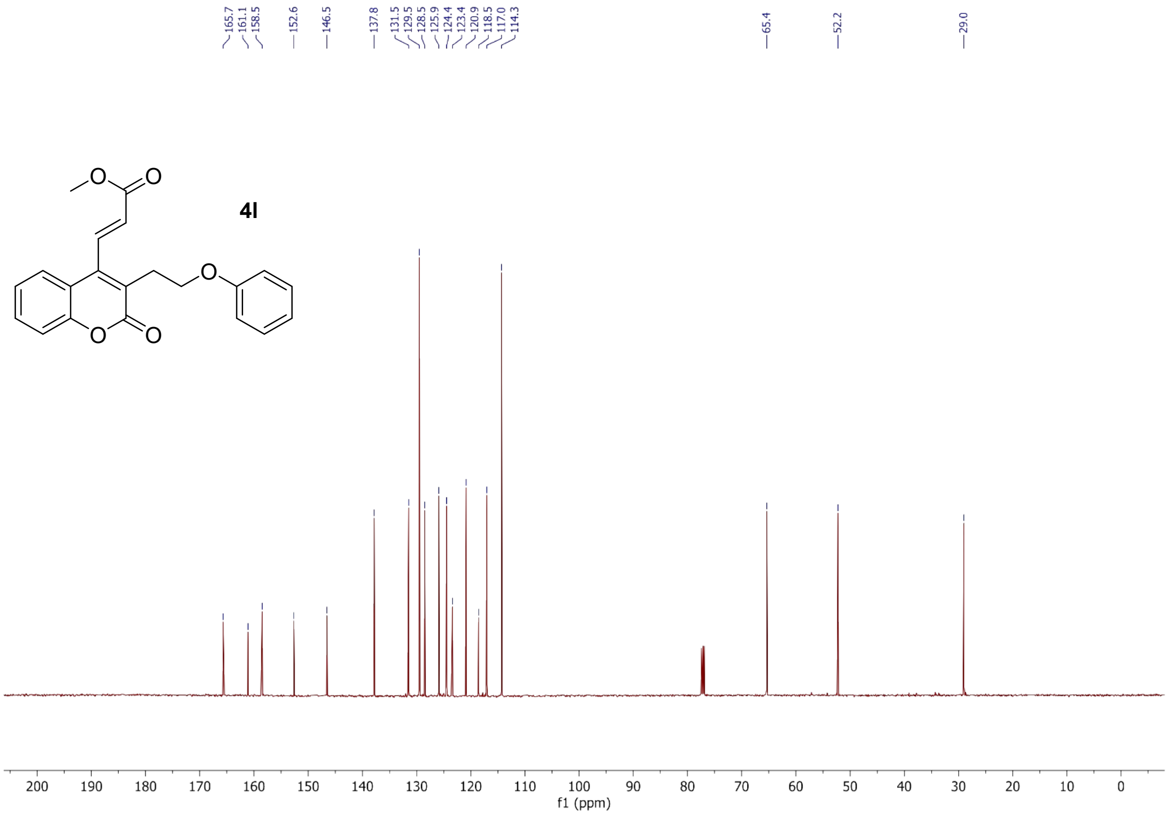

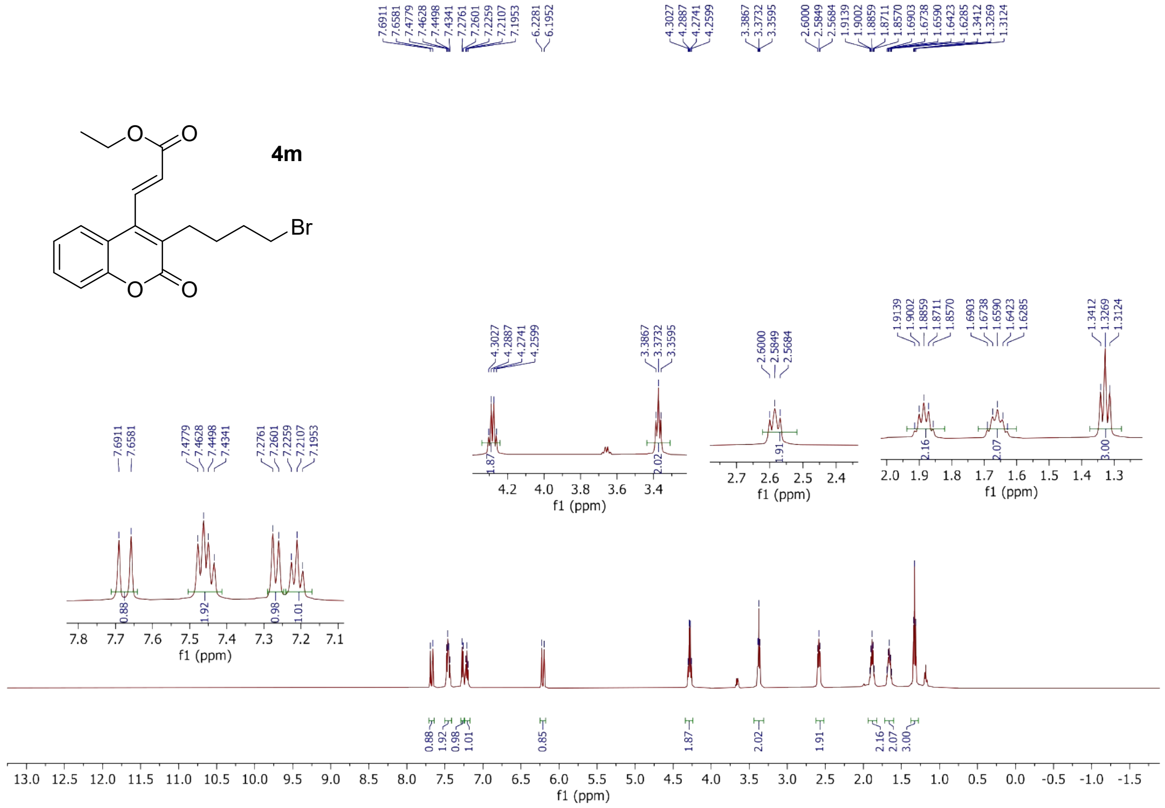
4m
f1 (ppm)

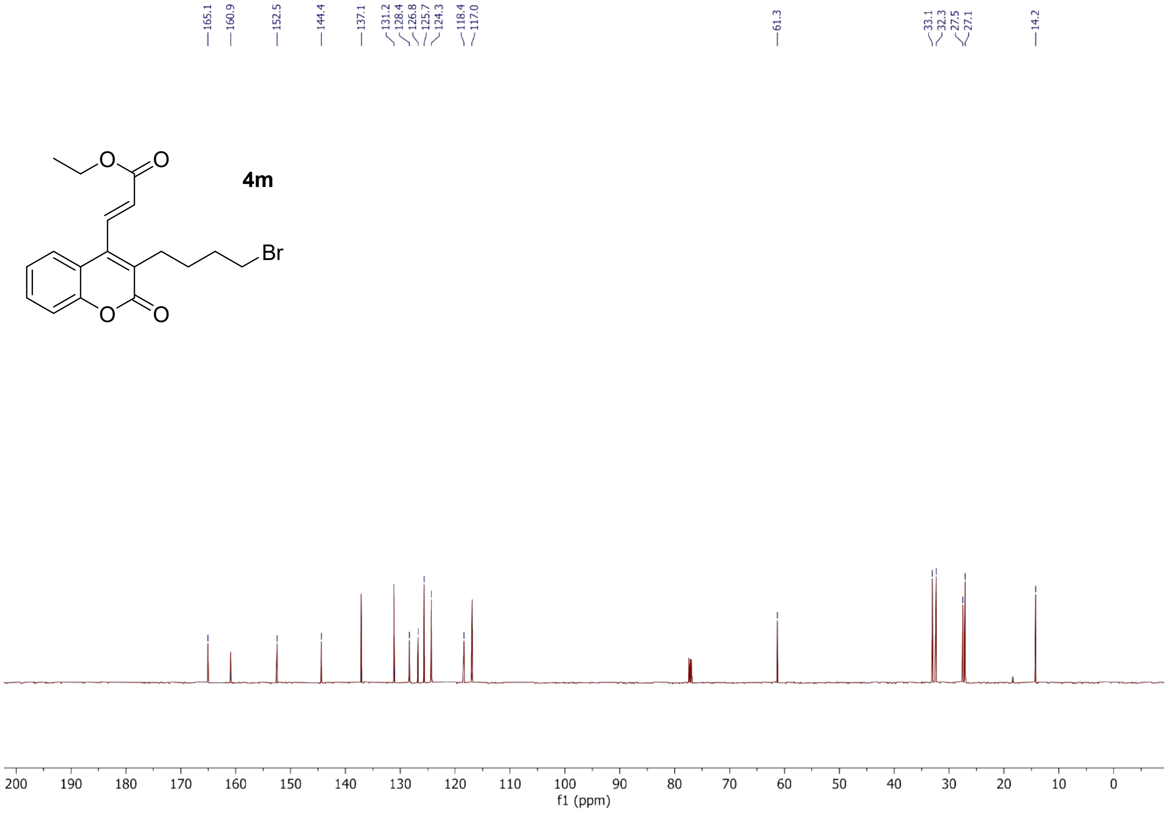
4m
f1 (ppm)

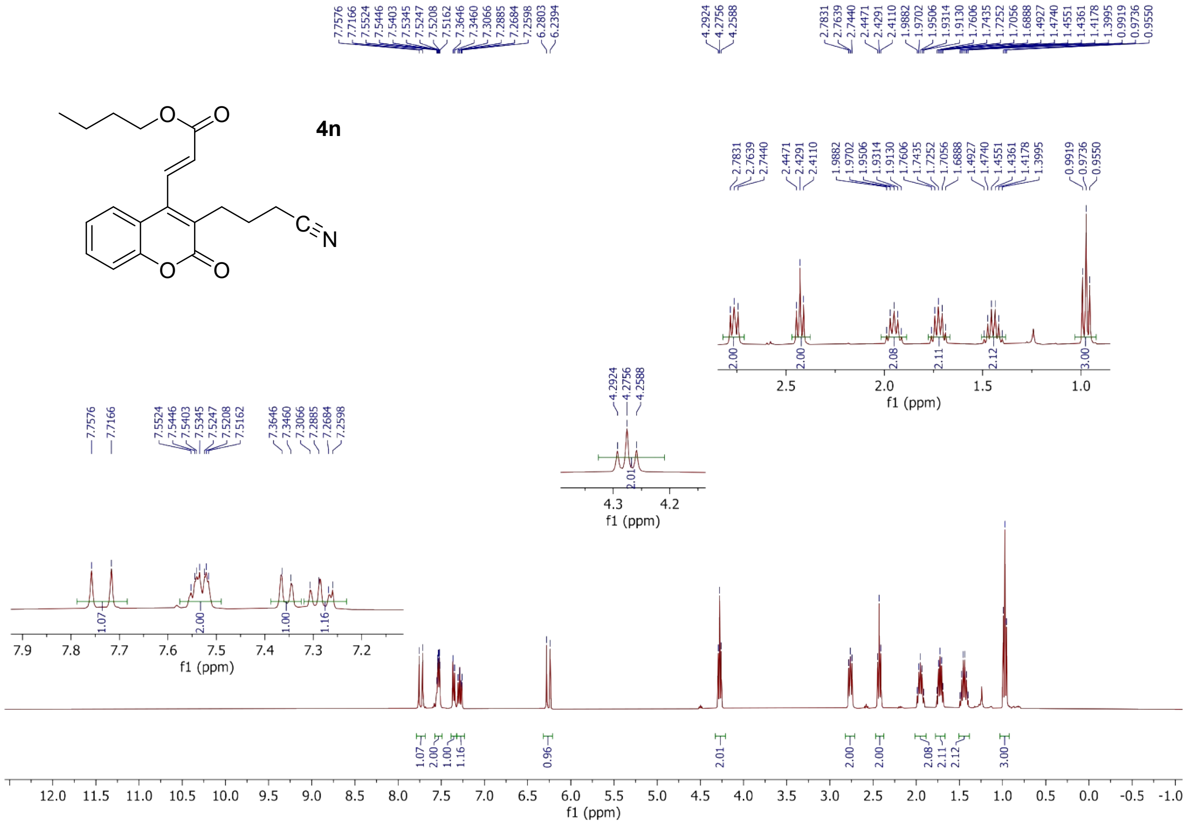
4n
7.7576 7.7166 7.5524 7.5446 7.5403 7.5345 7.5247 7.5208 7.5162 7.3646 7.3460 7.3066 7.2885 7.2684 7.2598 6.2803 6.2394
4.2924 4.2756 4.2588
2.7831 2.7639 2.7440 2.4471 2.4291 2.4110 1.9882 1.9702 1.9506 1.9314 1.9130 1.7606 1.7435 1.7252 1.7056 1.6888 1.4927 1.4740 1.4551 1.4361 1.4178 1.3995 0.9919 0.9736 0.9550
1.07 2.00 1.00 1.16 0.96 2.01 2.00 2.00 2.08 2.11 2.12 3.00
f1 (ppm)

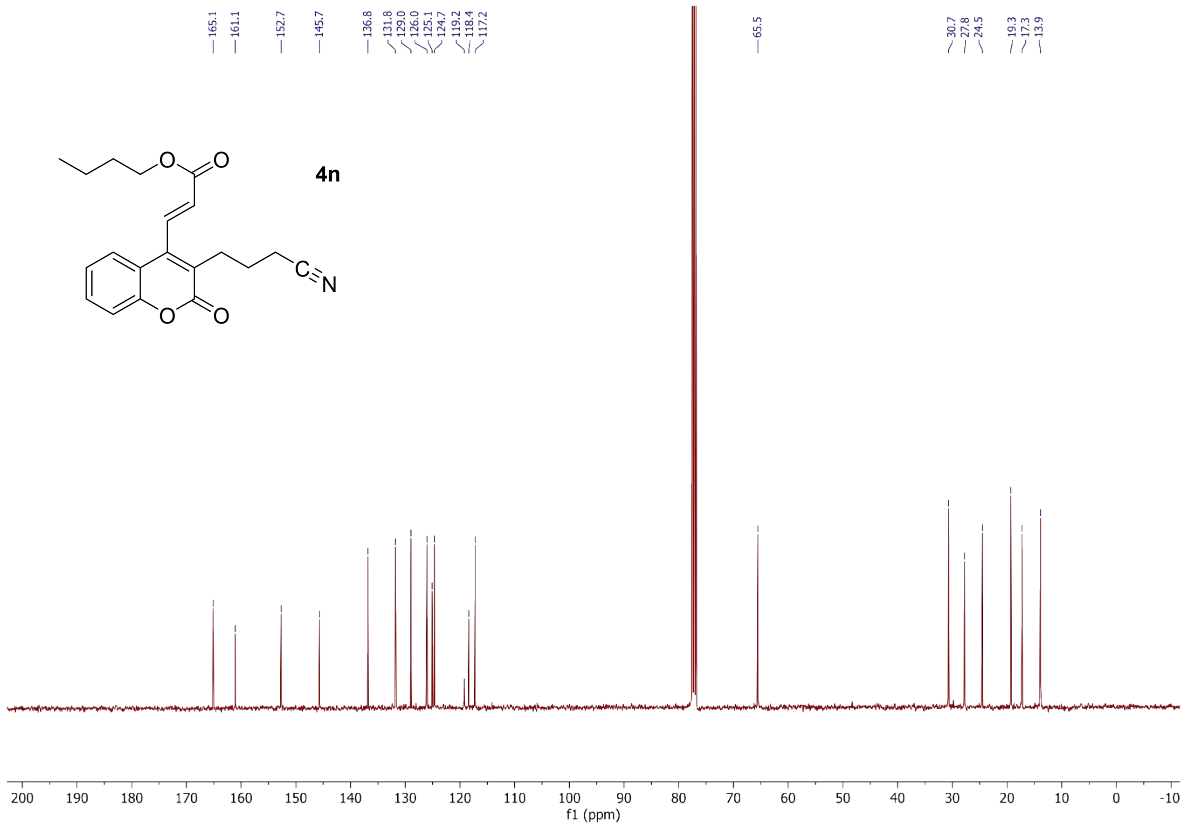
4n
165.1 161.1 152.7 145.7 136.8 131.8 129.0 126.0 125.1 124.7 119.2 118.4 117.2 65.5 30.7 27.8 24.5 19.3 17.3 13.9
f1 (ppm)

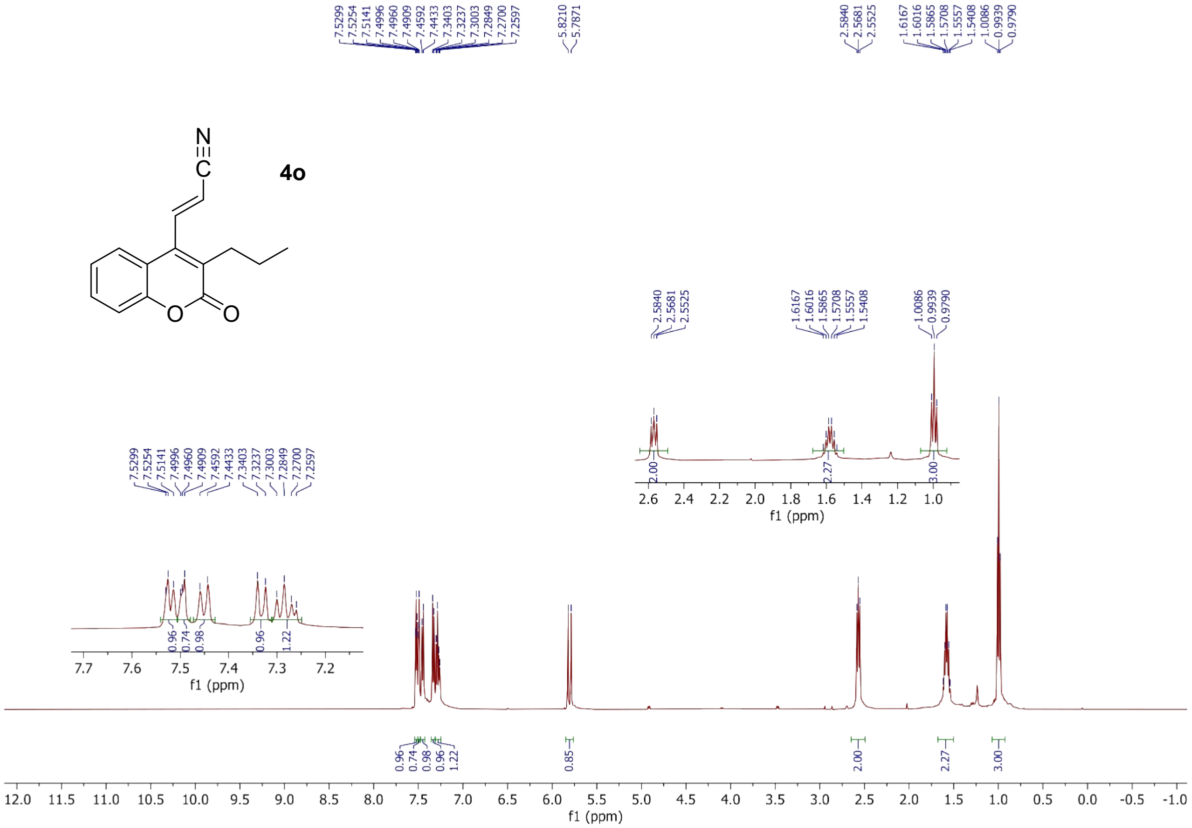
7.5299
7.5254
7.5141
7.4996
7.4960
7.4909
7.4592
7.4433
7.3403
7.3237
7.3003
7.2849
7.2700
7.2597
5.8210
5.7871
2.5840
2.5681
2.5525
1.6167
1.6016
1.5865
1.5708
1.5557
1.5408
1.0086
0.9939
0.9790
4o
f1 (ppm)

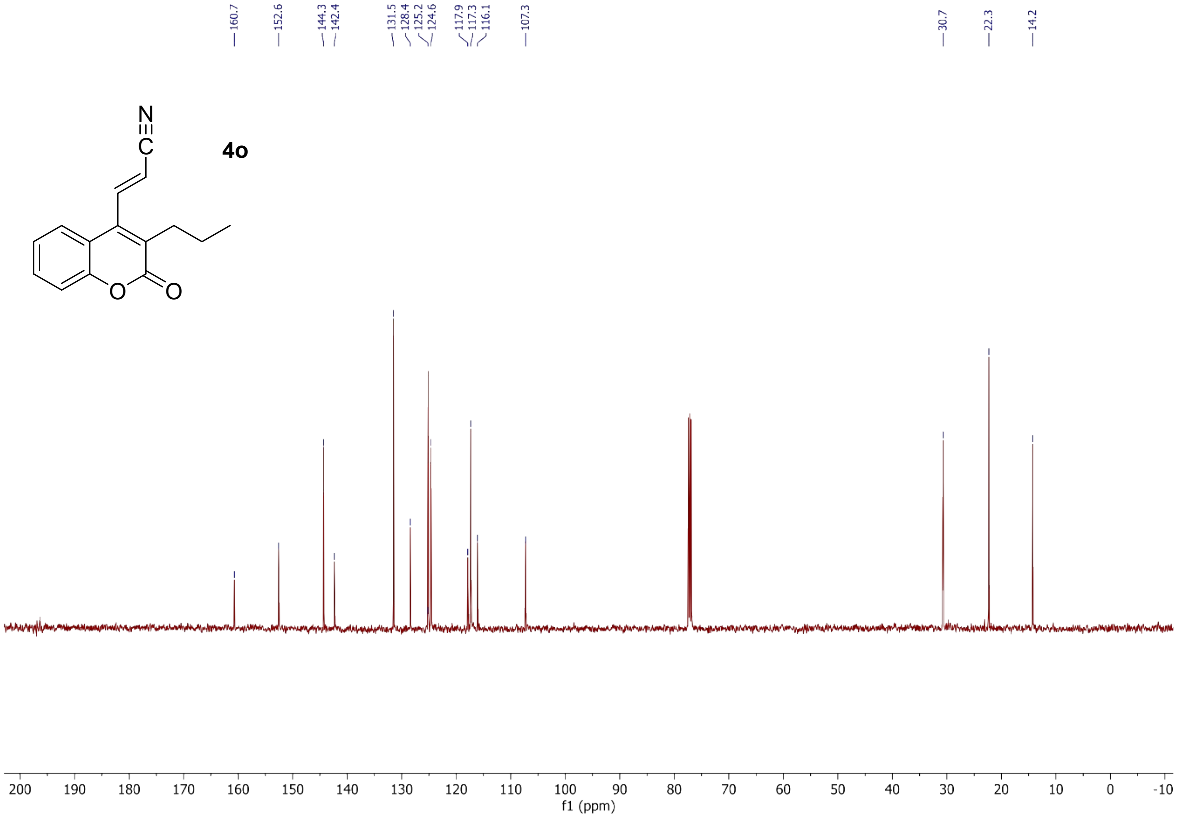
160.7
152.6
144.3
142.4
131.5
128.4
125.2
124.6
117.9
117.3
116.1
107.3
30.7
22.3
14.2
4o
f1 (ppm)

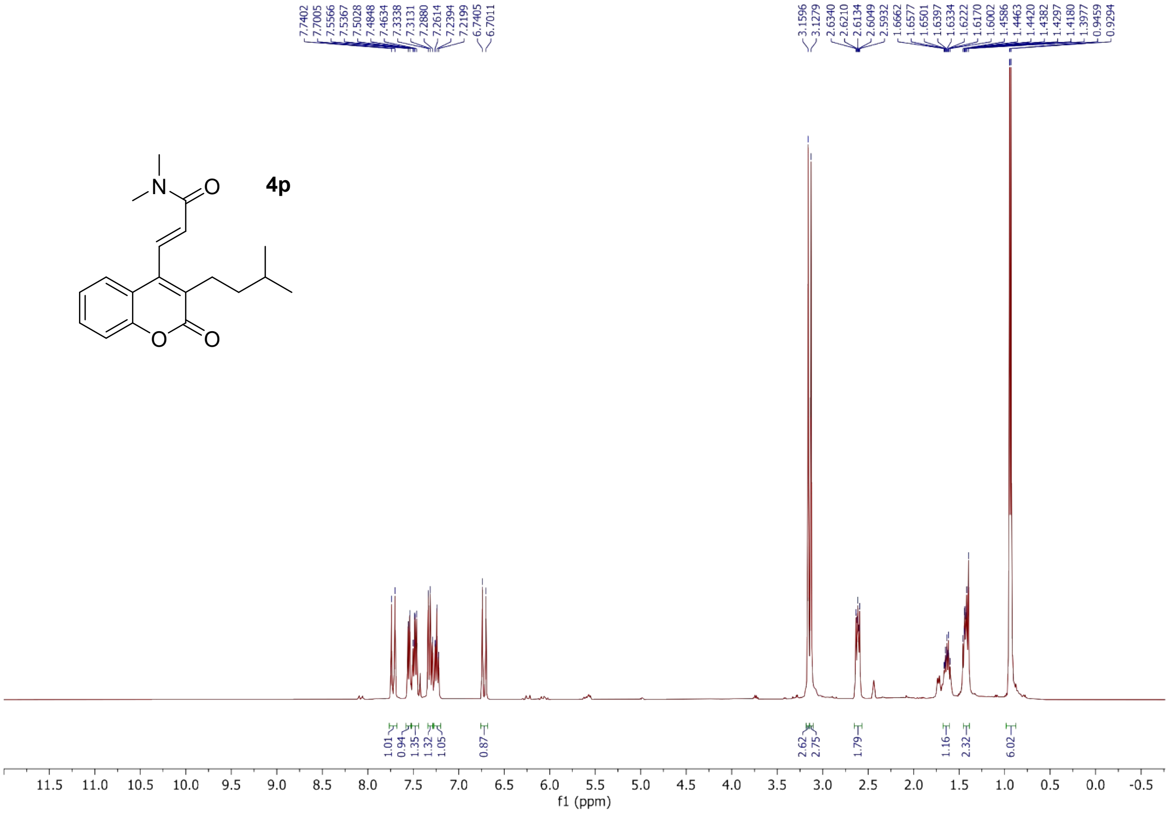

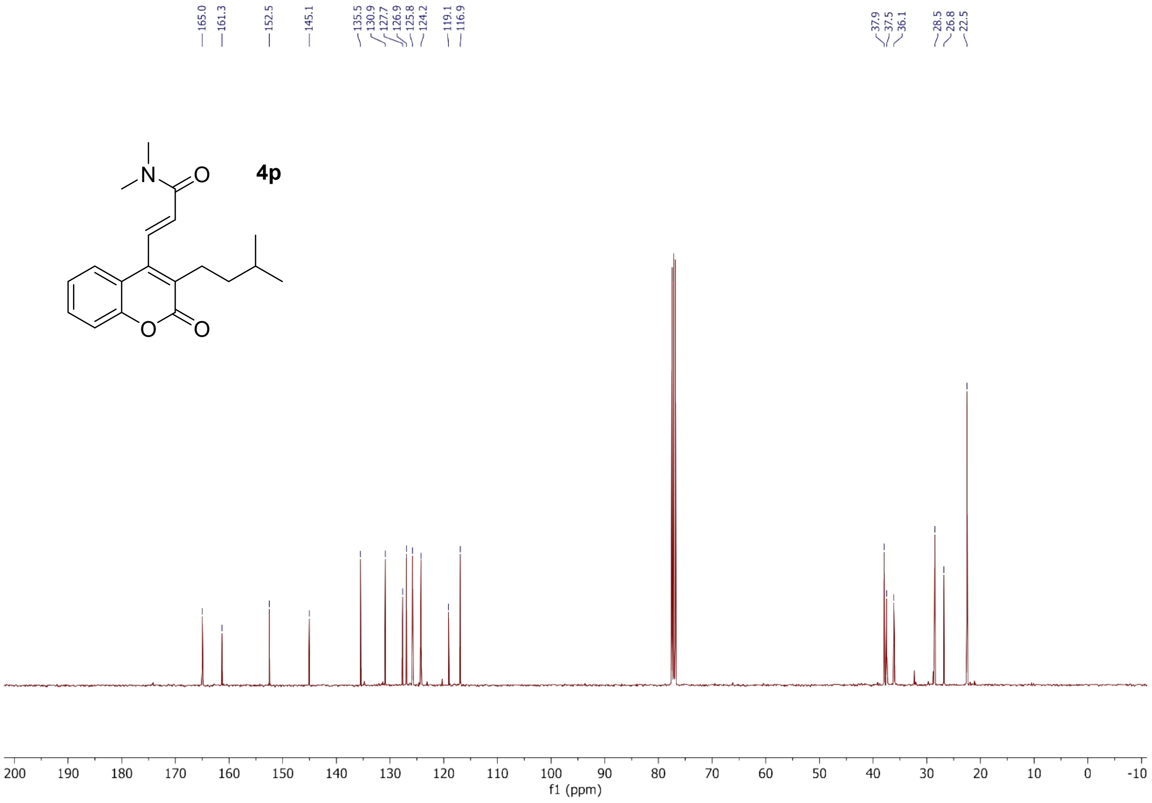

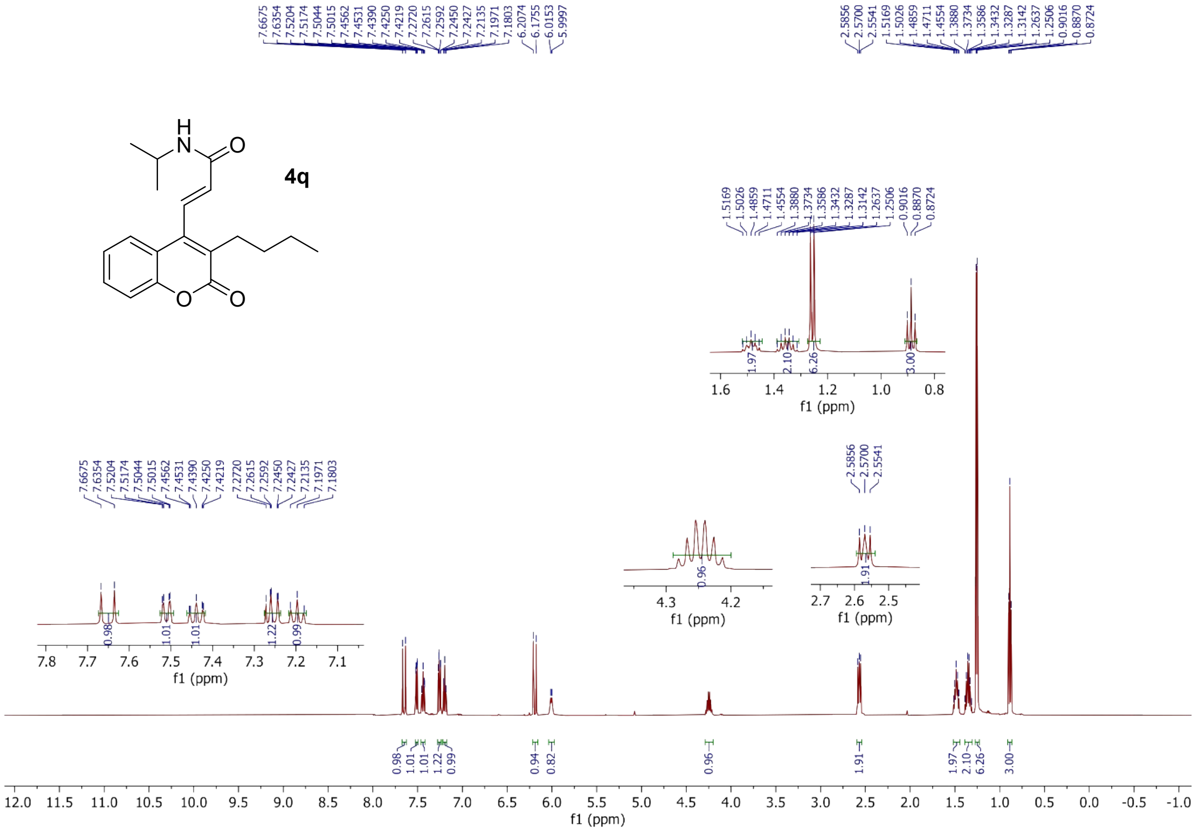
4q
7.6675 7.6354 7.5204 7.5174 7.5044 7.5015 7.4562 7.4531 7.4390 7.4250 7.4219 7.2720 7.2615 7.2592 7.2450 7.2427 7.2135 7.1971 7.1803 6.2074 6.1755 6.0153 5.9997
2.5856 2.5700 2.5541 1.5169 1.5026 1.4859 1.4711 1.4554 1.3880 1.3734 1.3586 1.3432 1.3287 1.3142 1.2637 1.2506 0.9016 0.8870 0.8724
0.98 1.01 1.01 1.22 0.99 0.94 0.82 0.96 1.91 1.97 2.10 6.26 3.00
f1 (ppm)

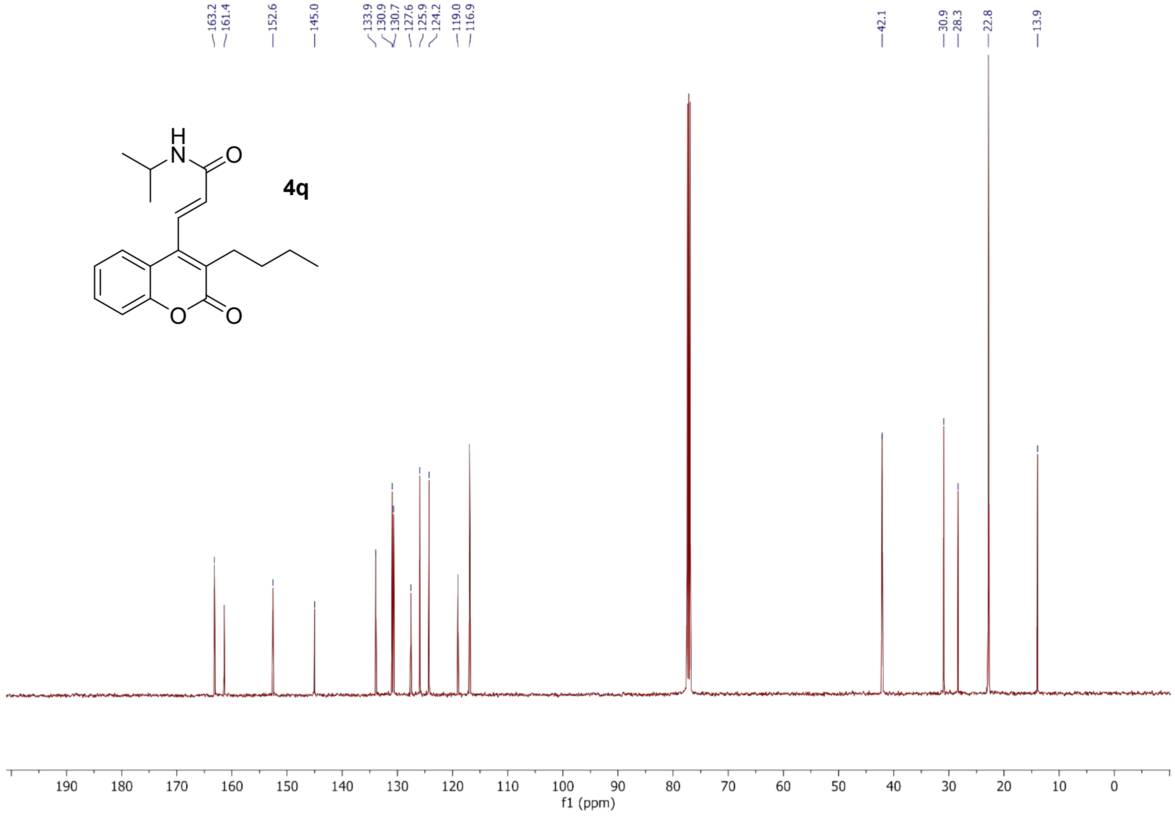
4q
163.2 161.4 152.6 145.0 133.9 130.9 130.7 127.6 125.9 124.2 119.0 116.9 42.1 30.9 28.3 22.8 13.9
f1 (ppm)

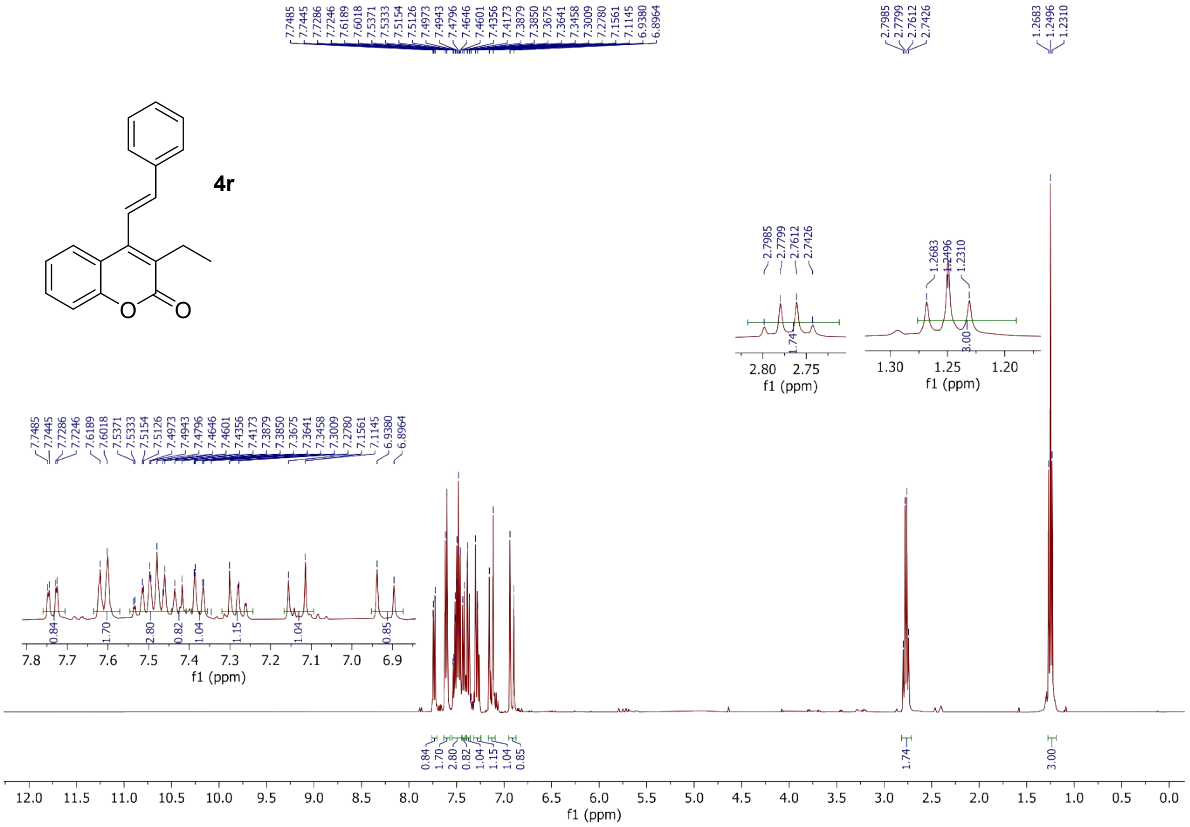

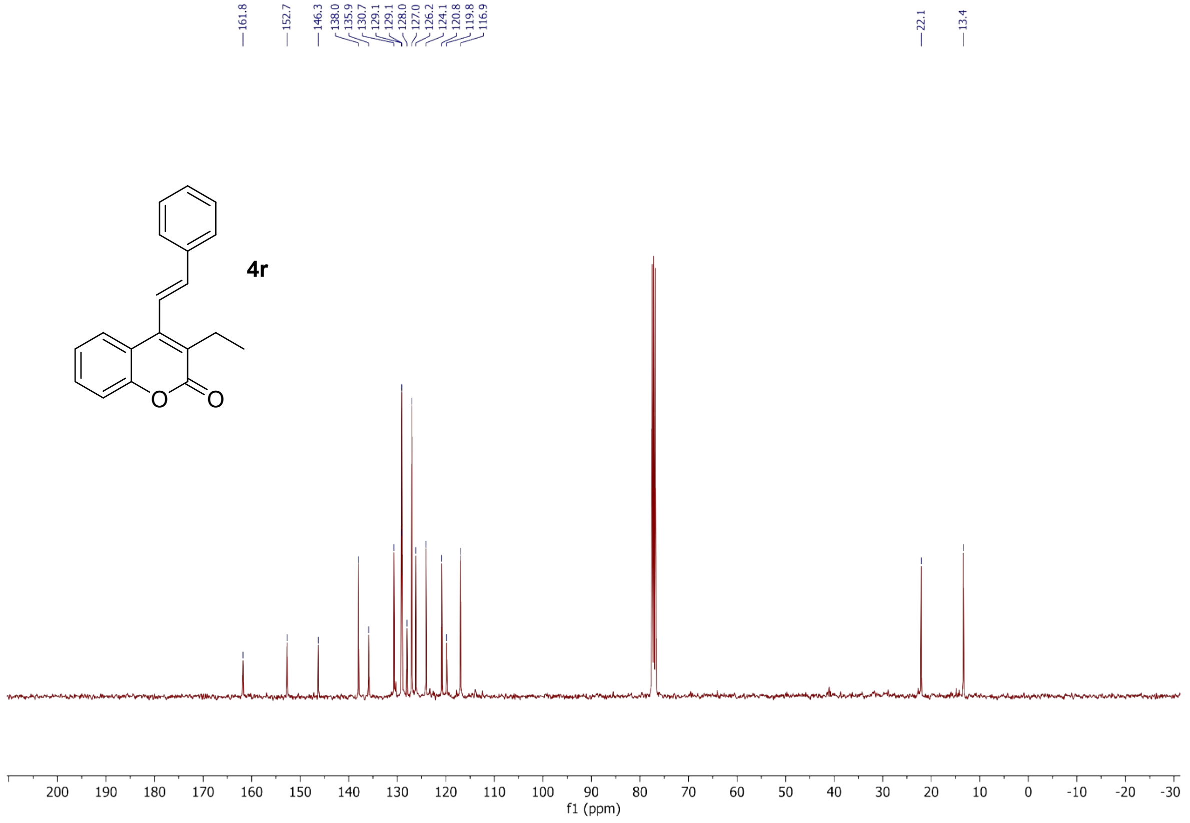

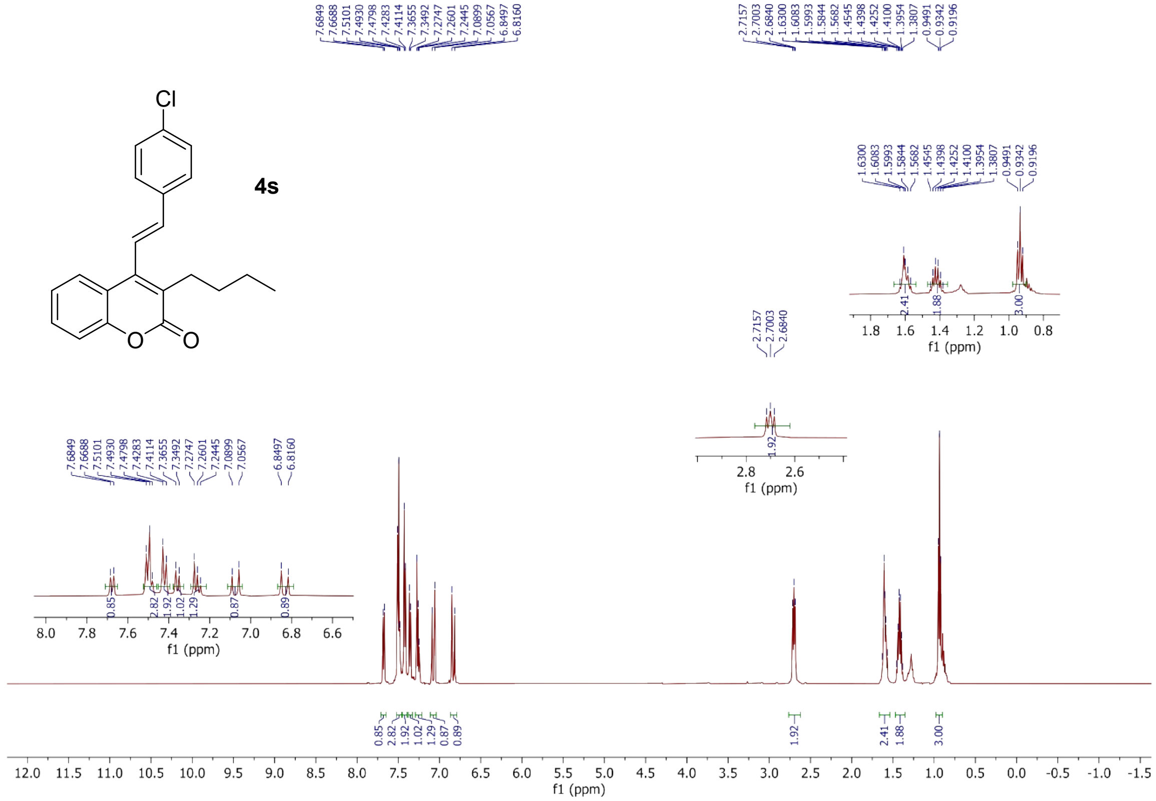
4s
f1 (ppm)

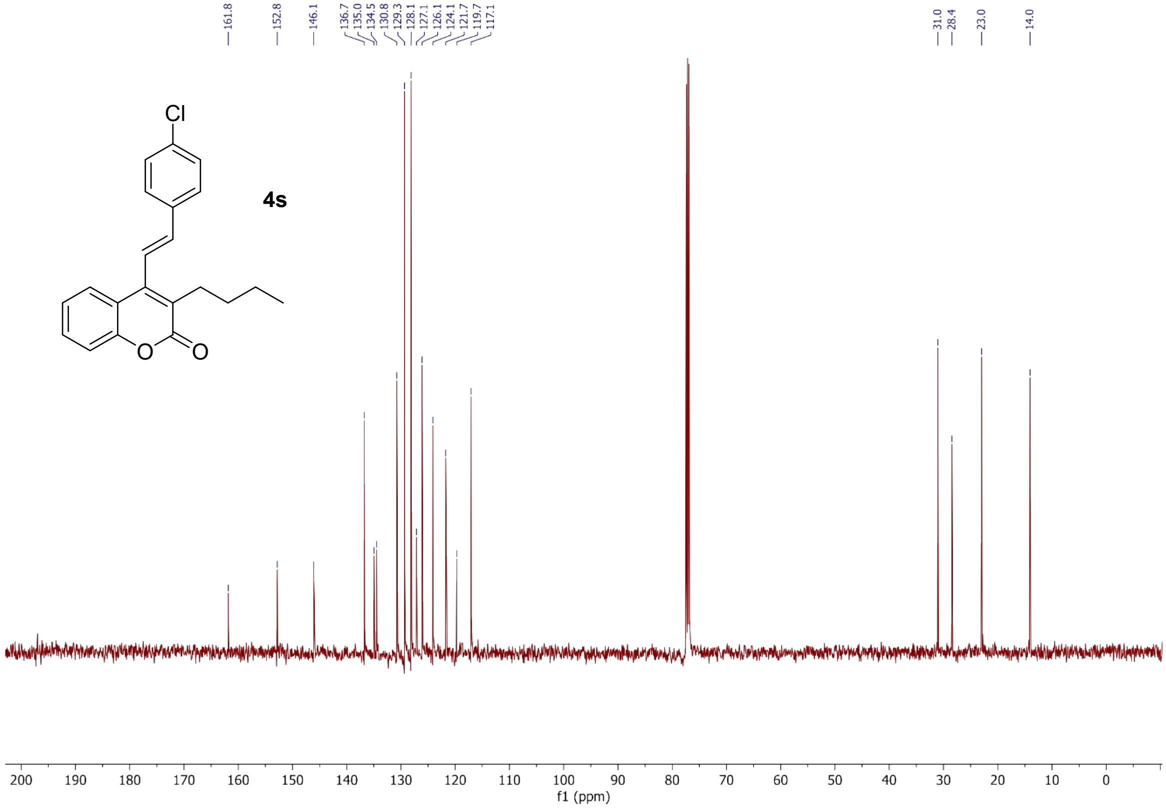
4s
f1 (ppm)

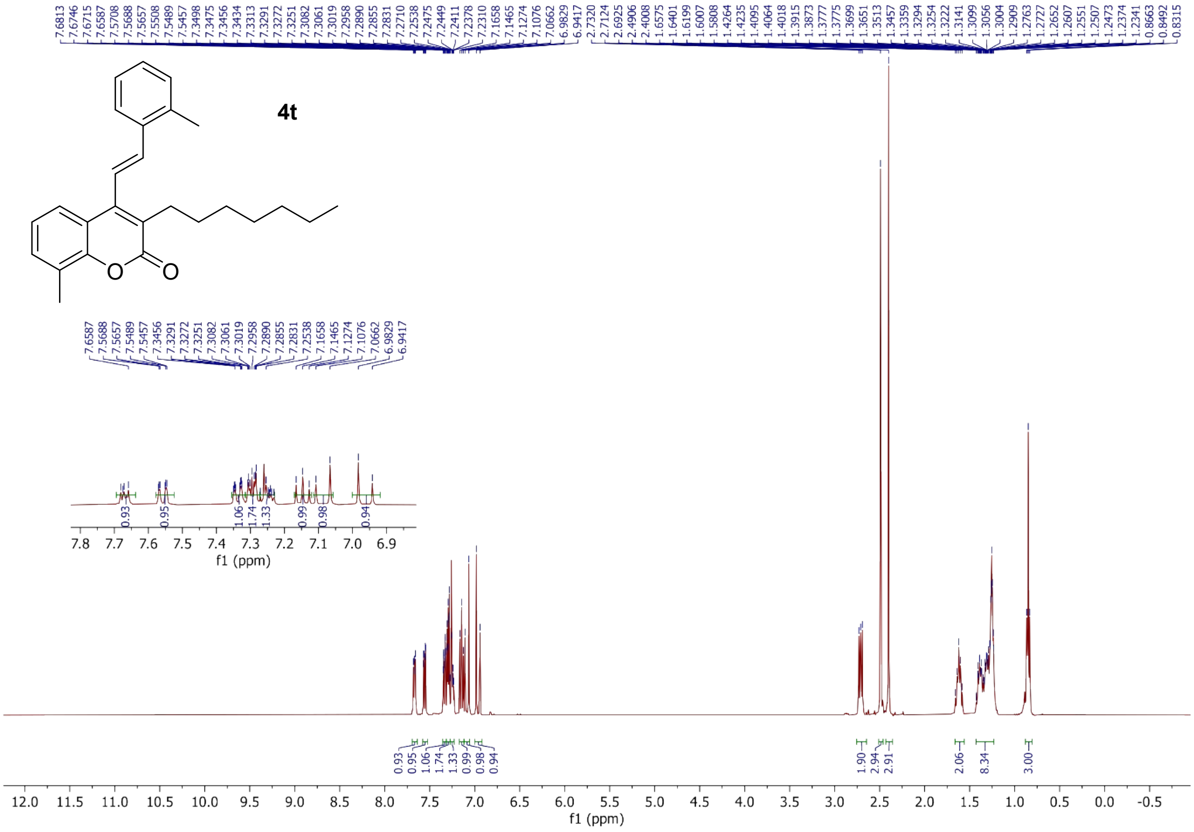

4t
f1 (ppm)

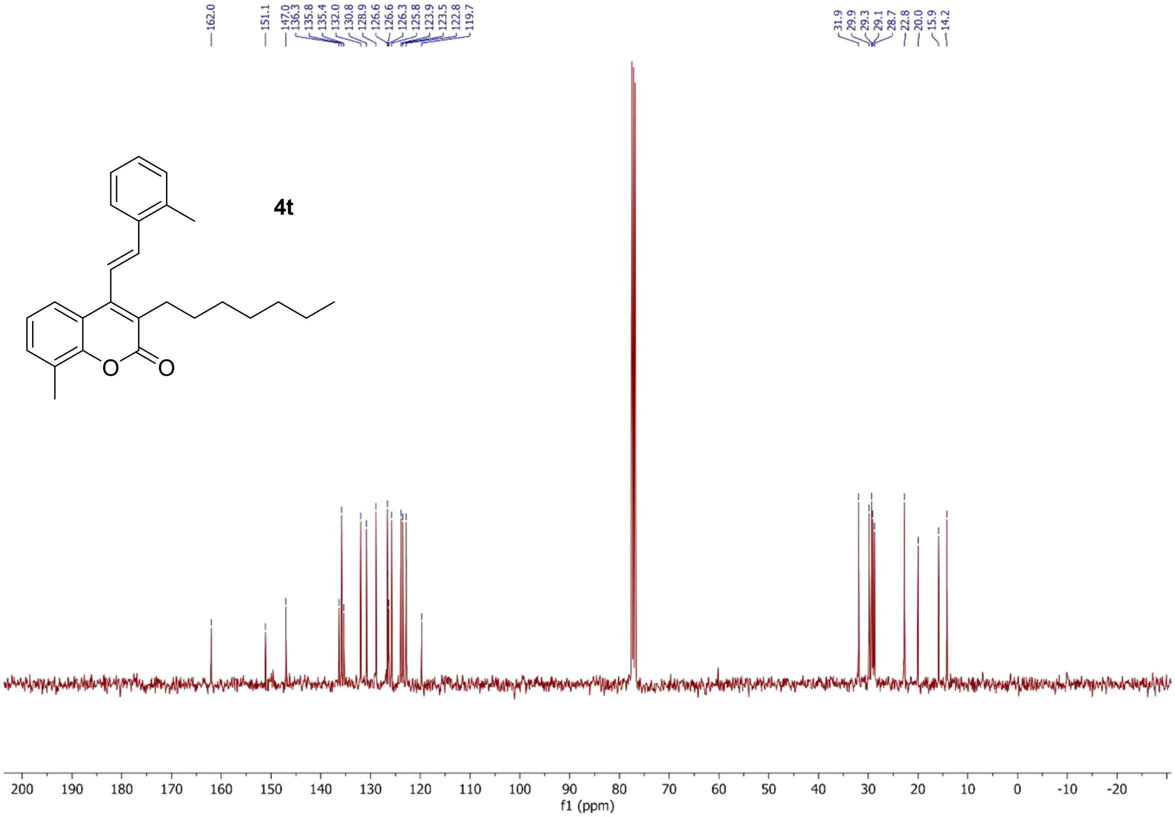

4t
f1 (ppm)

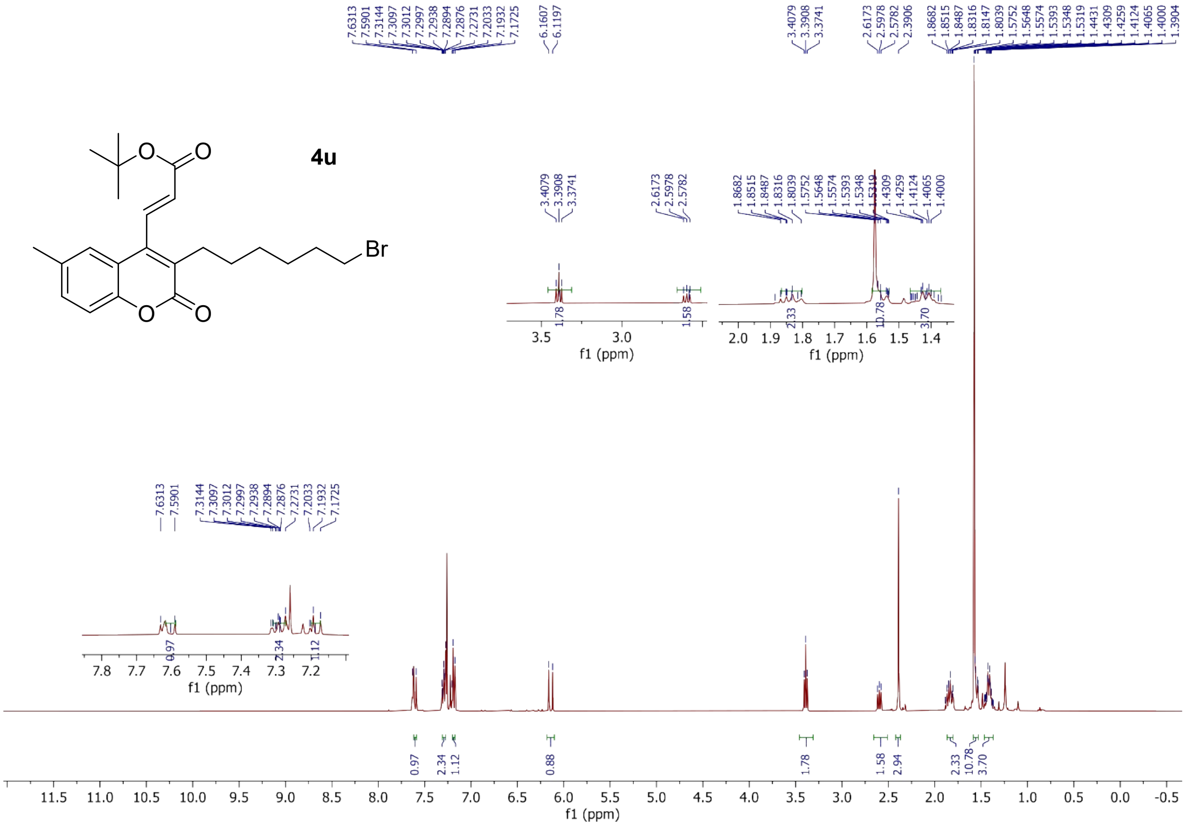
7.6313
7.5901
7.3144
7.3097
7.3012
7.2997
7.2938
7.2894
7.2876
7.2731
7.2033
7.1932
7.1725
6.1607
6.1197
3.4079
3.3908
3.3741
2.6173
2.5978
2.5782
2.3906
1.8682
1.8515
1.8487
1.8316
1.8147
1.8039
1.5752
1.5648
1.5574
1.5393
1.5348
1.5319
1.4431
1.4309
1.4259
1.4124
1.4065
1.4000
1.3904
4u
Br
f1 (ppm)

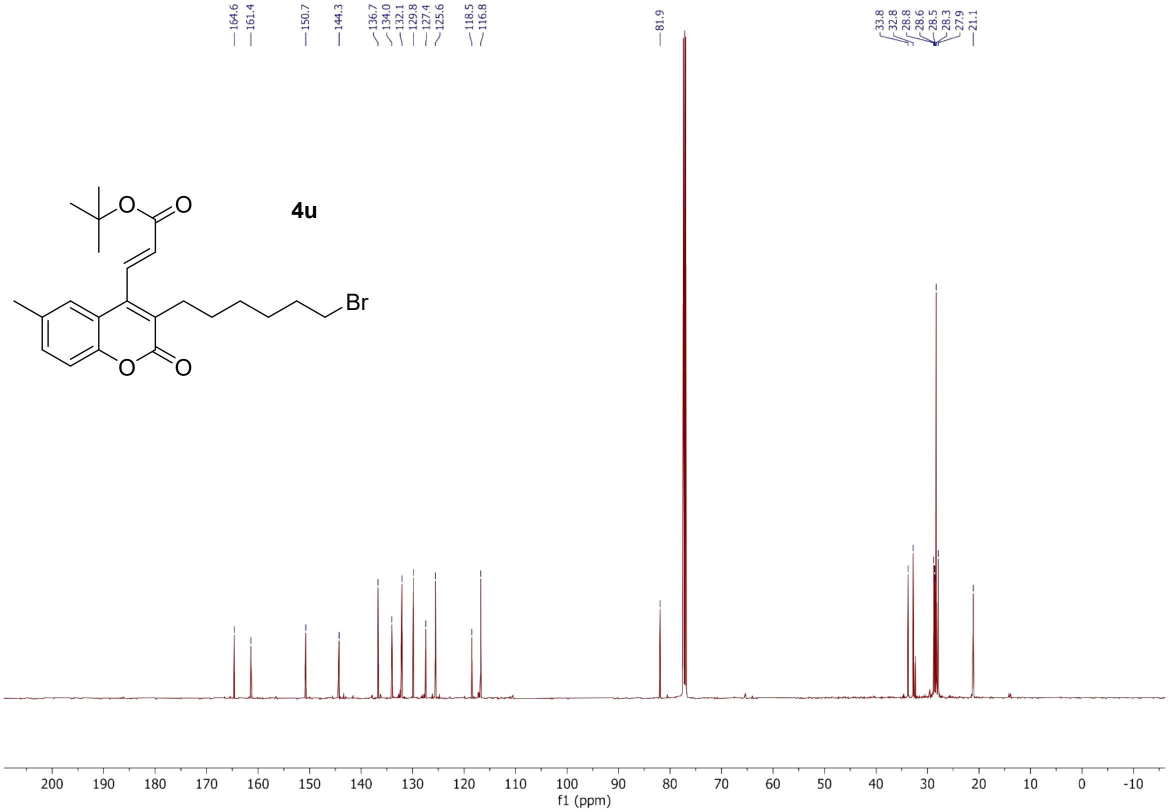
164.6
161.4
150.7
144.3
136.7
134.0
132.1
129.8
127.4
125.6
118.5
116.8
81.9
33.8
32.8
28.8
28.6
28.5
28.3
27.9
21.1
4u
Br
f1 (ppm)

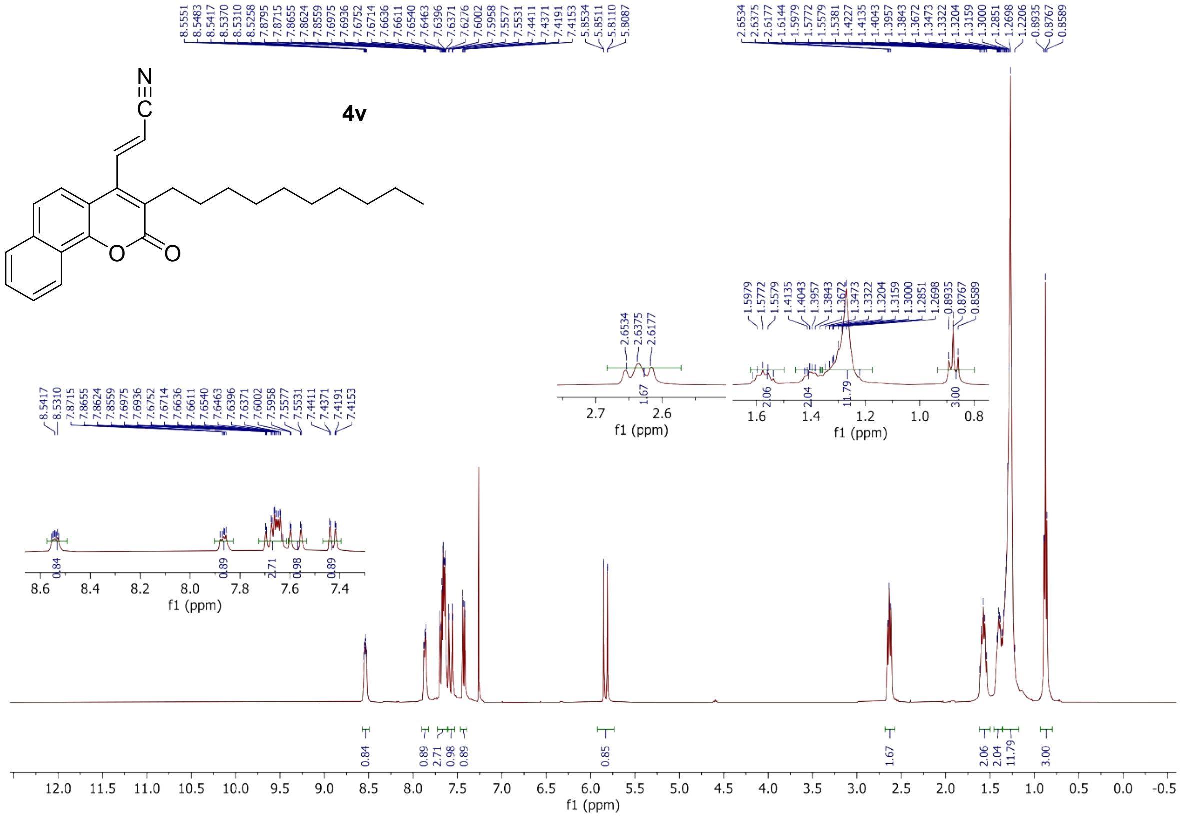
4v
f1 (ppm)

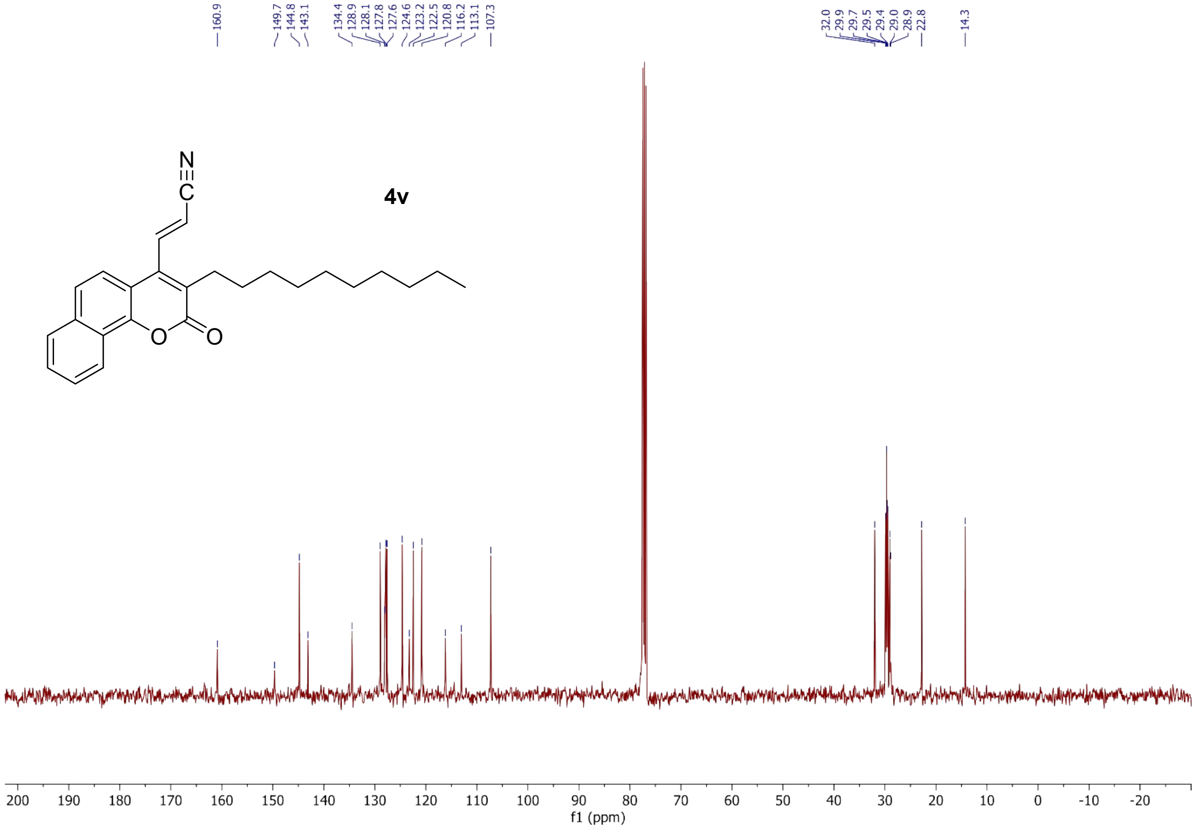
4v
f1 (ppm)

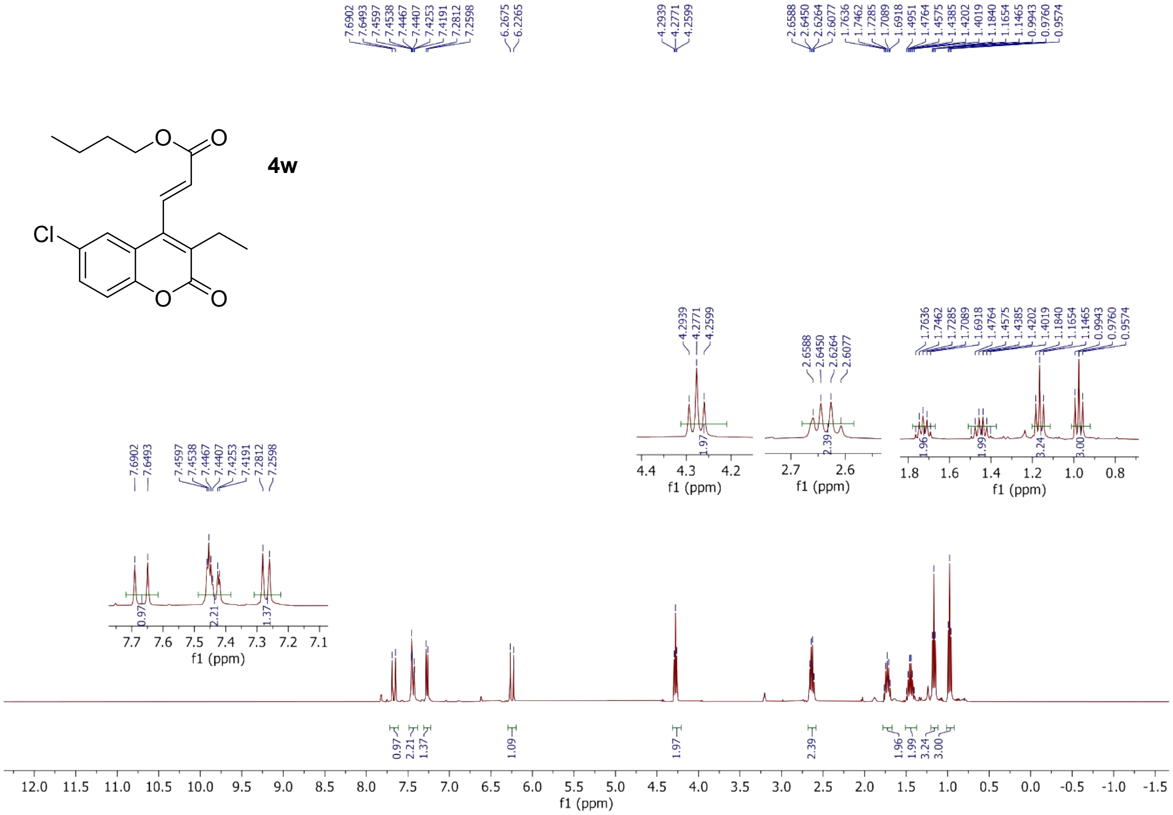
4w
Cl
O
f1 (ppm)

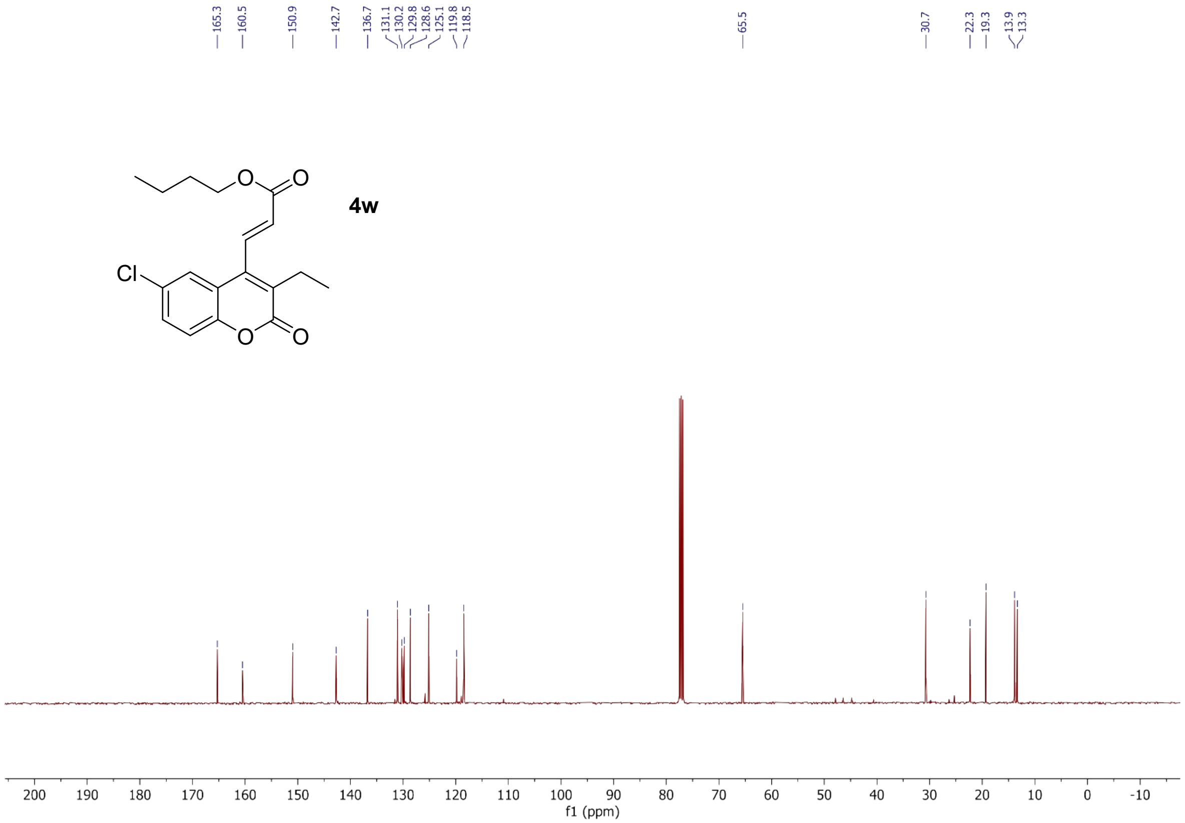
4w
Cl
O
f1 (ppm)

7. References

(1) Balalas, T.; Abdul-Sada, A.; Hadjipavlou-Litina, D.; Litinas, K. Pd-Catalyzed Efficient Synthesis of Azacoumestans Via Intramolecular Cross Coupling of 4-(Arylamino)Coumarins in the Presence of Copper Acetate under Microwaves. *Synthesis* **2017**, *49* (11), 2575–2583.
